# Supplementary material for: STAT1 is required to establish but not maintain interferon‐γ‐induced transcriptional memory
Source: EMBO J. 2023 Jun 5;42(14):e112259. doi: 10.15252/embj.2022112259 (PMC10350821; doi:10.15252/embj.2022112259)
Supplement: Supplementary file 1 — Appendix [file EMBJ-42-e112259-s006.pdf]

Appendix

STAT1 is required to establish but not maintain interferon-γ-induced transcriptional memory

Sahar S.H. Tehrani[1,2], Pawel Mikulski[1], Izma Abdul-Zani[1], João F. Mata[2], Wojciech Siwek[1,3,†] and Lars E.T. Jansen[1*]

[1]Department of Biochemistry, University of Oxford, Oxford, UK

[2]Instituto Gulbenkian de Ciência, Oeiras, Portugal

[3]Present address: Department of Molecular Biology, Massachusetts General Hospital, Harvard Medical School, Boston, MA 02114, USA

†Co-corresponding author: *siwek@molbio.mgh.harvard.edu*

*Correspondence: *lars.jansen@bioch.ox.ac.uk* (lead contact)

Table of Contents

Appendix Figure S1 2
Appendix Figure S2 3
Appendix Table S1. list of gRNAs and primes used in this study. 4

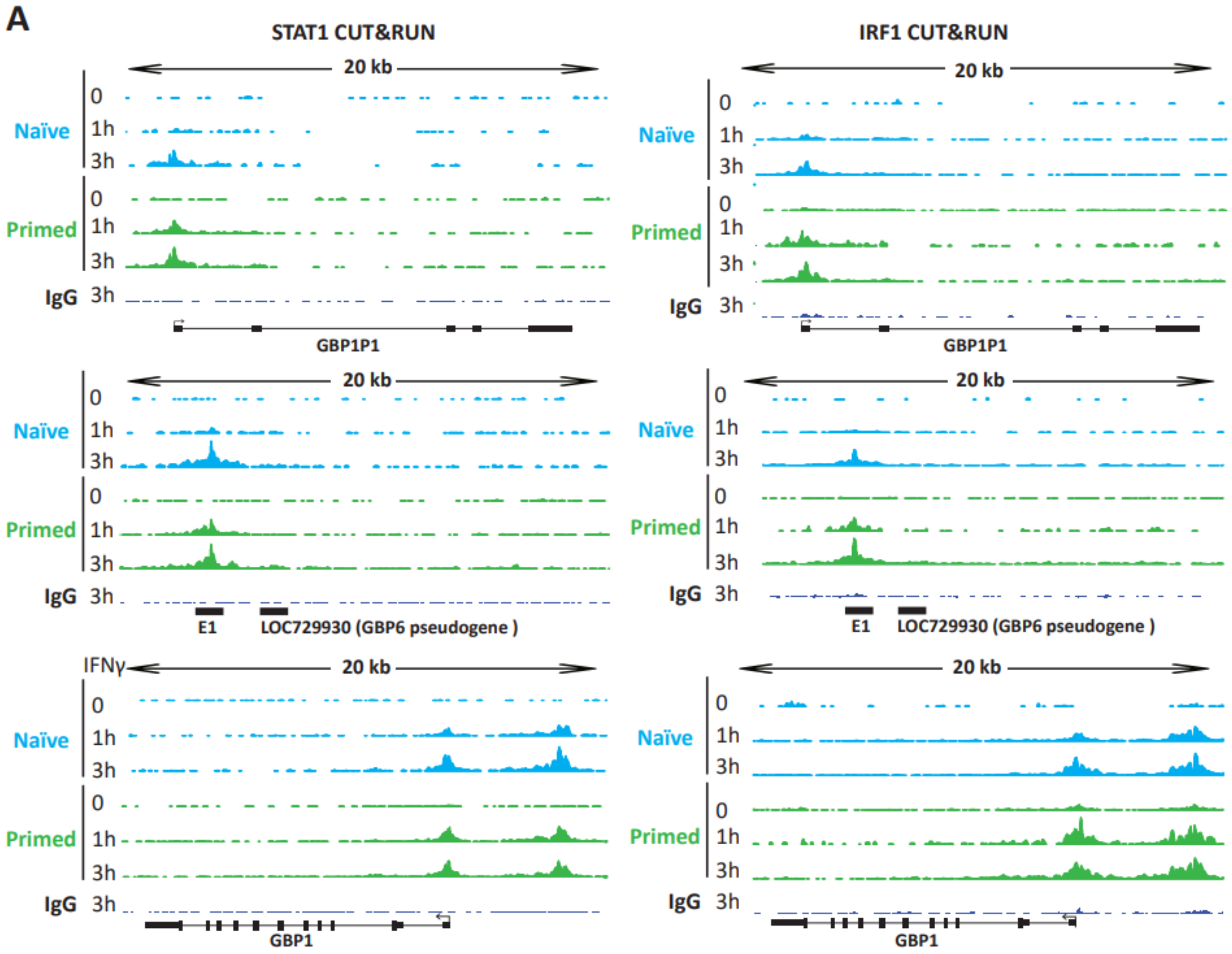

B list of overlap genes in Figure 3G, that showed both faster STAT1 and IRF1 recruitment

	Ensembl	Gene name	description
1	ENSG00000225492	GBP1P1	guanylate binding protein 1 pseudogene
2	ENSG00000154451	GBP5	guanylate binding protein 5 (see Figure 3)
3	ENSG00000162654	GBP4	guanylate binding protein 4 (see Figure 3)
4	ENSG00000237568	nan	LncRNA inside GBP5 gene
5	ENSG00000238081	LOC729930	GBP6 pseudogene, transcription factor binding site is found 2.4 kbp upstream of this site
6	ENSG00000284734	GBP4 (antisense)	novel transcript, antisense to GBP4 (see Figure 3)
7	ENSG00000117228	GBP1	guanylate binding protein 1
8	ENSG00000269588	LGALS13	lectin, galactoside-binding, soluble, 13 (LGALS13) pseudogene
9	ENSG00000253838	nan	lncRNA inside IDO1
10	ENSG00000262151	CIITA (antisense)	novel transcript, antisense to CIITA
11	ENSG00000226025	LGALS17A	galectin 14 pseudogene
12	ENSG00000131203	IDO1	indoleamine 2,3-dioxygenase 1

Appendix Figure S1. Accelerated STAT1 and IRF1 binding in primed genes (A) Representation of processed CUT&RUN data for STAT1 and IRF1 occupancy at the selected GBP genes loci (GBP1P1, LOC729930 and GBP1). **(B)** List of loci showing enhanced STAT1 and IRF1 binding in primed cells as in Figure 3G (−3kb of TSS and +3kb of TTS with a minimum of 1.5-fold difference between primed and naïve upon 1 hour of IFNγ treatment).

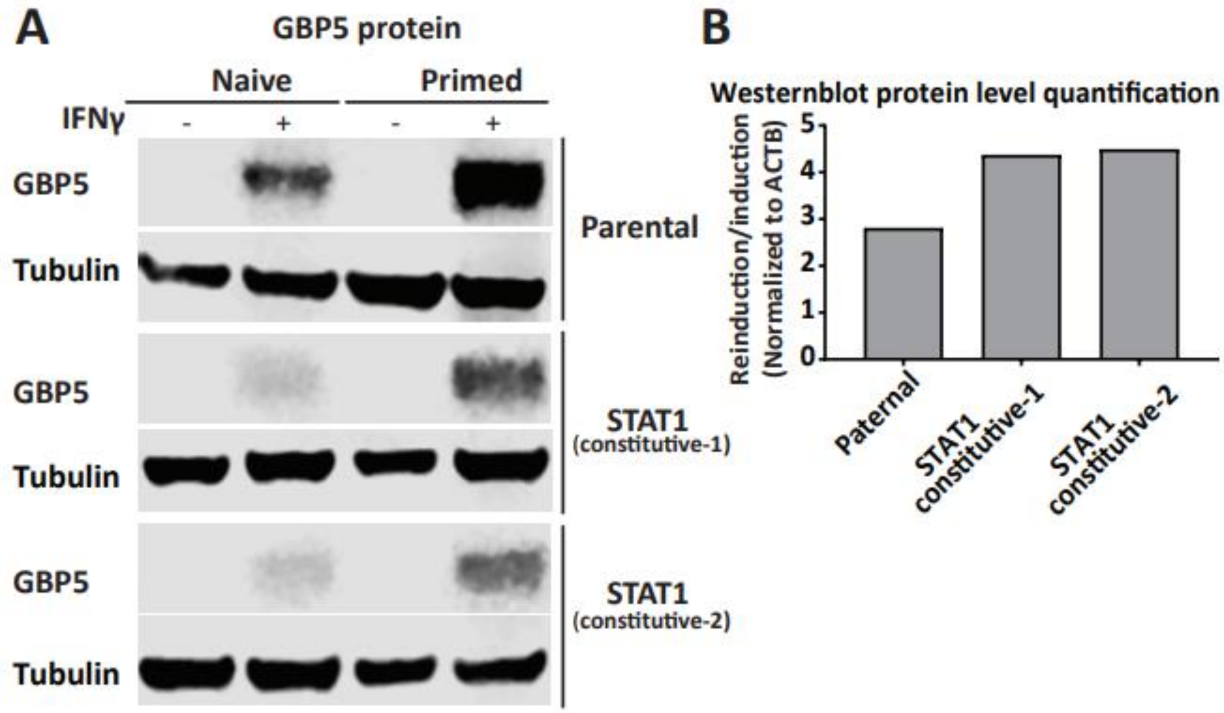

Appendix Figure S2 . Replicates of experiment show in Figure 5D. Two independent clones of STAT1 rescue cells and their parental control were subjected to IFNγ induction and reinduction regime as outlined in Figure 1A. **(A)** Cell extracts were processed for western blotting and probed for GBP5 expression before and after induction and reinduction as indicated in Figure 1A. α-Tubulin (Tubulin) was used as a loading control. Note that GBP5 and Tubulin blot for STAT1 (constitutive-1) are as in Figure 5D, STAT1 (constitutive). **(B)** Western blot signal quantification of blot in 6A. Data are shown as fold change of reinduction to induction.

Appendix Table S1. list of gRNAs and primes used in this study.

**CRISPRa gRNA**	
**Primer name #**	**sequence(5'->3')**
F_GBP1_SAM_gRNA1	CACCGAATTAGTGGAGTGTGCCAG
F_GBP1_SAM_gRNA2	CACCGAAATCTTTAAACCCTCCCAC
F_GBP1_SAM_gRNA3	CACCGTAGAACATGAGTACAACACA
R_GBP1_SAM_gRNA1	AAACCTGGCACACTCCACTAATTC
R_GBP1_SAM_gRNA2	AAACGTGGGAGGGTTTAAAGATTTC
R_GBP1_SAM_gRNA3	AAACTGTGTTGTACTCATGTTCTAC
F_ASCL1_gRNA1	CACCGCGGGAGAAAGGAACGGGAGG
R_ASCL1_gRNA1	AAACCCTCCCGTTCCTTTCTCCCGC
**CRISPR/Cas9**	
**Primer name #**	**sequence(5'->3')**
IRF1B_F	CACCGCATGGCTGGGACATCAACA
IRF9B_F	CACCGAGGGCTCAGCAACATCCATG
STAT1B_F	CACCGAGAACACGAGACCAATGGTG
STAT2B-F	CACCGAAGAATAGCATGGTAGCCT
STAT3A-F	CACCGCTACAGTGACAGCTTCCCAA
STAT5BA-F	CACCGTGCGGCATTATTTATCCCAG
ContorlA-F	CACCGTATTACTGATATTGGTGGG
ControlB-F	CACCGTTCCGCGTTACATAACTTA
IRF1B_R	AAACTGTTGATGTCCCAGCCATGC
IRF9B_R	AAACCATGGATGTTGCTGAGCCCTC
STAT1B_R	AAACCACCATTGGTCTCGTGTTCTC
STAT2B-R	AAACAGGCTACCATGCTATTCTTC
STAT3A-R	AAACTTGGGAAGCTGTCACTGTAGC
STAT5BA-R	AAACCTGGGATAAATAATGCCGCAC
ContorlA-R	AAACCCCACCAATATCAGTAATAC
ControlB-R	AAACTAAGTTATGTAACGCGGAAC
**qPCR**	
Primer name #	sequence(5'->3')
GBP1_qPCR_F	GTGGAACGTGTGAAAGCTGA
GBP1_qPCR_R	CAACTGGACCCTGTCGTTCT
GBP5_trancC_F	TTCAATTTGCCCCGTCTGTG
GBP5_trancC_R	AGGCAGTGTTTCAAGTTGGG
b-actin Forward	AACTGGAACGGTGAAGGTGACAGC
b-actin Reverse	TGGCTTTTAGGATGGCAAGGGAC
