# Supplementary material for: STAT1 is required to establish but not maintain interferon‐γ‐induced transcriptional memory
Source: EMBO J. 2023 Jun 5;42(14):e112259. doi: 10.15252/embj.2022112259 (PMC10350821; doi:10.15252/embj.2022112259)
Supplement: Supplementary file 2 — Expanded View Figures PDF [file EMBJ-42-e112259-s010.pdf]

Expanded View Figures

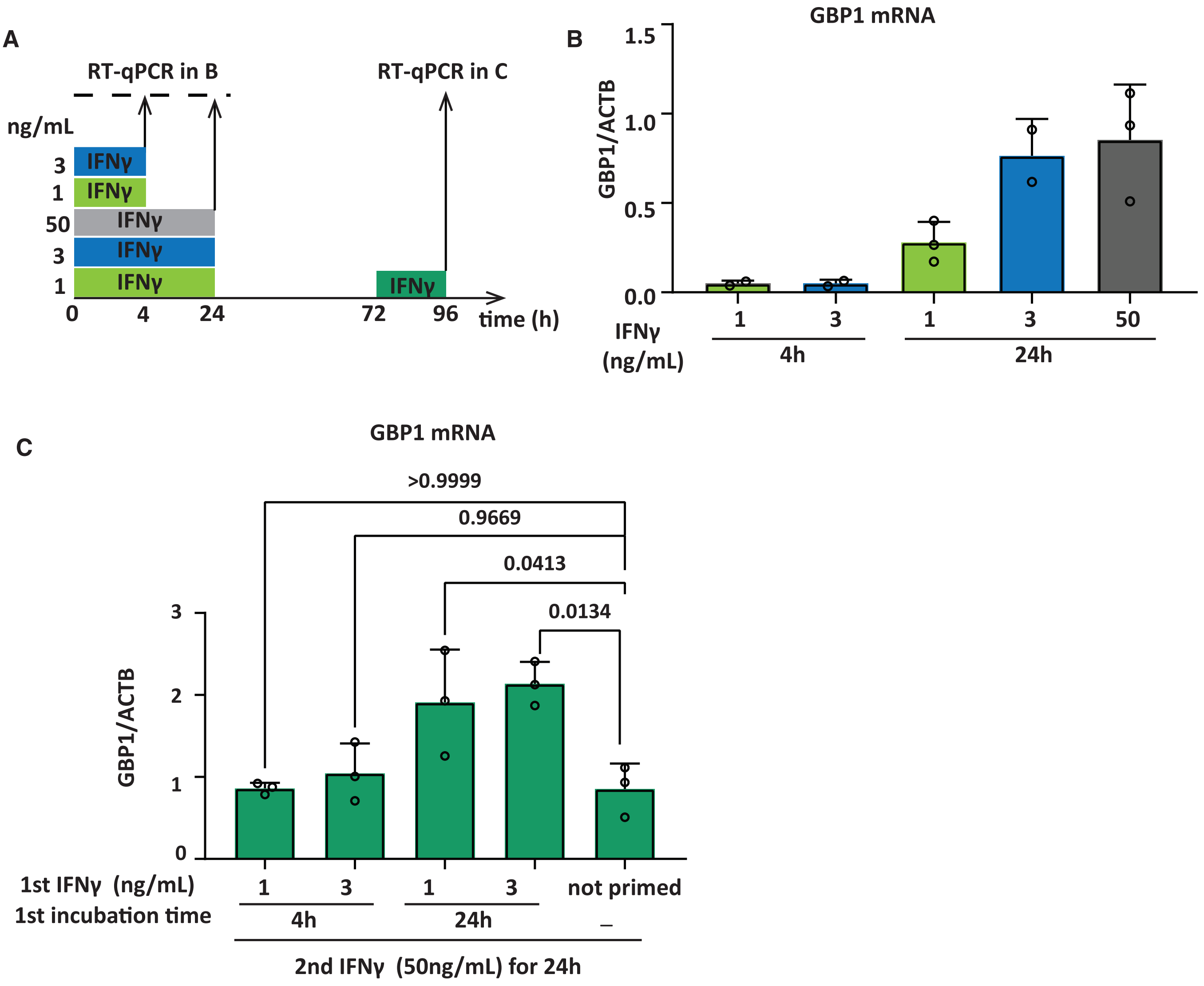

Figure EV1. Titration of minimal IFNγ pulse to prime GBP1.

A Experimental outline of GBP1 priming with different concentrations of IFNγ and incubation times. HeLa cells were induced with 1, 3, or 50 ng/ml of IFNγ for 4 and 24 h, followed by IFNγ washout.

B, C After 48 h, naïve and primed cells were induced with IFNγ (50 ng/ml) for 24 h and harvested for GBP1 mRNA analysis by RT–qPCR after induction (B) and reinduction (C), normalized to ACTB mRNA level. Statistical significance was determined using Ordinary one-way ANOVA. Data are shown as mean (error bars, SD; $n = 3$ biological replicates).

Source data are available online for this figure.

© 2023 The Authors

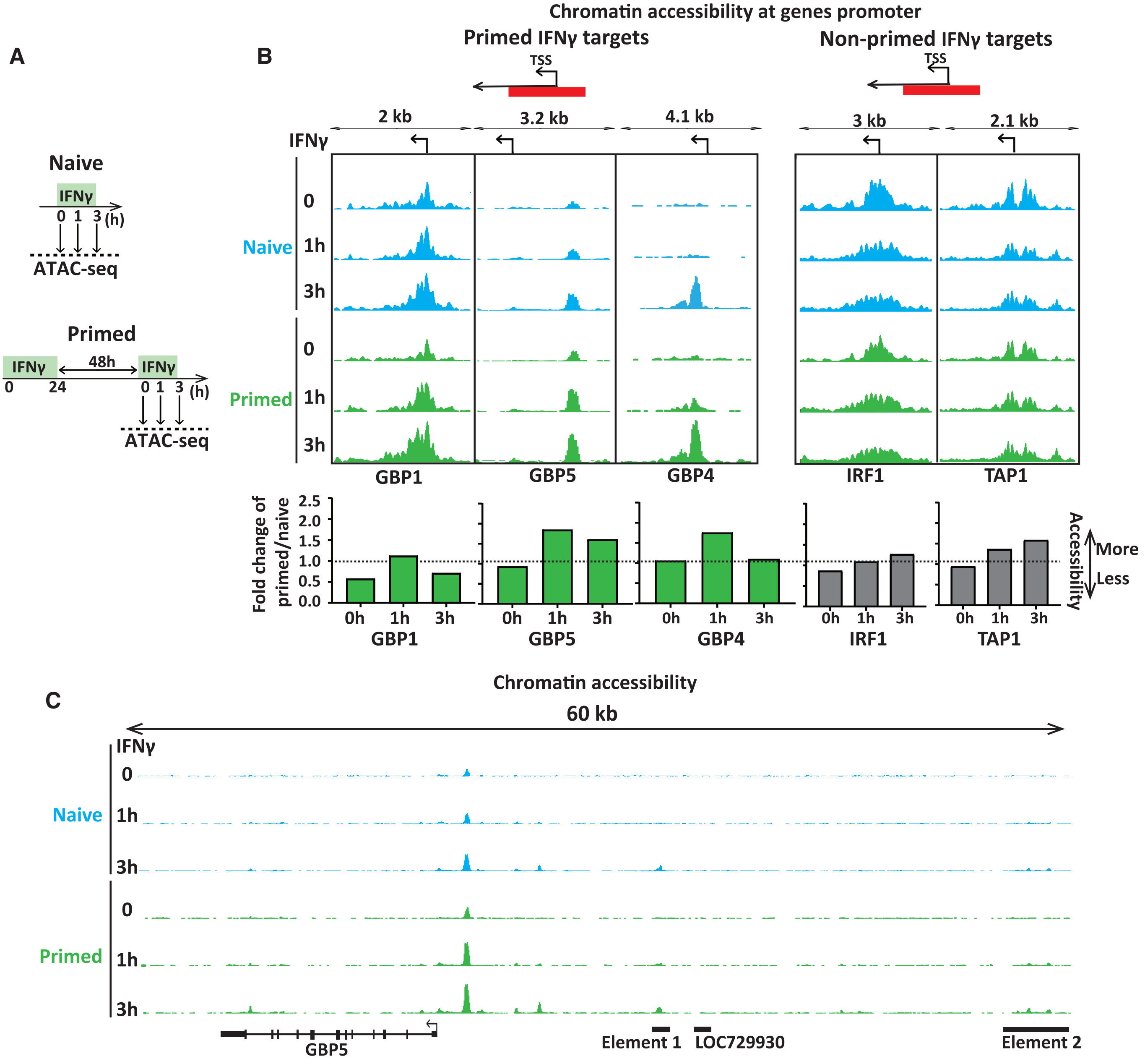

Figure EV2. Promoter accessibility of GBP genes upon IFNγ stimulation.

A Scheme describing chromatin accessibility (ATAC-seq) experiment. HeLa cells were primed with IFNγ for 24 h, followed by IFNγ washout. After 48 h, naïve and primed cells were induced by IFNγ for 1 and 3 h. Cells were harvested at indicated time points and processed for ATAC-seq.

B Results of sequenced reads were mapped to the human genome (hg38), and coverage data is displayed as reads per million (RPM) at equal scaling for two genes showing priming, GBP1, GBP4, and GBP5 (Left) and two IFNγ-induced genes, IRF1 and TAP1 (Right). The data across samples are scaled equally for each locus.

C No nearby enhancers accessible in primed cells. Representation of processed data for ATAC-seq at the GBP5 gene and upstream region. Sequenced reads were mapped to the human genome (hg38), and coverage data are displayed as reads per million (RPM) at equal scaling.

Source data are available online for this figure.

© 2023 The Authors

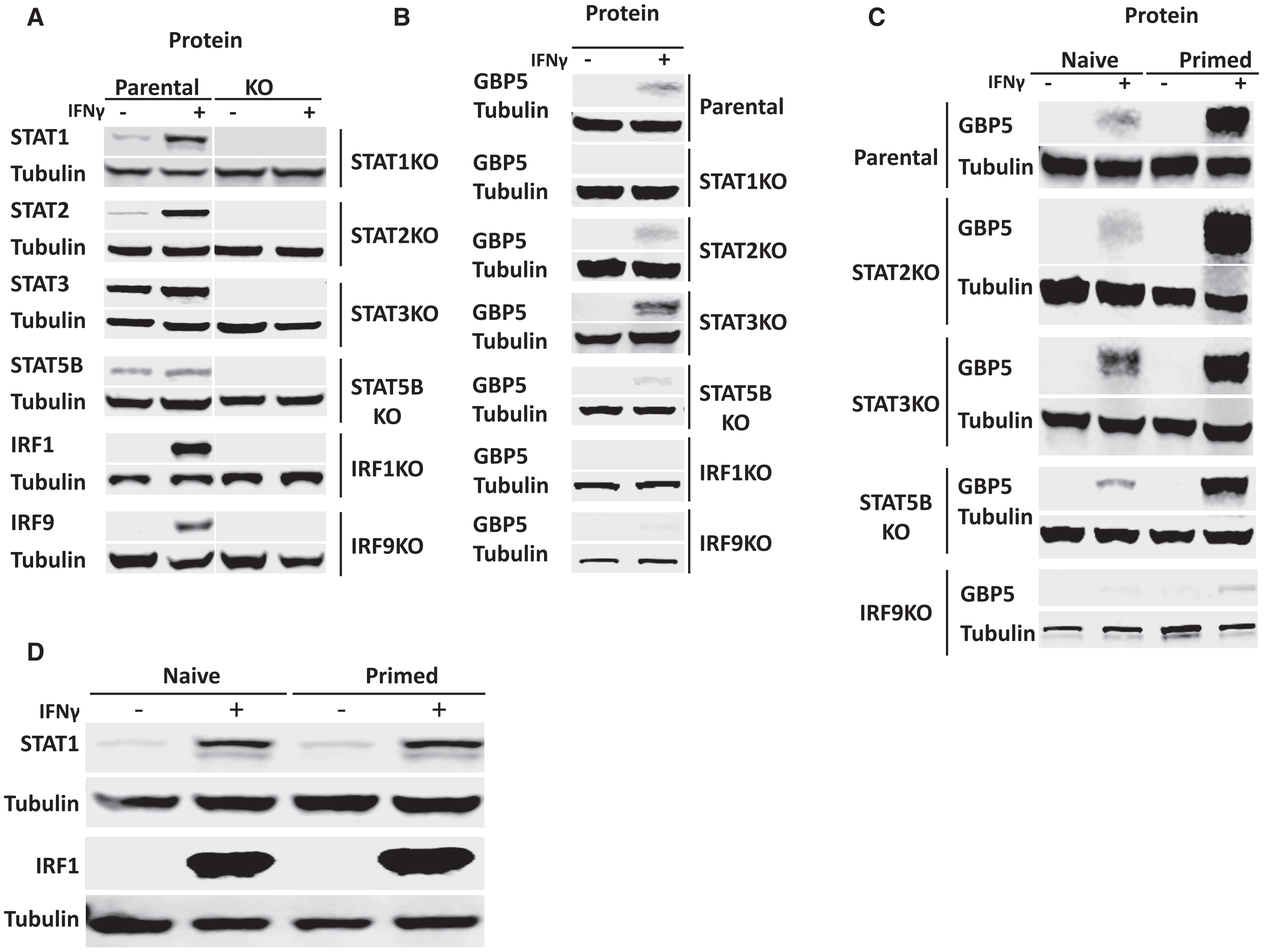

Figure EV3. Knockout cell lines of all relevant STAT and IRF genes to determine the requirement of GBP5 expression.

Stable CRISPR knockouts were generated for indicated genes in HeLa cells. Knockout (KO) cells and their parental controls (WT) were induced with IFNγ for 24 h or left untreated.

A–C Immunoblots probing for (A) STAT and IRF transcription factors to confirm knockout status, (B) probing for the effect on GBP5 expression (C) probing the effect on GBP5 priming. Experiment performed as outlined in Fig 1A. α-Tubulin (Tubulin) was used as a loading control. Note that GBP5 and Tubulin blot for parental cells is as in Fig 1C.

D HeLa cells were subjected to IFNγ induction and reinduction regime as outlined in Fig 1A with 2 days recovery time (primed state) after IFNγ washout. Cell extracts were prepared at indicated time points, processed for western blotting, and probed for STAT1, IRF1. α-Tubulin (Tubulin) as a loading control.

Source data are available online for this figure.

© 2023 The Authors

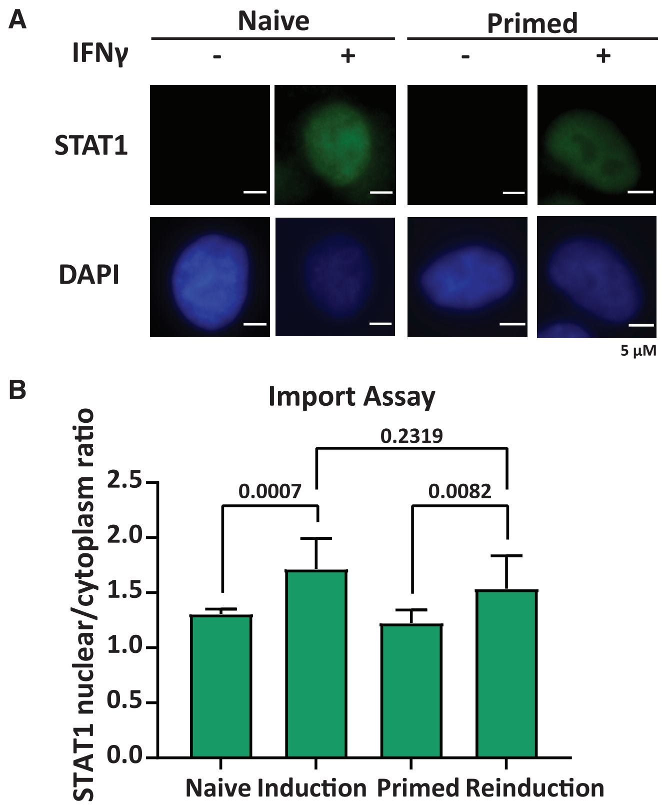

Figure EV4. Priming does not change rate of STAT1 nuclear import.

A Constitutive STAT1 expressing cells were subjected to IFNγ induction and reinduction regime as outlined in Fig 1A. Cells were fixed following indicated treatments as per the scheme in Fig 1A, followed by immunostaining for STAT1 and DAPI and imaging. Scale bar is 5 μm.

B Quantification of the ratio of STAT1 in the nucleus over cytoplasm in fixed cells. Statistical significance was determined using Ordinary one-way ANOVA. Data are shown as mean (error bars, SD; n = 10, biological replicates).

Source data are available online for this figure.

 © 2023 The Authors

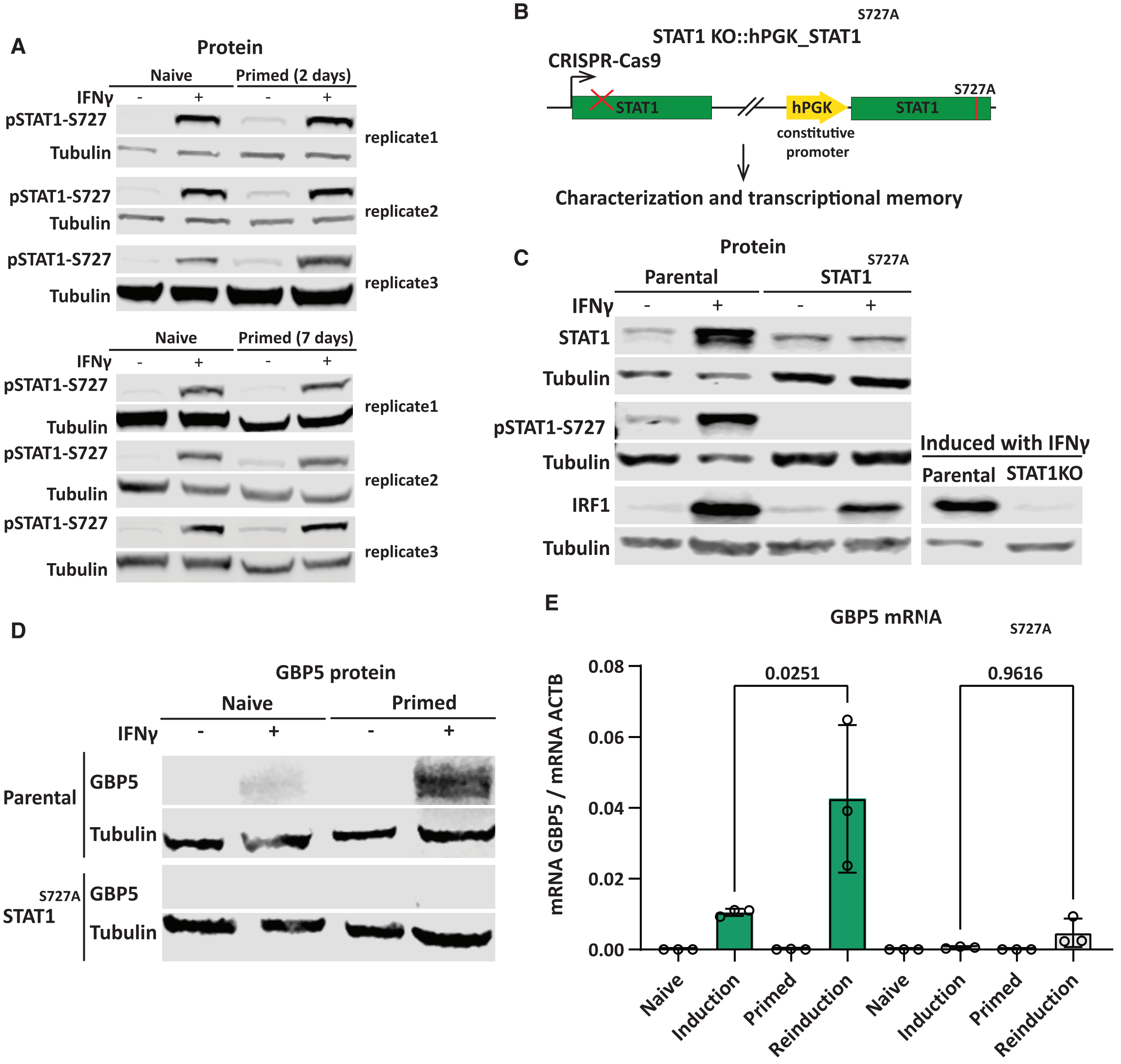

Figure EV5. STAT1 phosphorylation at Ser727 is essential for GBP5 expression.

A Biological replicates of experiments shown in Fig 6A, blots probed for pSTAT1-S727 and α-Tubulin (Tubulin) as a loading control.

B Schematic overview of STAT1 KO cell line rescued with a STAT1 variant with an S727A mutation under a constitutive promoter.

C STAT1KO::STAT1-S727A expressing cells or their parental controls (WT) were induced with IFNγ for 24 h or left untreated and processed for western blotting. Extracts were probed for STAT1, pSTAT1-S727, and IRF1 to confirm the mutation and to assess STAT1S727A function.

D STAT1-S727A expressing cells or their parental controls (WT) were subjected to IFNγ induction and reinduction regime as outlined in Fig 1A, and cell extracts were immunoblotted to determine the GBP5 protein level. α-Tubulin (Tubulin) was used as a loading control.

E In parallel to (D), RNA was isolated and GBP5 mRNA level was determined by RT–qPCR and normalized to ACTB mRNA level. Statistical significance was determined using Ordinary one-way ANOVA. Data are shown as mean (error bars, SD; $n = 3$ biological replicates).

Source data are available online for this figure.

© 2023 The Authors
