# Supplementary material for: STAT1 is required to establish but not maintain interferon‐γ‐induced transcriptional memory
Source: EMBO J. 2023 Jun 5;42(14):e112259. doi: 10.15252/embj.2022112259 (PMC10350821; doi:10.15252/embj.2022112259)
Supplement: Supplementary file 4 — PDF+ [file EMBJ-42-e112259-s011.pdf]

Article

SOURCE DATA | TRANSPARENT PROCESS | OPEN ACCESS

THE EMBO JOURNAL

STAT1 is required to establish but not maintain interferon-γ-induced transcriptional memory

Sahar SH Tehrani[1,2], Pawel Mikulski[1], Izma Abdul-Zani[1], João F Mata[2], Wojciech Siwek[1,*,†] & Lars ET Jansen[1,**]

Abstract

Exposure of human cells to interferon-γ (IFNγ) results in a mitotically heritable yet reversible state called long-term transcriptional memory. We previously identified the clustered GBP genes as strongly primed by IFNγ. Here, we discovered that in primed cells, both interferon-responsive transcription factors STAT1 and IRF1 target chromatin with accelerated kinetics upon re-exposure to IFNγ, specifically at promotors of primed genes. Priming does not alter the degree of IFNγ-induced STAT1 activation or nuclear import, indicating that memory does not alter upstream JAK–STAT signaling. We found STAT1 to be critical to establish transcriptional memory but in a manner that is independent of mere transcription activation. Interestingly, while Serine 727 phosphorylation of STAT1 was maintained during the primed state, STAT1 is not required for the heritability of GBP gene memory. Our results suggest that the memory of interferon exposure constitutes a STAT1-mediated, heritable state that is established during priming. This renders GBP genes poised for subsequent STAT1 and IRF1 binding and accelerated gene activation upon a secondary interferon exposure.

Keywords epigenetic memory; epigenetics; GBPs; STAT1; IRF1; trained immunity
Subject Categories Chromatin, Transcription & Genomics; Immunology
DOI 10.15252/embj.2022112259 | Received 2 August 2022 | Revised 9 May 2023 | Accepted 12 May 2023 | Published online 5 June 2023
The EMBO Journal (2023) 42: e112259

Introduction

The innate immune system, in contrast to adaptive immunity, has classically been considered nonspecific and transient with no memory of prior infections. However, it has become apparent that in some cases, activation of an innate immune response can lead to a primed state that results in enhanced resistance to reinfection, even to a different pathogen (Netea *et al*, 2011; Peignier & Parker, 2020). Such priming can last for weeks or months, is reported to be independent of the adaptive immune system, and is referred to as trained immunity (Netea *et al*, 2011). Examples include exposure of mice that lack functional B and T lymphocytes to BCG vaccine (Bacillus Calmette–Guérin), *Candida albicans*, or β-glucan (a component of the fungal cell wall) that induce a primed response resulting in enhancement of inflammatory cytokine production upon a secondary infection (Kleinnijenhuis *et al*, 2012; Quintin *et al*, 2012). This innate immune priming correlates with molecular changes including chromatin accessibility and modification (Lau *et al*, 2018), transcription of long noncoding RNAs (lncRNAs) (Fanucchi *et al*, 2019), DNA methylation (Verma *et al*, 2017) and reprogramming of cellular metabolism (Natoli & Ostuni, 2019; Netea *et al*, 2020). At present, most of these molecular signatures are correlative, yet understanding the mechanistic basis of “trained immunity” would enable us to exploit this phenomenon for clinical applications such as vaccination, as well as for the prevention and treatment of conditions such as chronic inflammation.

While the molecular drivers of trained immunity remain elusive and correlative, an analogous priming effect can occur at the level of gene expression that may contribute to trained immunity. This effect, called long-term transcriptional memory is observed upon exposure to inflammatory cytokines such as TNF-α and interferons even in nonimmune cells (Gialitakis *et al*, 2010; Light *et al*, 2013; Zhao *et al*, 2020). Transcriptional memory is also observed outside of the mammalian immune system in a variety of species ranging from yeast to plants, allowing organisms to adapt faster to previously encountered environmental stress conditions such as nutrient deprivation (D’Urso & Brickner, 2017), heat (Ding *et al*, 2013; Lämke *et al*, 2016) and cold stress (Song *et al*, 2012).

Possible mechanisms of transcriptional memory can be broadly categorized as “*cis*-acting” constituting factors such as DNA or histones modification, which may be locally inherited through the cell cycle (Moazed, 2011; Quintin *et al*, 2012); and “*trans*-acting” such as soluble transcription factors that initiate and/or maintain the memory of the signal even in its absence, for example, through rewiring of signaling cascades or transcription factor networks (Moazed, 2011).

Several lines of evidence indicate that local cis-regulated factors can contribute to memory. For instance, DNA demethylation has a positive impact on TNF-α-mediated transcriptional memory genes

1 Department of Biochemistry, University of Oxford, Oxford, UK
2 Instituto Gulbenkian de Ciência, Oeiras, Portugal
*Corresponding author. Tel: +48505313995; E-mail: siwek@molbio.mgh.harvard.edu
**Corresponding author. Tel: +441865613428; E-mail: lars.jansen@bioch.ox.ac.uk
†Present address: Department of Molecular Biology, Massachusetts General Hospital, Harvard Medical School, Boston, MA, USA

© 2023 The Authors. Published under the terms of the CC BY 4.0 license

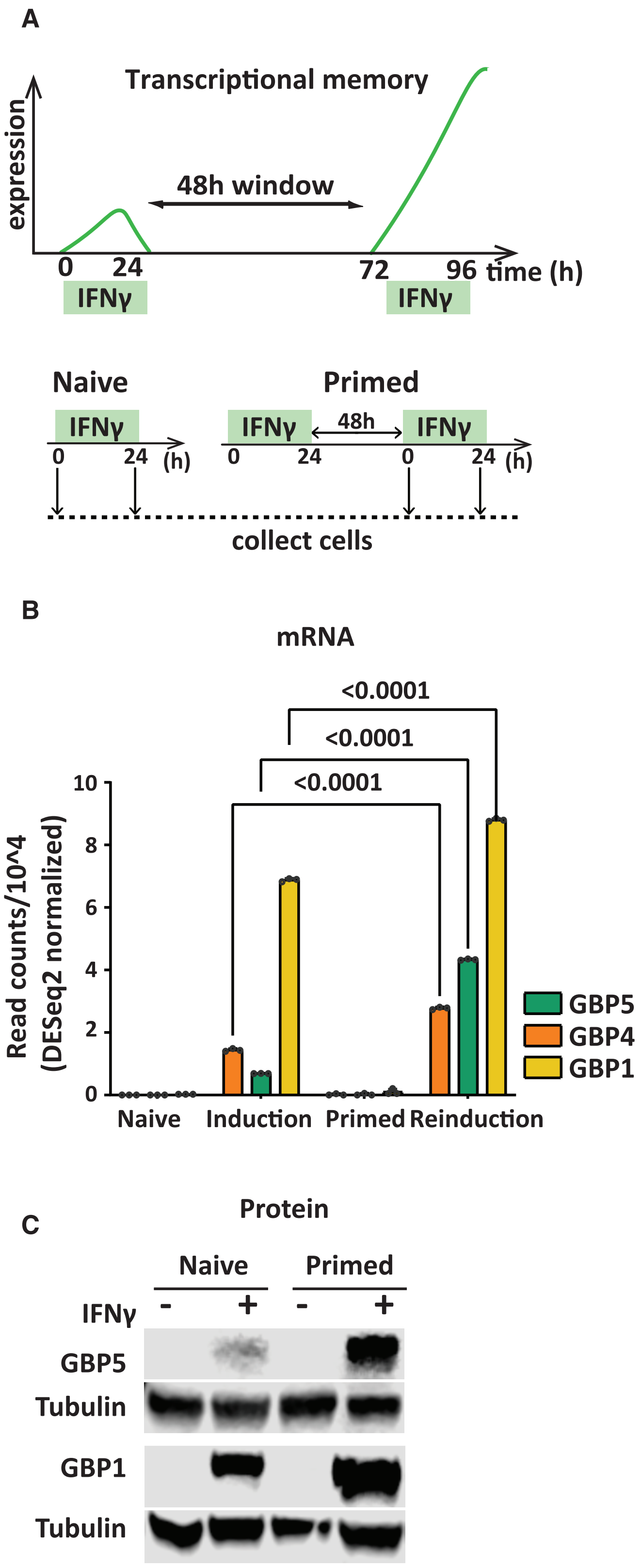

Figure 1.

Figure 1. Long-term transcriptional memory of GBP genes.

A Top: Principle of IFN-induced transcriptional memory. Among IFNγ inducible genes, those with memory show faster and stronger expression upon a second induction with IFNγ. Bottom: Experimental outline for transcriptional memory; HeLa cells were primed with IFNγ for 24 h, followed by IFNγ washout. After 48 h, naïve and primed cells were induced by IFNγ for 24 h.

B RNA-seq data (obtained from dataset reported by Siwek *et al*, 2020). Statistical significance was determined using two-way ANOVA. Data are shown as mean. Error bars, SD; $n = 3$ biological replicates.

C HeLa cells were harvested at indicated time points and processed for western blotting probed for GBP1 and 5 protein levels, α-Tubulin (Tubulin) as a loading control.

Source data are available online for this figure.

(Zhao *et al*, 2020). Additionally, dimethylation of histone H3 on lysine 4 (H3K4me2) has been widely associated with the maintenance of a prime state. For instance in yeast, COMPASS and mediator play a role in maintaining H3K4me2 in the context of the memory of INO1 expression (D'Urso *et al*, 2016). In plants, both H3K4me2 and 3 are implicated in retaining the transcriptional memory of *a prior* stressor such as acquired thermotolerance (Lämke *et al*, 2016). Besides the role of local chromatin factors in transcriptional memory, trans-acting transcription factors have also been implicated in the initiation or maintenance of priming. The yeast transcription factors, Sfl1 and Tup1, are critical for maintaining poised transcription, and the loss of those factors disrupts the transcriptional memory of INO1 and GAL1, respectively (D'Urso *et al*, 2016; Sood *et al*, 2017). Moreover, transcription factor MYC2, which is induced upon dehydration stress and HSFA2 for heat stress, is required for memory (Lämke *et al*, 2016; Liu & Avramova, 2016).

The widespread occurrence of transcriptional memory suggests that some basic principles and mechanisms may underlay this phenomenon and may thus be driven by mechanisms that are not unique to one cell type or system. In the context of the innate immune system, it is striking that cytokine signals such as interferons induce transcriptional memory even in nonimmune cells (Gialitakis *et al*, 2010). This creates an opportunity to discover general principles of transcriptional memory underlying trained immunity without the confounding effects of immune signaling and cell differentiation that can be induced by cytokines.

A well-established paradigm in innate immune transcriptional memory is the priming of genes by interferon-γ (IFNγ). In this case, a subset of IFNγ-activated genes can maintain a heritable poised state in the absence of active transcription of the primed genes. Yet, the primed state leads to an enhanced expression upon re-exposure to IFNγ (Fig 1A). Early studies showed that an IFNγ target gene, HLA-DRA undergoes priming, which correlates with the maintenance of RNA polymerase II (RNA PolII) (Light *et al*, 2013; D'Urso & Brickner, 2017) and H3K4me2 on the promoter of HLA-DRA in primed cells (Gialitakis *et al*, 2010), at least short term up to 48 h post an IFNγ pulse. However, other reports did not detect RNA Pol II poising following the priming of mouse fibroblasts by IFNβ and

© 2023 The Authors

IFNγ or HeLa cells (Kamada *et al*, 2018; Siwek *et al*, 2020). Additionally, H3.3 and H3K36me3 were observed as a memory mark, maintained on primed genes, albeit for a short 2-day period following an IFNγ pulse (Kamada *et al*, 2018; Siwek *et al*, 2020).

We previously reported that genes showing strong interferon-γ induced transcriptional memory tend to reside in genomic clusters and that the long-term memory of these genes is locally restricted by cohesion (Siwek *et al*, 2020). Here we aim to understand the role of transcription in priming and explore the contribution of the STAT and IRF transcription factors in IFNγ-mediated transcriptional memory. We find that in the primed state, the kinetics of the upstream JAK–STAT signaling cascade to activate STAT1 is not altered. Instead, we find that the chromatin of memory target gene promoters is more accessible in primed cells and that STAT1 and IRF1 are recruited faster specifically at the primed GBP cluster. Interestingly, memory is not driven by target gene transcription but depends on a STAT1-dependent state that is established during priming, after which STAT becomes dispensable for the maintenance of the primed state.

Results

GBP genes show long-term transcriptional memory

Exposure of cells to interferon-gamma (IFNγ) leads to a heritable, primed state resulting in enhanced activation of target genes following a second exposure (Gialitakis *et al*, 2010). Using this principle as an assay (outlined in Fig 1A), we previously identified genes encoding the guanylate binding proteins, including GBP1, GBP4, and GBP5 that show mitotically stable memory that is propagated for at least a week in proliferating cells (Siwek *et al*, 2020; Fig 1B). Using a standardized protocol with a 2-day memory window (Fig 1A), we validated these findings by directly measuring protein levels for GBP1 and GBP5 in HeLa cell lines (Fig 1C). In this study, we use GBP genes as a readout of memory, particularly GBP5, 4, and 1 as they showed the strongest reinduction upon a second IFNγ exposure.

Transcription of GBP1 is not sufficient to induce a local prime state

We previously reported that, based on single-cell RNA sequencing that, at least for GBP5, priming is manifested by an increased probability of primed cells to engage in target gene expression, correlating with the strength of the initial GBP5 activation (Siwek *et al*, 2020). Furthermore, earlier work has shown that priming results in enhanced Pol II recruitment or retention of promoter-bound polymerase in the absence of ongoing transcription (Light *et al*, 2013; Kamada *et al*, 2018). This suggests that transcription of the target gene itself may be sufficient to induce the prime state regardless of upstream signaling. To test this hypothesis directly, we artificially forced GBP transcription using CRISPR/Cas9 Synergistic Activation Mediator (CRISPRa-SAM) (Fig 2A), a method previously used for activation of a variety of genes in different cell types (Konermann *et al*, 2014; Chavez *et al*, 2016). In this way, we bypass the need for IFNγ and can determine the role of transcription in gene priming. We successfully established conditions for the CRISPRa-SAM activation of GBP1. A combination of 3 gRNAs targeting the GBP1 promotor, but not gRNAs for the unrelated ASCL1 control gene, is sufficient for GBP1 activation, as validated by RT–qPCR (Fig 2B).

Titration of the concentration and duration of IFNγ exposure revealed that a 24 h treatment of cells with only 1 ng/ml of IFNγ is sufficient to activate GBP1 (Figs 2B and EV1A and B) and induce a primed state that is heritable for at least 48 h (Fig EV1A and C). This level of IFNγ-mediated GBP1 mRNA is comparable to the level of induction by CRISPRa-SAM (Fig 2B). Interestingly, when combining IFNγ with CRISPRa-SAM, we observe further activation of GBP1 (Fig 2B). To determine whether GBP1 expression *per se* is sufficient for priming we transfected HeLa cells with CRISPRa-SAM, targeted by either gRNAs specific for GBP1 or a control gene (ASCL1). We allowed CRISPRa-SAM-driven GBP1 expression to build up for 48 h and then allowed cells to dilute out, prior to induction with IFNγ as outlined in Fig 2C. Our results demonstrate that IFNγ induction of GBP1 is not primed by prior CRISPRa-SAM activation despite similar transcriptional output to IFNγ priming (Fig 2B). Additionally, we combined CRISPRa-SAM activation with IFNγ to prime cells (Fig 2D). Under these conditions, IFNγ primes GBP1 for enhanced re-expression irrespective of any prior CRISPRa-SAM activation. This demonstrates that despite enhanced expression during priming (Fig 2B) CRISPRa has no impact on the degree of priming, consistent with our finding that CRISPRa alone cannot prime GBP1. Combined, unexpectedly, our results indicate that the mere recruitment of Pol II and the activation of the general transcription machinery at GBP1 is not sufficient to induce a prime state and that IFNγ signaling and its downstream transcription factors are necessary to initiate priming.

Increased promoter accessibility of GBP4 and GBP5 in primed cells

As mere transcription does not appear to be the initiator of priming, we reasoned that IFNγ-specific transcription factors upstream of transcription initiation may be required for inducing long-term memory. We started out by determining promoter accessibility as an indirect readout of the degree of transcription factor binding and target gene activation during induction, memory, and reinduction. We performed ATAC-seq in naïve and primed HeLa cells after inducing with IFNγ for 0, 1, and 3 h (Fig EV2A). We find that the promoters of GBP5, and the adjacent GBP4 gene are selectively accessed during IFNγ activation (Fig EV2B). Interestingly, the GBP1 promoter is already in an accessible state in naïve cells and is not significantly opened further by IFNγ activation (Fig EV2B). Interestingly, in primed cells, the GBP5 promoter and, to a lesser extent, GBP4 show accelerated opening during reinduction, particularly after 1 h of IFNγ, but not at IFNγ target genes that do not show priming such as IRF1 and TAP1 (Fig EV2B; Siwek *et al*, 2020).

In agreement with our previous report (Siwek *et al*, 2020) there is no indication of maintenance of an IFNγ-opened nearby enhancer site in primed cells, indicating that latent enhancers are not driving the accelerated reactivation of the GBP genes (Fig EV2C).

STAT1 and IRF1 are required for GBP5 expression

The increased ATAC signals at GBP promoters upon IFNγ induction suggest specific transcription factors target these promotors that may play a role in transcriptional memory. To explore this further,

© 2023 The Authors

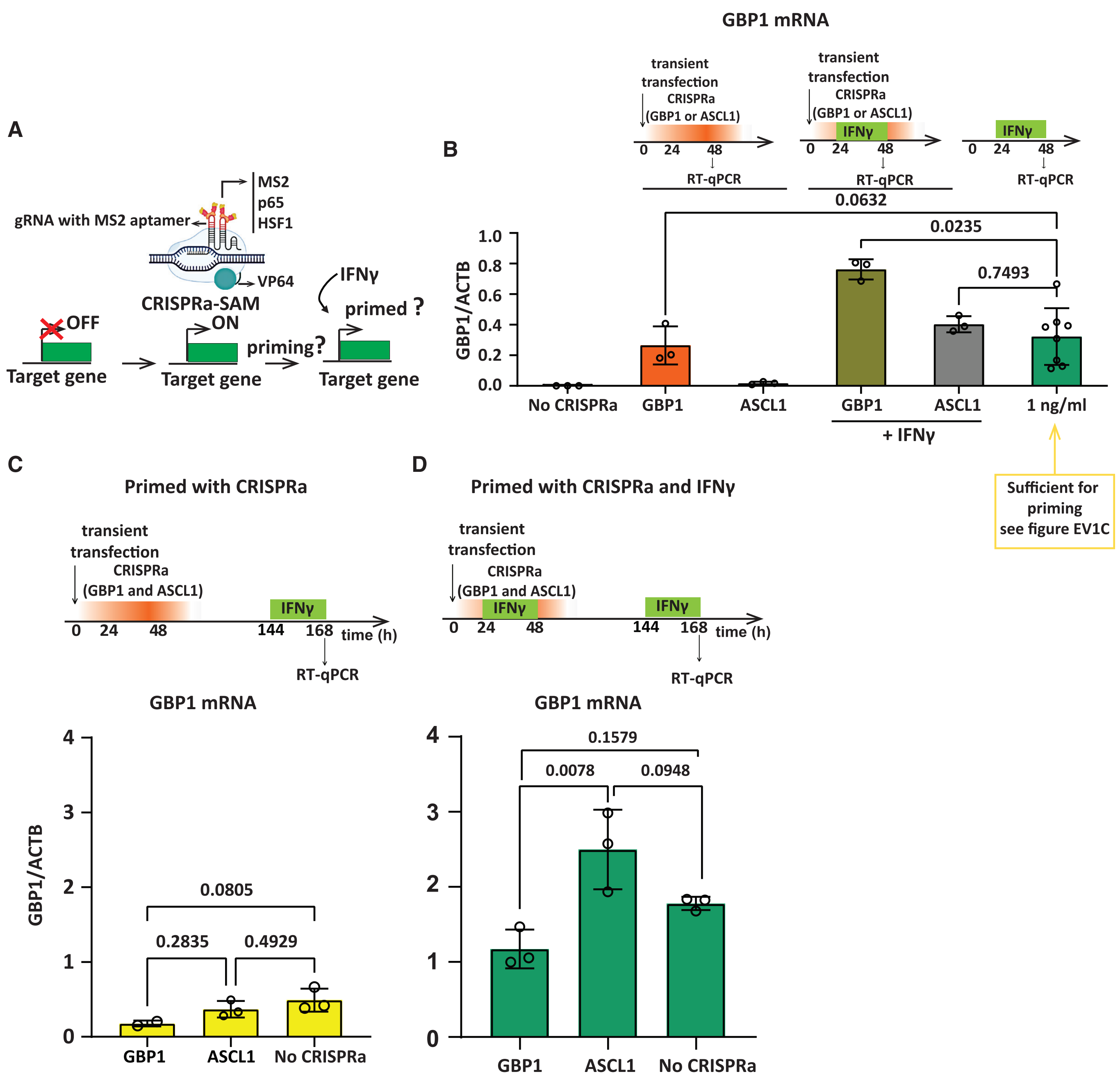

Figure 2. Transcription of a memory gene is not sufficient to induce a primed state.

A Experimental outline; targeting of CRISPR/Cas9 Synergistic Activation Mediator (CRISPRa-SAM), results in forced gene activation, followed by IFNγ to determine the priming state.

B Top: Experimental outline of CRISPRa targeting of GBP1 and ASCL1 (control) by transient transfection with plasmids containing dCAS9-SAM. Bottom: HeLa cells were transfected with dCas9-SAM technology with gRNA for indicated genes, either alone or in combination with a 24 h exposure to IFNγ. RNA was isolated, and GBP1 mRNA expression was measured by RT–qPCR after 48 h of transfection and normalized to ACTB expression. Statistical significance was determined using two-way ANOVA. Data are shown as mean. Error bars, SD; $n = 3$ biological replicates.

C Top: Experimental outline of CRISPRa targeting of GBP1 and ASCL1 (control) by transient transfection with plasmids containing dCAS9-SAM, followed by induction with IFNγ. Bottom: RNA was isolated, GBP1 mRNA level was determined as indicted in experimental outline at top, by RT–qPCR and normalized to ACTB mRNA level. Statistical significance was determined using Ordinary one-way ANOVA. Data are shown as mean. Error bars, SD; $n = 3$ biological replicates.

D Experiment as in (C) but with the addition of IFNγ during priming. Statistical significance was determined using Ordinary one-way ANOVA. Data are shown as mean. Error bars, SD; $n = 3$ biological replicates.

Source data are available online for this figure.

© 2023 The Authors

we first examined the role of transcription factors as effectors of this pathway. The STAT and IRF family of proteins are the main transcription factors responding to interferons (Mogensen, 2018). There are seven members in the STAT family (STAT1, 2, 3, 4, 5A, 5B, and 6) and nine in the IRF family (IRF1-9), which target genes in response to different cytokines (Delgoffe & Vignali, 2013; Antonczyk *et al*, 2019). STAT1 is well-established as the key transcription factor in IFNγ signaling (Antonczyk *et al*, 2019). Upon stimulation, STAT1 is activated by the IFNγ receptor-bound JAK kinase. Phosphorylation results in homodimerization or heterodimerization with other STATs, leading to translocation into the nucleus and target gene activation (Rawlings *et al*, 2004). Genes encoding IRF transcription factors are activated by STAT1 that then cooperate with it to further induce downstream interferon-target genes (Schroder *et al*, 2004). One possible way of achieving a primed state is for a specific transcription factor to respond to IFNγ stimulation in a feed-forward fashion. In such a scenario, IFNγ stimulation results not only in transcription factor activation but also in its continued expression, even after the removal of the cytokine. To determine whether any of the STATs or IRFs behave like this, we mined our RNA-seq dataset (Siwek *et al*, 2020) to determine their expression after induction, when primed and in the reinduction state (Fig 3A). As expected, several STAT and IRF members were strongly induced by IFNγ but following washout, all returned to baseline levels (Figs 3A and EV3D). Thus, a model in which the key drivers of IFNγ-mediated gene expression engage in self-propagating expression is unlikely. However, while the mRNA levels of these genes return to baseline, they may nevertheless be required to establish and/or maintain the primed state upon IFNγ exposure. To dissect the putative role of STAT and IRF proteins in priming in more detail in the HeLa model, we generated CRISPR/Cas9 knockout cell lines of a representative set of transcription factors. These include those most strongly activated by IFNγ; STAT1, STAT2, STAT3, and IRF1, as well as STAT5B and IRF9 (Fig EV3A). Consistent with earlier reports, GBP5 induction is lost in STAT1 and IRF1 knockout cells (Fig 3B; Ramsauer *et al*, 2007), while STAT2, STAT5B, and IRF9 are dispensable both for induction (Fig EV3B), as well as priming (Fig EV3C). Interestingly, STAT3 depletion had the opposite effect, leading to an increase in GBP5 induction (Fig EV3B). From this analysis, we conclude that both STAT1 and IRF1 are required for GBP5 induction. Next, we asked whether these two key transcription factors have any role in establishing or maintaining GBP5 priming.

STAT1 and IRF1 enrichment within the GBP cluster is accelerated during early reinduction

The enhanced promoter accessibility of primed genes upon IFNγ reinduction (Fig EV2B) may be driven by a different rate of promoter binding by the essential transcription factors for GBP5 induction, STAT1, and IRF1. To assess this, HeLa cells were induced with IFNγ for 0, 1, and 3 h, both in naïve and primed cells, followed by CUT&RUN (Cleavage Under Targets and Release Using Nuclease) (Meers *et al*, 2019b) for STAT1 and IRF1 (Fig 3C). These results show that STAT1 and IRF1 target the GBP gene promoters within the first 3 h of IFNγ induction. Interestingly, both bind faster upon reinduction of primed genes GBP5 and GBP4, GBP1P1, as well as an element distal to GBP5 named E1 (Fig 3D and E, Appendix Fig S1A). By contrast, both STAT1 and IRF1 target the GBP1 promoter rapidly within an hour of IFNγ that is not further enhanced in primed cells (Appendix Fig S1A), consistent with the enhanced chromatin accessibility (Fig EV2B). Moreover, unbiased genome-wide analysis in primed vs naïve cells at the promoter of genes (−3 kb to +3 kb relative to TSS) revealed that GBP5, GBP1, and GPB4 were among the top loci for accelerated STAT1 and IRF1 recruitment at least at the early time point (Fig 3F).

To explore non-promotor binding sites, we expanded our search to whole genes with 3 kb upstream and downstream of the gene body. Interestingly, there are only 12 sites, genome-wide that show accelerated binding of both STAT1 and IRF1 (Fig 3G, Appendix Fig S1B), and among those, seven map within the GBP cluster (numbered 1–7 in Appendix Fig S1B). Moreover, visual inspection of the GBP cluster identified one additional site with faster STAT1 and IRF1 recruitment in primed cells, which were outside of our defined search range (±3 kb of gene body), and mapped distal to the GBP5 promoter (Fig 4A, Element 2). Together, these results suggest that faster recruitment of both STAT1 and IRF1 upon reinduction is a feature strongly associated with the GBP cluster (Fig 4B), both at primed gene promoters, as well as 2 elements 16 and 37 kb upstream of the GBP5 promoter. Next, we aimed to determine whether the accelerated binding of both STAT1 and IRF1 in primed cells is the consequence of changes in upstream signaling.

GBP5 priming is not dependent on the regulation of IFNγ-induced STAT1 expression

One possible explanation for GBP5 priming and enhanced promoter binding by STAT1 is STAT1 priming itself. Our RNA-seq analysis shows that STAT1 is strongly induced by IFNγ (Siwek *et al*, 2020; Fig 3A, as previously reported Cheon & Stark, 2009). Although this bulk RNA-seq analysis does not show a significant enhancement of the expression of STAT1 in primed cells, our single-cell RNA sequencing data from the same study (Siwek *et al*, 2020), did show a modest degree of priming of STAT1 mRNAs (Fig 5A). To determine whether priming of STAT1 expression is relevant for GBP5 priming, we expressed STAT1 from a constitutive promoter in cells in which endogenous STAT1 was deleted (Fig 5B). Effectively, in these cells, STAT1 expression is uncoupled from IFNγ induction and maintained at a level similar to that of induced cells (Fig 5C). Interestingly, while STAT1 is no longer IFNγ-regulated we find that GBP5 expression remains strictly IFNγ-dependent and priming still occurs (Fig 5D). It should be noted that endogenous STAT1 is expressed as different isoforms (Schindler *et al*, 1992; Zakharova *et al*, 2003). We opted to clone and express the main isoform that, although having a lower apparent molecular weight, it is functional in supporting IFNγ-indued expression of GBP genes. Thus, while overall GBP5 expression in these cells is consistently lower (Fig 5D, Appendix Fig S2A), it has no bearing on the degree of memory (Fig 5D, Appendix Fig S2B), indicating that STAT1 expression is not rate limiting in priming.

Upstream STAT1 phospho-dynamics and import, are not altered in primed cells

Upon IFNγ stimulation, STAT1 is activated by phosphorylation at Tyr701 via JAK kinase (Darnell *et al*, 1994). One possible mechanism of retention of the primed state is that after IFNγ induction,

© 2023 The Authors

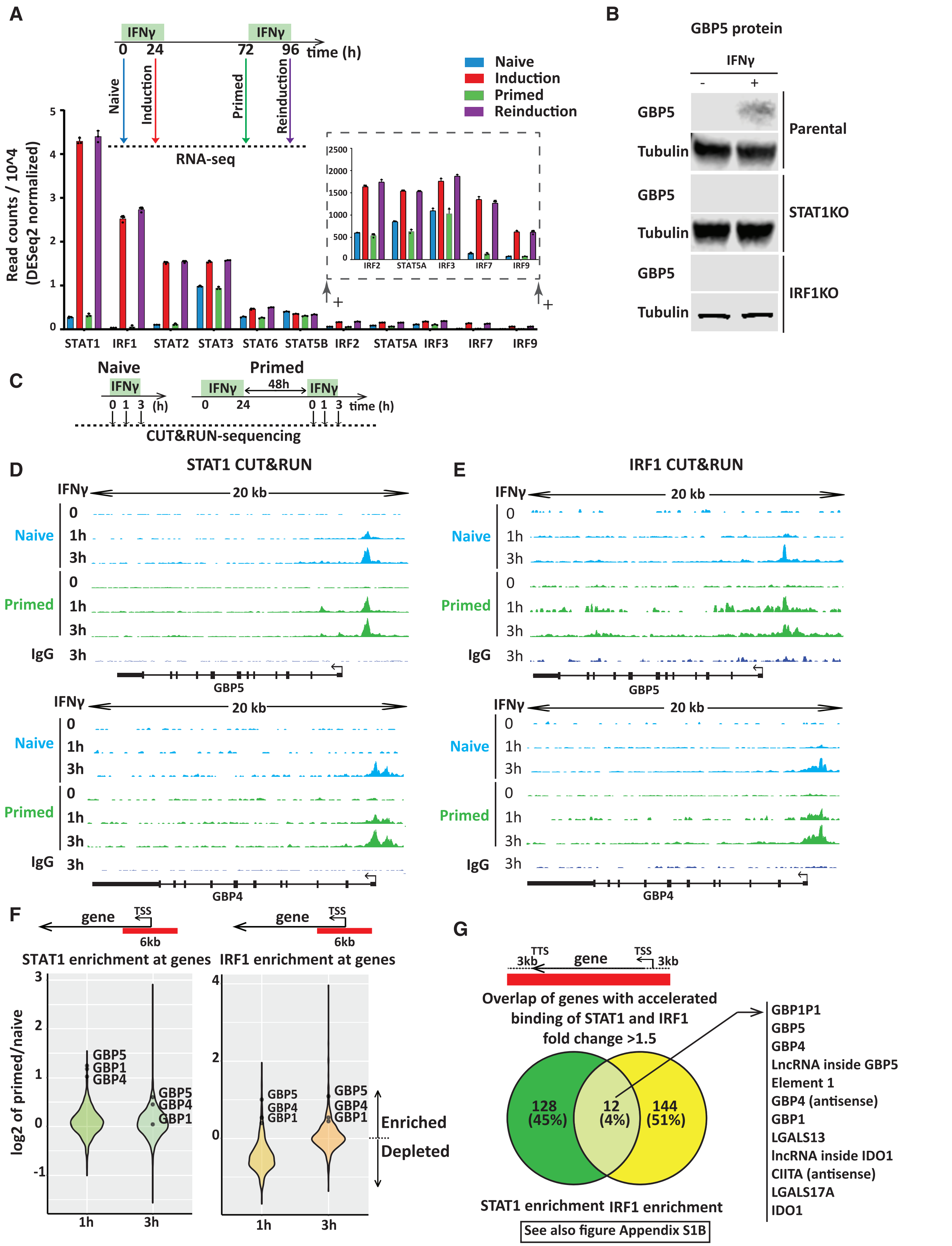

Figure 3.

© 2023 The Authors

Figure 3. STAT1 and IRF1 are essential for GBP5 expression and show enhanced recruitment to target gene promoters during early reinduction.

A Top: Experimental outline of IFNγ induction and reinduction regime. Bottom: mRNA levels of STAT and IRF family members at indicated time points based on the experiment outlined in the top (obtained from dataset reported by Siwek *et al*, 2020). Transcription factors are ordered by their expression level. Data are shown as mean. Error bars, SD; $n = 3$ biological replicates.

B Stable CRISPR knockouts were generated for indicated genes in HeLa cells. Knockout (KO) cells or their parental controls (WT) were induced with IFNγ for 24 h or left untreated and prepared for SDS–PAGE and immunoblotting. Blots incubated with GBP5 antibody assess gene expression. α-Tubulin (Tubulin) was used as a loading control. Note that GBP5 and Tubulin blot of parental cells is as in Fig 1C.

C Scheme describing STAT1 and IRF1 transcription factor enrichment by CUT&RUN.

D–G HeLa cells were primed with IFNγ for 24 h, followed by IFNγ washout. After 48 h, naïve and primed cells were induced by IFNγ for 1 and 3 h. Cells were harvested at indicated time points and processed for CUT&RUN. Representation of processed data of CUT&RUN for STAT1 (D) and IRF1 (E) occupancy at GBP5 and GBP4 genes. Sequenced reads were mapped to the human genome (hg38), and coverage data are displayed as reads per million (RPM) at equal scaling. (F) Violin plot showing log2 fold change of STAT1 and IRF1 enrichment upon treatment of primed relative to naïve cells at the promoter (−3 kb to +3 kb relative to TSS) of all annotated genes as measured by CUT&RUN. Data (primed/naïve) is plotted for 1 and 3 h IFNγ treatment. (G) Venn diagram displaying overlap of STAT1 and IRF1 enrichment for genes (−3 kb of TSS and +3 kb of TTS) that have more than 1.5-fold change differences between primed and naïve upon 1 h of IFNγ treatment.

Source data are available online for this figure.

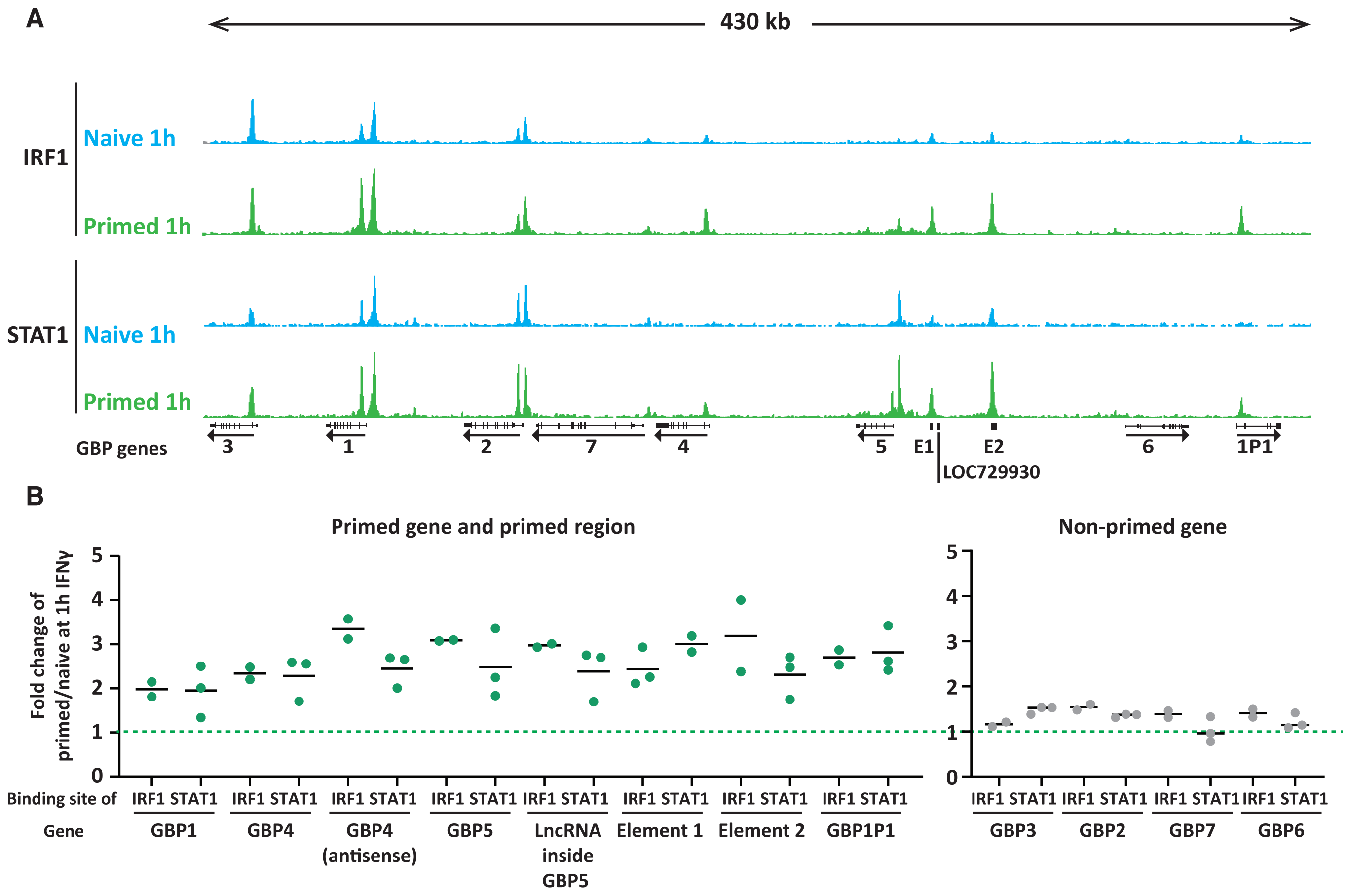

Figure 4. Accelerated recruitment of STAT1 and IRF1 across the primed GBP cluster.

A Tracks of processed CUT&RUN data for STAT1 and IRF1 occupancy across the GBP cluster following 1 h of our IFNγ induction in naïve and primed cells. Results of sequenced reads were mapped to the human genome (hg38), and coverage data are displayed as reads per million (RPM) at equal scaling. GBP gene positions are indicated.

B Quantification of STAT1 and IRF1 enrichment in primed cells relative to naïve cells, 1 h after IFNγ induction. Fold change is shown for the 7 loci within the GBP cluster listed in Fig 3G and Appendix Fig S1B, as well as an additional site (E2) distal to GBP5 (green data points). Enrichment ratios for nonprimed genes (gray) are shown for comparison. Each dot represents one biological replicate. The line shows the mean of the data.

Source data are available online for this figure.

JAK proteins remain active at a low level or stay in a poised state leading to faster activation upon reinduction. To determine the impact of JAK/STAT signaling on GBP gene induction and the maintenance of transcriptional memory, we inhibited JAK kinase using a specific inhibitor (CP-690550, here abbreviated as JAKi). Upon JAK inhibition during IFNγ induction GBP5 expression was

© 2023 The Authors

lost (Fig 5E), consistent with prior reports (Ramana *et al*, 2000; Migita *et al*, 2011). Having established conditions to effectively inhibit JAK, we then treated cells with JAKi immediately following the initial IFNγ induction and kept JAK inhibited during the memory window until just before the second induction (Fig 5F). These results indicate that inhibition of JAK after priming does not significantly affect transcriptional memory indicating that JAK/STAT signaling is required for GBP induction but is dispensable for maintaining memory. While JAK-mediated signaling is not required after priming, it may be hyperactive upon IFNγ exposure, leading to accelerated STAT1 phosphorylation and enrichment on GBP promoters. To determine this, we measured STAT1 Y701 phosphorylation in naïve and primed cells after IFNγ induction at different time points. We find that STAT1 phosphorylation is fast both in naïve and primed cells with a possible minor increase in rate at early time points (Fig 5G). To further test whether priming has a differential impact on STAT1 activity, we measured STAT1 import into the nucleus, which is the downstream consequence of STAT1 activation (Ramana *et al*, 2002). For this, we employed a cell line constitutively expressing a GFP-tagged STAT1 transgene (described in detail below) and measured STAT1-EGFP levels by live cell imaging (Fig 5H). Treatment of cells with IFNγ leads to STAT1 accumulation

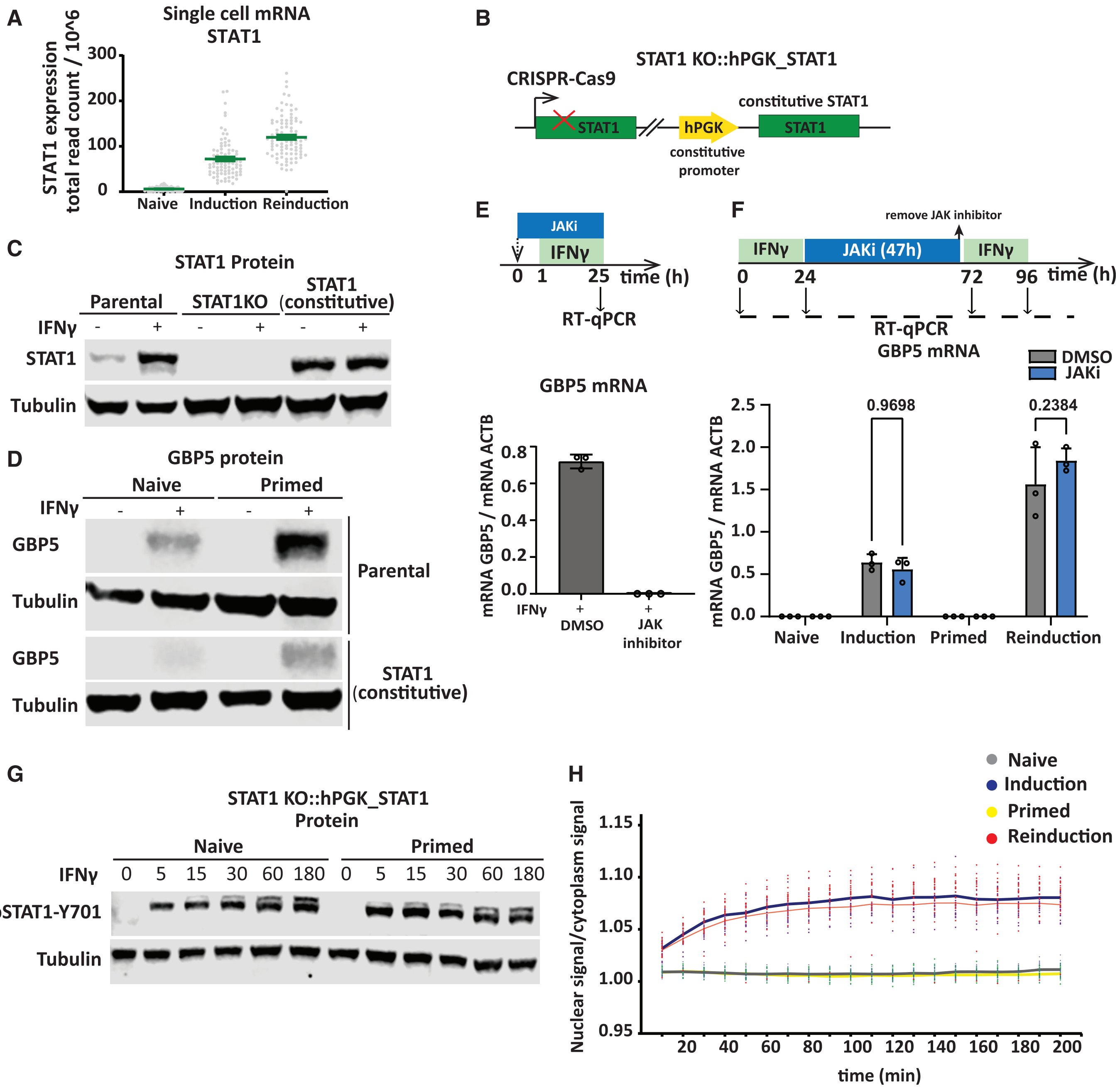

Figure 5.

 © 2023 The Authors

Figure 5. STAT1 expression, activation and import are not rate limiting for priming.

A Single-cell RNA-seq from HeLa cells for STAT1 from data described in (Siwek *et al*, 2020). Each dot represents STAT1 expression in one cell in naïve (n = 91), induction (n = 90) and reinduction (n = 92). Error bars, SEM.

B Scheme outlining genotype of STAT1 knockout cell line, rescued with constitutive expression of STAT1 from a lentiviral vector.

C Blot probing for STAT1 before and after IFNγ induction in STAT1 knockout (STAT1KO), STAT1 rescued (STAT1, constitutive), and parental control (WT), to confirm knockout and rescue status. α-Tubulin (Tubulin) was used as a loading control.

D STAT1 rescue cells and their parental control were subjected to IFNγ induction and reinduction regime as outlined in Fig 1A. Cell extracts were processed for western blotting and probed for GBP5 expression before and after induction and reinduction as indicated in Fig 1A. α-Tubulin (Tubulin) was used as a loading control.

E Top: Scheme describing HeLa cells treated with JAK inhibitor CP-690550 (JAKi, 10 μM) or DMSO vehicle control for 25 h together with IFNγ induction for 24 h. Bottom: RT–qPCR analysis of GBP5 expression in induced cells treated with JAK inhibitor CP-690550 (JAKi, 10 μM) or DMSO vehicle control. Error bars, SD; n = 3 biological replicates.

F Top: Schematic overview of JAK inhibitor treatment during memory window experiment; HeLa cells primed with IFNγ for 24 h, were treated with JAK inhibitor CP-690550 (JAKi, 10 μM) or DMSO vehicle control for 47 h, followed by drug washout. After 1 h, cells were reinduced with IFNγ for 24 h. Bottom: GBP5 mRNA level after induction and reinduction in the context of JAKi and DMSO was determined by RT–qPCR and normalized to ACTB mRNA level. Statistical significance was determined using two-way ANOVA. Error bars, SD; n = 3 biological replicates.

G Cells constitutively expressing STAT1 (as in B) were primed with IFNγ, followed by IFNγ washout. After 48 h, naïve and primed cells were induced by IFNγ for different time points (5, 15, 30, 60, 180 min). Cell extracts were prepared at indicated time points and processed for western blotting. Immunoblot of pSTAT1-Y701, and α-Tubulin (Tubulin) as a loading control.

H STAT1-EGFP-dTAG cells were primed with IFNγ, followed by IFNγ washout. After 48 h, naïve and primed cells were induced with IFNγ and prepared for live cell imaging. Images were acquired 10 min after IFNγ addition at 10-min intervals. The ratio of STAT1-EGFP in the nucleus over cytoplasm was quantified. Each dot represents the ratio of EGFP fluorescence intensity in one cell. The line shows the mean of data.

Source data are available online for this figure.

in the nucleus; however, STAT1 is not retained in primed cells, and nuclear accumulation during induction and reinduction is indistinguishable. We also imaged STAT1 protein by immunofluorescence to quantify nuclear and cytoplasmic pools (Fig EV4A). Consistent with the live cell data, the rate of STAT1 import into the nucleus is not detectably different between the 1st and 2nd induction, and STAT1 is not maintained in the nucleus after the removal of IFNγ (Fig EV4B). These results indicate that the rates of JAK-mediated STAT1 activation and import are not significantly altered in primed cells compared with naïve cells.

STAT1 phosphorylation at Ser727 is maintained after removal of IFNγ

In addition to JAK-mediated phosphorylation at Tyr701, STAT1 is also phosphorylated at Ser727, which is required for maximal STAT1 activation and transcriptional activity (Wen *et al*, 1995). A previous study has shown that CDK8, a chromatin-associated kinase known to target Ser727 (Bancerek *et al*, 2013), remains active in primed cells (D'Urso *et al*, 2016). We determined the degree of both Tyr701 and Ser727 phosphorylation before and after IFNγ induction in naïve and primed cells. Interestingly, while Tyr701 phosphorylation is rapidly lost after the removal of IFNγ, Ser727 phosphorylation is maintained for up to 7 days after priming (Figs 6A and EV5A). To test the necessity of STAT1 phosphorylation at Ser727 in transcriptional memory, we rescued the STAT1 knockout cell line (Fig 3B) with a transgene expressing STAT1 in which Ser727 was mutated to alanine (S727A) (Fig EV5B). Immunoblotting with phospho-specific antibodies for S727P confirmed loss of phosphorylation (Fig EV5C). While STAT^{S727A} expression levels were lower compared with IFNγ-induced levels, this mutant form retained partial functionality as revealed by its ability to support IFNγ-mediated IRF1 induction, which is STAT1-dependent (Fig EV5C). Nevertheless, this level of STAT1 activity was insufficient to detectably induce GBP5 expression as our readout for transcriptional memory (Fig EV5D and E). This indicates that STAT1 phosphorylation at Ser727 plays a critical role in GBP5 expression, which precluded us from further exploring this mutant. We then asked whether a constitutively phosphorylated-like variant of STAT1 impacts on expression and memory. We generated cells in which we expressed STAT1-bearing phosphomimetic negative charge mutations at the S727 (S272E and S727D) in a STAT1 knockout background (Fig 6B). Unlike the nonphosphorylatable mutant, these phosphomimetic mutants are still able to robustly support GBP expression (Fig 6C) indicating that S727 phosphorylation is required for expression. However, the addition of charged residues does not lead to enhanced memory. Instead, the degree of priming is reduced as judged by the ratio of expression between reinduction and priming (Fig 6C). This suggests that the differential phosphorylation of this residue between naïve cells (no S727 phosphorylation) and primed cells (memorized S272 phosphorylation) is functionally relevant for memory and may depend on the dynamic of control of the phosphorylated state.

STAT1 is required during priming to establish GBP5 transcriptional memory but is dispensable during memory of the primed state

Thus far we showed that STAT1 binds to GBP target gene promotors more rapidly in primed cells (Fig 3D) and that STAT1 activation leads to multiday retention of Ser727 phosphorylation with functional consequences for GBP expression and memory (Fig 6). To circumvent this limitation and directly test the hypothesis that STAT1 itself carries the memory of prior IFNγ induction, we constructed a STAT1 allele tagged with a destruction tag (dTAG) composed of a modified FKB12 protein, that can be selectively degraded with the small molecule dTAG13 (Nabet *et al*, 2018). This allows us to determine whether STAT1 has a role in the maintenance of the primed state without affecting its essential role in GBP gene expression upon IFNγ exposure. We expressed STAT1 C-terminally tagged with EGFP-dTAG in HeLa STAT1 knockout cells (Fig 6D). To characterize the functionality of the degron we tested STAT1 dynamics in the context of our IFNγ induction and reinduction regime. Following IFNγ priming, we can effectively remove the vast majority of STAT1

© 2023 The Authors

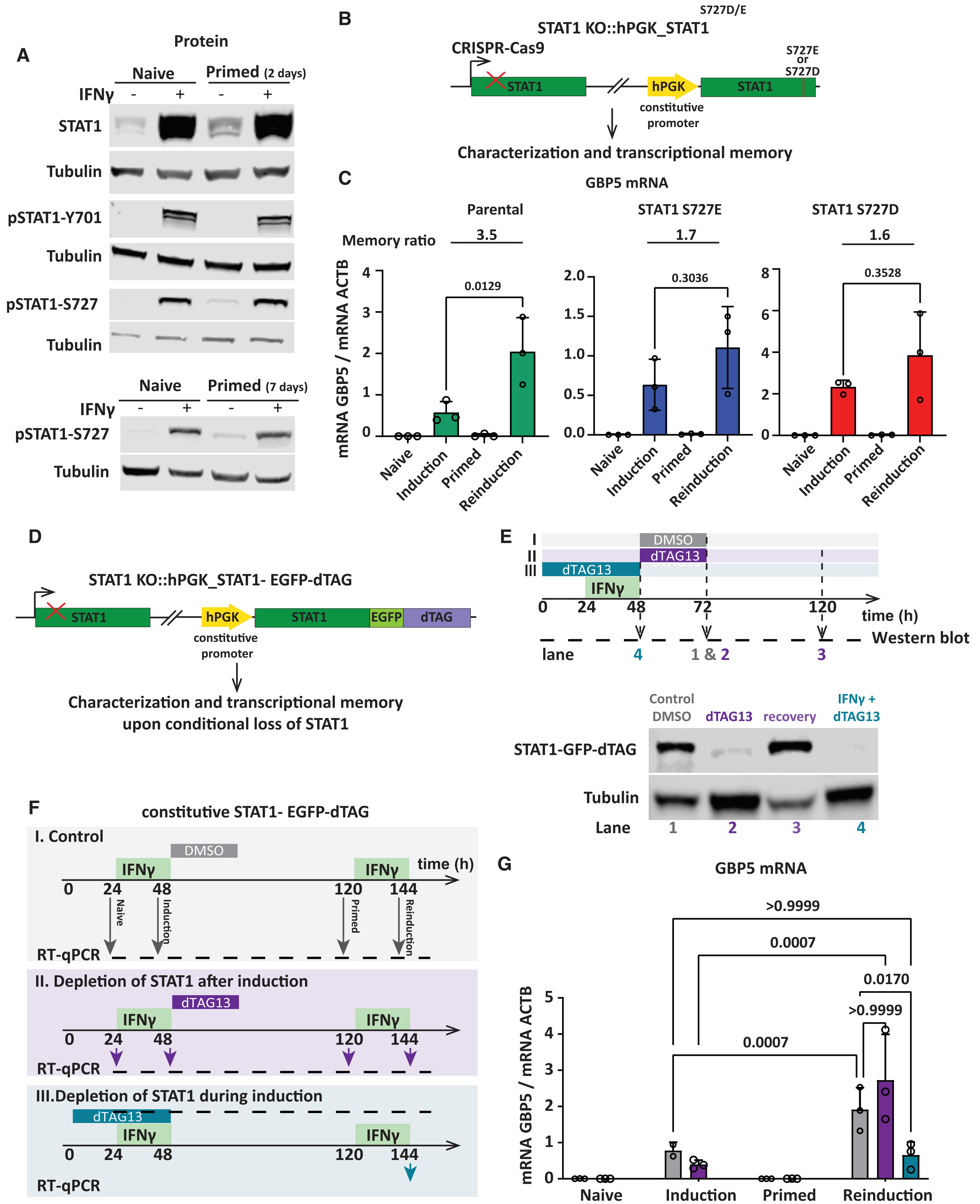

Figure 6.

© 2023 The Authors

Figure 6. STAT1 phosphorylation at Ser727 is inherited after removal of IFNγ but not required for priming.

A HeLa cells were subjected to IFNγ induction and reinduction regime as outlined in Fig 1A with 2 and 7 days recovery time (primed state) after IFNγ washout. Cell extracts were prepared at indicated time points, processed for western blotting, and probed for STAT1, pSTAT1-Y701, and pSTAT1-S727. α-Tubulin (Tubulin) as a loading control.

B Schematic overview of expression of a STAT1 variant with S727E or S727D mutations from a constitutive promoter.

C (H) STAT1KO::STAT1-S727E and STAT1KO::STAT1-S727D expressing cells or their parental controls (WT) were subjected to IFNγ induction and reinduction regime as outlined in Fig 1A, RNA was isolated and GBP5 mRNA level was determined by RT–qPCR and normalized to ACTB mRNA level. Statistical significance was determined using Ordinary one-way ANOVA. Data are shown as mean (error bars, SD; $n = 3$ biological replicates).

D Schematic overview of STAT1 KO cells rescued with STAT1-EGFP-dTAG (FKB12F36V) from a constitutive promoter.

E Characterization of dTAG-induced destruction and recovery. STAT1-EGFP-dTAG expressing cells were induced with IFNγ for 24 h, then treated with dTAG13 (100 nM) for DMSO vehicle control (I) or STAT1 depletion (II) for 24 h. To determine recovery of STAT1 expression, cells were washed three times with medium and harvested 48 h after IFNγ washout (120 h time point, lane 4). In parallel, (III) cells were treated with dTAG13 (100 nM) for 48 h during 24 h induction with IFNγ. Immunoblot of STAT1-GFP-dTAG at indicated treatments confirms STAT1 depletion and recovery, respectively. α-Tubulin (Tubulin) was used as a loading control.

F Experimental outline of dTAG13 depletion and recovery experiment for STAT1 during and after induction [analogous to C but separately outlined for control (I), depletion after induction (II), and depletion during induction (III)].

G RNA was isolated and GBP5 mRNA level as indicated in (F) was determined by RT–qPCR, normalized to ACTB mRNA level. Statistical significance was determined using two-way ANOVA. Data are shown as mean (error bars, SD; $n = 3$ biological replicates).

Source data are available online for this figure.

within 24 h of dTAG13 addition, while DMSO controls retain STAT1 levels (Fig 6E). By 48 h post removal of dTAG13, STAT1 levels are fully recovered, prior to IFNγ reinduction. Having established conditions in which we can selectively remove STAT1 only after priming and re-express prior to reinduction, we determined the effect of STAT1 removal on transcriptional memory (Fig 6F). We find that STAT1 depletion immediately after priming does not impair GBP5 memory and allows for enhanced GBP5 expression upon IFNγ reinduction, similar to controls (Fig 6G). These results demonstrate that STAT1 and its associated posttranslational modifications are not necessary for the maintenance of GBP5 transcriptional memory. To further explore the role of STAT1, we aimed to determine the requirement of STAT1 during priming. While STAT1 is necessary for GBP5 induction, whether it plays a role in priming has not been determined. Possibly, IFNγ signaling leads to priming via factors other than STAT1. To test this directly, we depleted STAT1 with dTAG13 prior to, and during IFNγ priming (Fig 6F). We then let STAT1 levels recover before IFNγ reinduction. Strikingly, under these conditions, GBP5 expression is not enhanced after the 2nd IFNγ induction. Despite STAT1 presence during reinduction, the cells behave as if naïve despite prior exposure to IFNγ (Fig 6G). These results demonstrate that STAT1 is necessary during cell priming, not only to induce GBP expression but also to establish transcriptional memory.

Discussion

In this report, we dissected the role of STAT1 in the establishment and maintenance of IFNγ-induced transcriptional memory. We focused on GBP5, GBP4, and GBP1 as target genes that show strong priming upon IFNγ induction, as previously described (Gialitakis *et al*, 2010; Siwek *et al*, 2020) to understand transcriptional memory (Fig 1A). Prior work showed that RNA polymerase II (Pol II) is retained at promoters following IFNγ priming (Light *et al*, 2013) or is recruited more rapidly following reinduction (Kamada *et al*, 2018). An increase in both chromatin accessibility (Fig EV2) and Pol II recruitment (Kamada *et al*, 2018) can be due to the alteration of local chromatin structure induced by transcription, regardless of upstream signaling. However, we find that transcription *per se*, induced using CRISPRa-SAM-mediated gene activation is not sufficient to provoke the primed state nor does it add to the ability of IFNγ to prime cells (Fig 2). Artificial CRISPRa-mediated gene activation is known to bring RNA Pol II, the general transcription machinery, and increases the acetylation levels in nucleosomes (Giménez *et al*, 2016). Nevertheless, the recruitment of these factors is not sufficient to prime the GBP target gene, implying a specific IFNγ-mediated factor is required. Furthermore, these results suggest that while RNA polymerase II has been detected on promoters of primed HLA genes (Light *et al*, 2013), it may itself not be the primary driver of transcriptional memory. In line with this, recent elegant experiments in yeast have shown that in the case of *INO1* transcriptional memory, RNA polymerase II is poised but not required for memory (Sump *et al*, 2022).

We showed both STAT1 and IRF1 are required for GBP5 induction, and both display an accelerated recruitment to the promoters of primed genes during reinduction (Fig 3). Intriguingly, the set of loci that show enhanced recruitment of both STAT1 and IRF1 is largely restricted to the GBP cluster (Figs 3G and 4). We excluded priming of STAT1 expression or the presence of STAT1 protein to be involved in the maintenance of memory (Figs 5D and 6E). Combined, these results lead us to propose that the primed state, induced by IFNγ exposure, does not involve upstream JAK–STAT signaling nor is a consequence of mere target gene expression. Instead, memory appears to be restricted to a state induced by STAT1 that may include changes in local chromatin structure, specifically at memorized GBP genes that facilitate accelerated recruitment of STAT1 and IRF1. Interestingly, we detected Ser727 phosphorylation of STAT1 in primed cells (Figs 6A and EV5A) for up to 7 days of memory. While our STAT1 depletion experiments demonstrate that this phosphorylation is not the carrier of the primed state (Fig 6G), it is possible that the underlying chromatin maintains the capacity to rapidly activate STAT1 upon rebinding to promoters. Indeed, our mutational analysis of Ser727 suggests that phosphorylation of this residue contributes to robust priming (Fig 6C). In this vein, it is noteworthy that the putative kinase for STAT1 Ser727, CDK8 is maintained on target gene chromatin in IFNγ-primed cells (D'Urso *et al*, 2016) and that CDK8 occupancy correlates with STAT1 S727 phosphorylation (Bancerek *et al*, 2013). Other possible changes in chromatin structure and composition may include SMARCA4 (BRG1), an SWI/SNF-related remodeling

complex (Sif *et al*, 2001) that can be recruited by STAT1 to GBP genes (Ni *et al*, 2005).

In sum, our work defines that IFNγ priming results in STAT1-dependent changes of primed genes, excluding effects in upstream signaling or downstream transcription activation. This focuses future efforts on identifying what factors cause local chromatin changes that enhance target gene expression upon IFNγ re-exposure.

Materials and Methods

Reagents and Tools table

Reagent/Resource	Reference or source	Identifier or catalog number
Experimental models: cell lines		
Human: HeLa WT	ATCC	Cat#CCL-2; RRID: CVCL_0030
Human: HeLa Kyoto	https://web.expasy.org/cellosaurus/CVCL_1922	RRID: CVCL_1922
Human: HEK293	ATCC	CRL-1573; RRID: CVCL_0045
Human: HeLa STAT1 knockout polyclonal	This study	N/A
HeLa STAT1 knockout clonal	This study	N/A
HeLa constitutive STAT1 polyclonal	This study	N/A
HeLa constitutive STAT1^{S727A} polyclonal	This study	N/A
HeLa constitutive STAT1-GFP-FKB12^{F36V} (dTAG13) polyclonal population	This study	N/A
HeLa STAT2 knockout polyclonal	This study	N/A
HeLa STAT3 knockout polyclonal	This study	N/A
HeLa STAT5B knockout polyclonal	This study	N/A
HeLa IRF1 knockout polyclonal	This study	N/A
HeLa IRF9 knockout polyclonal	This study	N/A
Recombinant DNA		
lentiCRISPR v2	Sanjana *et al*, 2014; Addgene	Addgene #52961
psPAX2	Addgene	Addgene #12260
pMD2.G	Addgene	Addgene #12259
lentisgRNA(MS2)_Puro	Addgene	Addgene #73795
lentiMS2-P65-HSF1_Hygro	Addgene	Addgene #61426
lenti dCas9-VP64_Blast	Addgene	Addgene #61425
pRosetta	Addgene	Addgene ##59700
pRosetta _STAT1	This study	N/A
pRosetta _STAT1^{S727A}	This study	N/A
EGFP-dtag-HA	Jansen Lab	N/A
Antibodies		
GBP5	Cell Signaling Technology	Cat#67798
GBP5	Abcam	Cat#ab96119
EGFP	Merck	Cat#G1544
a-TUB	Merck	Cat#T9026
Anti-rabbit (fluorophore conjugated)	LI-COR	Cat#926-32211
Anti-mouse (fluorophore conjugated)	Rockland	Cat#610-744-124
STAT1	Cell Signaling Technology	Cat#9172
pSTAT1 (701)	Cell Signaling Technology	Cat#9167
pSTAT1 (727)	Cell Signaling Technology	Cat#9177

© 2023 The Authors

Reagents and Tools table (continued)

Reagent/Resource	Reference or source	Identifier or catalog number
STAT2	Santa Cruz Biotechnology	Cat#sc-1668
STAT3	Santa Cruz Biotechnology	Cat#sc-8019
STAT5B	Santa Cruz Biotechnology	Cat#sc-1656
IRF1	Cell Signaling Technology	Cat#8478
IRF9	ThermoFisher Scientific	Cat#702322
Rabbit IgG	Epicypher	Cat#13-0042k
Oligonucleotides and other sequence-based reagents		
CRISPRa gRNAs	This study	Appendix Table S1
CRISPR/Cas9 gRNAs	This study	Appendix Table S1
qPCR primers	This study	Appendix Table S1
GeneArt Strings DNA Fragments (STAT1 cDNA)	ThermoFisher	NA
Bacterial and virus strains		
ER2566	Jansen Lab	N/A
DH5alpha	Jansen Lab	N/A
Chemicals, peptides, and recombinant proteins		
Interferon gamma (IFNγ)	Merck	Cat#SRP3058; CAS: 9008-11-1
Vybrant DyeCycle Ruby Stain	ThermoFisher	Cat#V10309
Tofacitinib citrate	Merk	Cat# PZ0017
dTAG13	Merck	Cat #SML2601-1MG
T4 DNA Polymerase	New England Biolabs	Cat#M0203S
Lipofectamine LTX with Plus Reagent	ThermoFisher	Cat# A12621
Software		
CellProfiler v. 3.1.9	https://cellprofiler.org/	N/A
ImageJ v. 1.52h	https://imagej.nih.gov/ij/	N/A
GraphPad Prism 9.5.1	https://www.graphpad.com/scientific-software/prism/	N/A
RStudio v. 1.3.1093	https://www.rstudio.com/	N/A
Deeptools v. 3.5.0	https://anaconda.org/bioconda/deeptools	N/A
Samtools v. 1.15	https://anaconda.org/bioconda/samtools	N/A
Bowtie2 v. 2.4.5	https://anaconda.org/bioconda/bowtie2	N/A
GPP sgRNA Designer software	https://portals.broadinstitute.org/gpp/	N/A
Biorender	https://biorender.com/	N/A
Other (*Kits, instrumentation, laboratory equipment, lab ware etc*)		
Monarch® RNA Cleanup Kit	New England Biolabs	Cat#T2040L
RNA-to-cDNA Kit	Applied Biosystems	Cat#4387406
CUTANA™CUT&RUN kits	EpiCypher	Cat#14_1048
NEBNext Ultra II DNA Library Prep Kit for Illumina	New England Biolabs	Cat#E7645S
TDE1 Tagment DNA Enzyme	Illumina	Cat#15027865

Methods and Protocols

Human cell lines

i HeLa (female, RRID: CVCL_0030); used in Figs 1, 3, 5, 6 and EV2–EV5.
ii HeLa Kyoto (female, RRID: CVCL_1922); used in Figs 2–4 and EV1, Appendix Fig S1.
iii HeLa STAT1 knockout polyclonal; used in Fig 5.
iv HeLa STAT1 knockout clonal; used in Figs 5 and EV5C.
v HeLa constitutive STAT1 polyclonal; used in Fig 5, Appendix Fig S2.
vi HeLa constitutive STAT1^{S727A} polyclonal; used in Fig EV5.
vii HeLa constitutive STAT1^{S727E} polyclonal; used in Fig 6.
viii HeLa constitutive STAT1^{S727D} polyclonal; used in Fig 6.

© 2023 The Authors

ix HeLa constitutive STAT1-GFP-FKBP12 F36V (dTAG13) polyclonal; used in Figs 5 and 6.
x HeLa STAT2 knockout polyclonal; used in Fig EV3.
xi HeLa STAT3 knockout polyclonal; used in Fig EV3.
xii HeLa STAT5B knockout polyclonal; used in Fig EV3.
xiii HeLa IRF1 knockout polyclonal; used in Figs 3 and EV3.
xiv HeLa IRF9 knockout polyclonal; used in Fig EV3.
xv HEK239T (female, RRID: CVCL_0045); used for virus production.

Cell lines and culture conditions

All cell lines were incubated at 37°C, 5% CO_2, and grown in Dulbecco's Modified Eagle Medium (DMEM) containing high glucose and pyruvate (ThermoFisher, 41966-029) supplemented with 10% NCS (newborn calf serum, ThermoFisher, 16010-159) and 1% Penicillin–Streptomycin (ThermoFisher, 15140-122). For passaging, cells were washed using 1× DPBS (ThermoFisher), detached using TrypLE Express phenol red (ThermoFisher), and resuspended in DMEM. Cells were counted using Countess™ Cell Counting according to the manufacturer's instructions (Thermo Fisher Scientific). Transfection of cells was performed using Lipofectamine LTX (Thermo Fisher Scientific) according to the manufacturer's instructions. Cells were routinely tested for Mycoplasma contamination.

Method details

Reagents

All chemicals, unless otherwise noted, were obtained from ThermoFisher or Merck. Enzymes were obtained from New England Biolabs. The following drugs/dyes were used for this work: IFNγ (final concentration of 50 ng/ml, Merck), dTAG13 (final concentration of 100 nM, Merck), Vybrant DyeCycle Ruby Stain (final concentration of 5 μM, ThermoFisher) and Tofacitinib citrate also known as CP-690550 (JAK inhibitor, concentration of 10 μM, Merck). The following antibodies were used for this work: GBP5 (Cell Signaling Technology, 67798; Abcam, ab96119), STAT1 (Cell Signaling Technology, 9172), Phospho-STAT1 (Ser727) (Cell Signaling Technology, 9177), Phospho-STAT1 (Tyr701) (58D6) (Cell Signaling Technology, 9167), STAT2 (Santa Cruz Biotechnology, sc-1668), STAT3 (Santa Cruz Biotechnology, sc-8019), STAT5B (Santa Cruz Biotechnology, sc-1656), IRF1 (Cell Signaling Technology, 8478), IRF9 (ThermoFisher Scientific, 702322), a-TUB (Merck, T9026), anti-rabbit (fluorophore-conjugated) (LI-COR, 926-32211), anti-mouse (fluorophore-conjugated) (Rockland, 610-744-124), Rabbit IgG (Epicypher, 13-0042k).

DNA constructs and genome engineering

A constitutively expressed STAT1 was constructed in the pRosetta plasmid (Addgene ##59700). First, the EGFP sequence was deleted by PCR from the plasmid backbone using primers (5″-gaagcggag ctactaacttcagcctgctgaagcagg-3″, 5″-ggtggatccccctggggagagaggtcg-3″). The product was blunted by T4 DNA Polymerase (New England Biolabs) and self-ligated with T4 DNA Ligase (New England Biolabs). A synthetized fragment was cloned into this vector carrying the cDNA of the α isoform of STAT1 with a silent PAM site mutation and 25 bp homology arms by the SLIC method (Jeong *et al*, 2012).

To construct the STAT1^{S727A} variant in the pRosetta-STAT1 plasmid, mutations were introduced using the primers 5″-ACAACCTGC TCCCCATGGCTCCTGAGGAGTTTGACG-3″ and 5″-CGTCAAACTCC TCAGGAGCCATGGGGAGCAGGTTGT-3″, replacing Serine 727 in STAT1 to Alanine (The underline nucleotides are the introduced mutations).

To construct the STAT1^{S727E} variant in the pRosetta-STAT1 plasmid, mutations were introduced using the primers 5″-CCCCATGG AACCTGAGGAGTTTGACGAGGTGTCTCG-3″ and 5″-CGTCAAACT CCTCAGGTTCCATGGGGAGCAGGTTGT-3″, replacing Serine 727 in STAT1 to Glutamic acid (The underline nucleotides are the introduced mutations).

To construct the STAT1^{S727D} variant in the pRosetta-STAT1 plasmid, mutations were introduced using the primers 5″-CGTCAAA CTCCTCAGGATCCATGGGGAGCAGGTTGT-3″ and 5″-CCCCATGGA TCCTGAGGAGTTTGACGAGGTGTCTCG-3″, replacing Serine 727 in STAT1 to Aspartic acid (The underline nucleotides are the introduced mutations). To construct the STAT1-GFP-FKBP12 F36V (dTA G13), the EGFP-FKBP12 F36V fragment was first amplified from EGFP-dtag-HA plasmid using primers (5″-GAATTCGACAGTATGA TGAACACAGTAGCCATGGTGAGCAAGGGCGAGGAG-3″, 5″-AGGC TGAAGTTAGTAGCTCCGCTTCCGCTAGGTGCATAGTCCGGGACAT CATACG-3″) and inserted into pRosetta-STAT1 plasmid in frame with the STAT1 C-terminus by the SLIC method (Jeong *et al*, 2012).

All plasmid inserts were verified by DNA sequencing. Expression plasmids were co-transfected with lentiviral packaging plasmid psPAX2 (Addgene #12260), and envelope plasmid pMD2.G (Addgene #12259) into HEK293T cells at a molar ratio of 4:3:1, respectively. Lentiviral particles were harvested as described (Dull *et al*, 1998). The lentivirus containing the desired construct was then transduced into HeLa cells (see below in CRISPR/Cas9 knockout and lentivirus packaging section).

Transcriptional memory assay

Cells were primed with IFNγ (Merck) or left untreated for 24 h, followed by IFNγ washout with DPBS (ThermoFisher) and trypsinization by TrypLE (ThermoFisher) to harvest cells. Cells were cultured with fresh medium for another 48 h unless stated otherwise. Next, naïve and primed cells were induced by IFNγ for 24 h. After 24 h, cells were trypsinized and harvested, and the pellets were processed for subsequent experiments.

ATAC-seq

The ATAC-seq procedure was adapted from Omni-ATAC protocol (Corces *et al*, 2017). Briefly, 50,000 naive or primed cells treated with IFNγ for 1 and 3 h and nontreated cells were centrifuged for 5 min at 500 *g*, resuspended in 50 μl of cold ATAC lysis buffer (10 mM Tris–HCl pH 7.4; 10 mM NaCl; 3 mM $MgCl_2$; 0.1% NP-40; 0.1% Tween-20 and 0.01% Digitonin), and incubated on ice for 3 min. Next, 1 ml wash buffer was added to the pellets (10 mM Tris–HCl pH 7.4; 10 mM NaCl; 3 mM MgCl2; 0.1% Tween-20), cells were resuspended and immediately centrifuged for 10 min at 500 *g* at 4°C. Next cell pellets were resuspended in 50 μl tagmentation reaction buffer [25 μl 2 × TD buffer, 16.5 μl 1× PBS, 0.1% Tween-20, 0.01% Digitonin, 5.4 μl nuclease-free water and 2.5 μl TDE1 (Tn5 enzyme, Illumina)] followed by incubation at 37°C for 30 min. DNA was then purified using MinElute PCR Purification Kit (QIAGEN) (10 μl elution).

DNA library preparation was performed as previously reported (Siwek *et al*, 2020). In brief, the purified DNA was amplified by Q5 Hot start DNA polymerase using described indexing primers (Buenrostro *et al*, 2013). The thermal cycling process was programmed as

 © 2023 The Authors

follows: 72°C for 5 min and 98°C for 30 s, and five cycles of (98°C 10 s; 63°C 30 s; 72°C 1 min); followed by subsequent library quantification using qPCR with the following program: 95°C for 30 s, 95°C for 10 s, 58°C for 30 s, 72°C for 1 min, and additional PCR amplification that is needed for each sample to reach 1/3 of saturated signal. DNA library was purified and size selected using a double-sided bead purification protocol with AMPure XP beads that removes both unwanted small and large fragments (Beckman Coulter). The QuBit dsDNA HS Assay (Thermo Fisher Scientific) was used to determine DNA concentration according to the manufacturer's protocol. Fragment size was estimated by DNA TapeStation (Agilent High Sensitivity D1000) prior to mixing of multiplexed libraries diluted to 2 nM concentration (calculated based the on Qubit dsDNA HS Assay Kit) for sequencing using the NextSeq 500/550 v2.5 kit (Illumina, 75 bp single-end reads). Finally, sequenced reads were mapped to the human genome (hg38) using bowtie2 (Langmead & Salzberg, 2012). Coverage bigwig files were generated using bamCompare (Ramírez *et al*, 2016), with 50 bp bin size. Data were normalized to reads per million (RPM).

CRISPRa-SAM

CRISPRa-SAM transfection was performed as described (Konermann *et al*, 2014) with the following modifications: Guide RNAs (gRNAs) for GBP1 and GBP5 were designed using GPP Web Portal (Broad Institute). gRNAs were designed 200–300 bases upstream of the TSS. All oligo sequences can be found in Appendix Table S1. Cells were transiently transfected with Cas9 component plasmid (Addgene #61425), gRNAs plasmid (Addgene #73795), and MS2-P65-HSF1 activator plasmid (Addgene #61426) at a molar ratio of 1:1:1, respectively. Lipofectamine LTX (ThermoFisher) transfection was performed according to the manufacturer's protocol. After 4 h, the cells were cultured in fresh medium and incubated for 2 days at 37°C. Transfected cells were harvested and processed for further experiments.

CRISPR/Cas9 knockout and lentivirus packaging and transfection

To mutate STAT and IRF genes, gRNAs were selected from the Toronto KnockOut (TKO) CRISPR Library (Appendix Table S1). The lentiCRISPR plasmids (Addgene #52961) with cloned gRNAs were co-transfected (as described above) with lentiviral packaging plasmid psPAX2 (Addgene #12260) and envelope plasmid pMD2.G (Addgene #12259) into HEK293T cells at a molar ratio of 4:3:1, respectively. Cells were incubated for 3 days at 37°C, prior to collecting the medium containing the virus and filtering through a 0.45 μm filter. HeLa cells were incubated in a medium containing 8 μg/ml of polybrene (Merck) for 1 h, then infected with filtered viruses carrying Cas9 with gRNAs targeting STAT and IRF genes. The cells were left to grow for 48 h followed by selection with puromycin (1 μg/ml).

RT–qPCR

RNA was extracted using TRIzol (Invitrogen) based on the manufacturer's instructions. DNA was removed with DNase I (New England Biolabs) and RNA was purified using Monarch® RNA Cleanup Kit (New England Biolabs). Complementary DNA (cDNA) was generated using High-Capacity RNA-to-cDNA Kit (Applied Biosystems) and the cDNAs were diluted 10-fold prior to qPCR measurements. The qPCR assay was performed with iTaq Universal SYBR Green Supermix (BioRad) on CFX384 Real-Time System machine (BioRad). qPCR primers are listed in Appendix Table S1. All experiments were performed in technical and biological triplicates. Primer efficiency and qPCR quantification were analyzed as previously described (Siwek *et al*, 2020).

Immunoblotting

Cell pellets were resuspended in protein sample buffer (125 mM Tris–HCl pH 6.8, 10% Glycerol, 1% SDS, 0.2% (w/v) Orange G, 5% β-mercaptoethanol) and incubated in 98°C for 5 min. Benzonase (50 U) was added to the lysates and incubated at room temperature for 30 min, followed by incubation at 98°C for 5- and 10-min centrifugation. Soluble extracts were separated on a 10% or 12% SDS–PAGE gel (BioRad), then transferred to nitrocellulose membranes (BioRad Transblot Turbo), blocked with Intercept (PBS) Blocking Buffers (LI-COR) for 1 h and incubated overnight with primary antibodies at 4°C. The following day, blots were washed three times with TBST (20 mM Tris–HCl pH 7.5, 150 mM NaCl, 0.1% Tween-20) and incubated with secondary antibodies for 1 h, with subsequent three times TBST washing. The blots were analyzed by Odyssey Imaging System (LI-COR) and quantified using ImageStudio.

CUT&RUN

For CUT&RUN (preprint: Meers *et al*, 2019a), samples were processed using CUTANA™ CUT&RUN kit (EpiCypher) according to the manufacturer's instructions. In brief, cells were fixed with 1% formaldehyde for 1 min at room temperature, followed by quenching in 25 ml of 125 mM glycine in PBS. Cells were harvested by low-speed centrifugation, 500,000 cells were washed with washing buffer, then incubated with preactivated Concanavalin A-coated beads for 10 min at room temperature, followed by overnight incubation at 4°C with antibodies (0.5 μg) in antibody-buffer containing 0.01% digitonin. Next, ConA beads bound cells were washed twice with permeabilization buffer containing 0.01% digitonin, then incubated with protein A-MNase fusion for 10 min at room temperature and washed to remove unbound protein A-MNase. Cleavage was performed at 4°C for 2 h by the addition of calcium chloride to a final concentration of 100 mM. After incubation, the reaction was stopped by the addition of the STOP buffer (containing fragmented genomic *E. coli* DNA as spike-in). Fragmented DNA samples were extracted after 10 min of incubation at 37°C, followed by phenol-chloroform extraction. For preparation of DNA libraries, fragmented DNA was processed using NEBNext Ultra II DNA Library Prep Kit for Illumina (New England Biolabs) according to the manufacturer's instructions. Next, the material was purified and size selected using a double-sided bead purification protocol with AMPure XP beads (Beckman Coulter). Purified multiplexed libraries were diluted to 2 nM concentration (calculated based on the Qubit dsDNA HS Assay Kit) for sequencing using NextSeq 500/550 v2.5 Kit (Illumina, 35 bp paired-end reads). Finally, sequenced reads were mapped to the human genome (hg38) using bowtie2 (Langmead & Salzberg, 2012). Coverage bigwig files were generated using bamCompare (Ramírez *et al*, 2016), with 50 bp bin size. Coverage data were normalized to reads per million (RPM).

Immunofluorescence

Immunofluorescence protocols were adopted by Bodor *et al* (2012). In brief, cells were fixed on poly-l-lysine coated glass coverslips with 4% formaldehyde (Thermo Scientific) for 10 min followed by

© 2023 The Authors

permeabilization with in PBS with 0.1% v/v Triton-X-100 (PBS-TX) (ThermoFisher), blocked for 30 min at 37°C, then incubated with STAT1 antibody (1:50, Cell Signaling Technology) for 1 h at 37°C. Coverslips were washed with PBS-TX and incubated with fluorescein-conjugated anti-rabbit IgG (1:200, Rockland Immunochemicals) for 30 min at 37°C. Nuclei were stained using DAPI (Merck). Coverslips were mounted in Mowiol and stored at 4°C until imaging.

Microcopy

Imaging was performed on Leica Microsystems DMI 6000B inverted-light microscope at 40× magnification using a 1.4 NA oil objective (HC PLAN APO) to capture 0.2 μm Z-stacks. Images were quantified using ImageJ macros. CRaQ is described previously (Bodor *et al*, 2012), and modified to calculate the whole DAPI signal as region of interest (ROI). The macro quantifies the median of whole cell STAT1 levels and nuclear STAT1 levels (using DAPI as mask).

Live cell imaging was performed on Leica Microsystems DMI 6000B inverted-light microscope at 20× magnification, a microscope stage incubator maintained at 37°C in a humidified atmosphere. HeLa STAT1 KO cells constitutively expressing STAT1-GFP-FKB12 F36V (dTAG13) were seeded in chamber slides (ThermoFisher) and grown in Live Cell Imaging solution (ThermoFisher) containing 10% FBS and 1% Penicillin–Streptomycin (ThermoFisher, 15140-122). Vybrant DyeCycle Ruby Stain (ThermoFisher) was added for 1 h before imaging at a final concentration of 5 μM to mark cell nuclei. Images were acquired every 10 min immediately after IFNγ induction and quantified using CellProfiler (Carpenter *et al*, 2006).

FACS

Cells were harvested and resuspended in ice-cold conditional medium (1:1 mixture of fresh complete medium and filtered medium collected from proliferating cell cultures) supplemented with 20% Fetal Bovine Serum, 0.25 mg/ml Fungizone (Thermo Fisher Scientific), 1% Penicillin–Streptomycin (ThermoFisher, 15140-122), and filtered through a 5 ml polystyrene round-bottom tubes with cell-strainer caps (Falcon) before sorting (FACSCalibur) (BD Biosciences). Cells were collected in the conditional medium.

Quantification and statistical analysis

RT–qPCR and STAT1CUT&RUN data were collected in triplicate, and IRF1 CUT&RUN data were collected in duplicate (with the exception of the IRF1 primed 3 h data point for which only one replicate is shown). For ATAC-seq one replicate experiment is shown. Standard deviation is reported. Statistical analyses and *P*-value calculations for the RT–qPCR data were performed by one-way or two-way analysis of variance (ANOVA) (GraphPad prism, v9.3.1) and can be found in the figures.

Data availability

The datasets produced in this study are available in the following databases: ATAC-seq: ArrayExpress E-MTAB-12624 https://www.ebi.ac.uk/biostudies/arrayexpress/studies/E-MTAB-12624?accession=E-MTAB-12624; CUT&RUN: ArrayExpress E-MTAB-12625 https://www.ebi.ac.uk/biostudies/arrayexpress/studies/E-MTAB-12625?accession=E-MTAB-12625.

Expanded View for this article is available online.

Acknowledgements

We thank the IGC Gulbenkian FACS facility and the Sir William Dunn School of Pathology Flow Cytometry Facility for support with cell sorting. We thank Amanda Williams, Zoology department, University of Oxford for support with Illumina sequencing. This research was funded in part by an ERC-consolidator grant ERC-2013-CoG-615638 and the Wellcome Trust, Senior Wellcome Research Fellowship [210645/Z/18/Z] to LETJ. For the purpose of Open Access, the author has applied a CC BY public copyright licence to any Author Accepted Manuscript version arising from this submission. SSHT was supported by Fundação para a Ciência e a Tecnologia (FCT) doctoral fellowship PD/BD/128438/2017. LETJ receives salary support from the University of Oxford, Department of Biochemistry. WS was supported by an FCT postdoctoral fellowship (SFRH/BPD/117179/2016) and a Marie Sklodowska-Curie individual fellowship (101025900).§

Author contributions

Sahar SH Tehrani: Conceptualization; data curation; formal analysis; investigation; writing – original draft; writing – review and editing. **Pawel Mikulski:** Data curation; formal analysis; supervision; investigation; writing – review and editing. **Izma Abdul-Zani:** Investigation; project administration. **João F Mata:** Investigation; project administration. **Wojciech Siwek:** Conceptualization; data curation; formal analysis; supervision; investigation; writing – original draft. **Lars ET Jansen:** Conceptualization; data curation; supervision; funding acquisition; writing – original draft; project administration; writing – review and editing.

Disclosure and competing interests statement

The authors declare that they have no conflict of interest.

References

Antonczyk A, Krist B, Sajek M, Michalska A, Piaszyk-Borychowska A, Plens-Galaska M, Wesoly J, Bluyssen HAR (2019) Direct inhibition of IRF-dependent transcriptional regulatory mechanisms associated with disease. *Front Immunol* 10: 1176

Bancerek J, Poss ZC, Steinparzer I, Sedlyarov V, Pfaffenwimmer T, Mikulic I, Dölken L, Strobl B, Müller M, Taatjes DJ *et al* (2013) CDK8 kinase phosphorylates transcription factor STAT1 to selectively regulate the interferon response. *Immunity* 38: 250 – 262

Bodor DL, Rodríguez MG, Moreno N, Jansen LET (2012) Analysis of protein turnover by quantitative SNAP-based pulse-chase imaging. *Curr Protoc Cell Biol* Chapter 8: Unit8.8

Buenrostro JD, Giresi PG, Zaba LC, Chang HY, Greenleaf WJ (2013) Transposition of native chromatin for fast and sensitive epigenomic profiling of open chromatin, DNA-binding proteins and nucleosome position. *Nat Methods* 1012: 1213 – 1218

Carpenter AE, Jones TR, Lamprecht MR, Clarke C, Kang IH, Friman O, Guertin DA, Chang JH, Lindquist RA, Moffat J *et al* (2006) CellProfiler: Image

§Correction added on 17 July 2023 after first online publication: Funding information for WS has been added.

 © 2023 The Authors

analysis software for identifying and quantifying cell phenotypes. *Genome Biol* 7: 1–11

Chavez A, Tuttle M, Pruitt BW, Ewen-Campen B, Chari R, Ter-Ovanesyan D, Haque SJ, Cecchi RJ, Kowal EJK, Buchthal J *et al* (2016) Comparison of Cas9 activators in multiple species. *Nat Methods* 13: 563–567

Cheon H, Stark GR (2009) Unphosphorylated STAT1 prolongs the expression of interferon-induced immune regulatory genes. *Proc Natl Acad Sci USA* 106: 9373–9378

Corces MR, Trevino AE, Hamilton EG, Greenside PG, Sinnott-Armstrong NA, Vesuna S, Satpathy AT, Rubin AJ, Montine KS, Wu B *et al* (2017) An improved ATAC-seq protocol reduces background and enables interrogation of frozen tissues. *Nat Methods* 1410: 959–962

Darnell JE, Kerr IM, Stark GR (1994) Jak-STAT pathways and transcriptional activation in response to IFNs and other extracellular signaling proteins. *Science* 264: 1415–1421

Delgoffe GM, Vignali DAA (2013) STAT heterodimers in immunity: a mixed message or a unique signal? *JAKSTAT* 2: e23060

Ding Y, Liu N, Virlouvet L, Riethoven JJ, Fromm M, Avramova Z (2013) Four distinct types of dehydration stress memory genes in *Arabidopsis thaliana*. *BMC Plant Biol* 13: 1–11

Dull T, Zufferey R, Kelly M, Mandel RJ, Nguyen M, Trono D, Naldini L (1998) A third-generation lentivirus vector with a conditional packaging system. *J Virol* 72: 8463–8471

D'Urso A, Brickner JH (2017) Epigenetic transcriptional memory. *Curr Genet* 63: 435–439

D'Urso A, Takahashi Y, Xiong B, Marone J, Coukos R, Randise-Hinchliff C, Wang J-P, Shilatifard A, Brickner JH (2016) Set1/COMPASS and mediator are repurposed to promote epigenetic transcriptional memory. *Elife* 5: e16691

Fanucchi S, Fok ET, Dalla E, Shibayama Y, Börner K, Chang EY, Stoychev S, Imakaev M, Grimm D, Wang KC *et al* (2019) Immune genes are primed for robust transcription by proximal long noncoding RNAs located in nuclear compartments. *Nat Genet* 51: 138–150

Gialitakis M, Arampatzi P, Makatounakis T, Papamatheakis J (2010) Gamma interferon-dependent transcriptional memory via relocalization of a gene locus to PML nuclear bodies. *Mol Cell Biol* 30: 2046–2056

Giménez CA, Ielpi M, Mutto A, Grosembacher L, Argibay P, Pereyra-Bonnet F (2016) CRISPR-on system for the activation of the endogenous human INS gene. *Gene Ther* 236: 543–547

Jeong JY, Yim HS, Ryu JY, Lee HS, Lee JH, Seen DS, Kang SG (2012) One-step sequence- and ligation-independent cloning as a rapid and versatile cloning method for functional genomics studies. *Appl Environ Microbiol* 78: 5440–5443

Kamada R, Yang W, Zhang Y, Patel MC, Yang Y, Ouda R, Dey A, Wakabayashi Y, Sakaguchi K, Fujita T *et al* (2018) Interferon stimulation creates chromatin marks and establishes transcriptional memory. *Proc Natl Acad Sci USA* 115: E9162–E9171

Kleinnijenhuis J, Quintin J, Preijers F, Joosten LAB, Ifrim DC, Saeed S, Jacobs C, van Loenhout J, de Jong D, Stunnenberg HG *et al* (2012) Bacille Calmette-Guérin induces NOD2-dependent nonspecific protection from reinfection via epigenetic reprogramming of monocytes. *Proc Natl Acad Sci* 109: 17537–17542.

Konermann S, Brigham MD, Trevino AE, Joung J, Abudayyeh OO, Barcena C, Hsu PD, Habib N, Gootenberg JS, Nishimasu H *et al* (2014) Genome-scale transcriptional activation by an engineered CRISPR-Cas9 complex. *Nature* 517: 583–588

Lämke J, Brzezinka K, Altmann S, Bäurle I (2016) A hit-and-run heat shock factor governs sustained histone methylation and transcriptional stress memory. *EMBO J* 35: 162–175

Langmead B, Salzberg SL (2012) Fast gapped-read alignment with Bowtie 2. *Nat Methods* 9: 357–359

Lau CM, Adams NM, Geary CD, El WO, Rapp M, Pritykin Y, Leslie CS, Sun JC (2018) Epigenetic control of innate and adaptive immune memory. *Nat Immunol* 19: 963–972

Light WH, Freaney J, Sood V, Thompson A, D'Urso A, Horvath CM, Brickner JH (2013) A conserved role for human Nup98 in altering chromatin structure and promoting epigenetic transcriptional memory. *PLoS Biol* 11: e1001524

Liu N, Avramova Z (2016) Molecular mechanism of the priming by jasmonic acid of specific dehydration stress response genes in Arabidopsis. *Epigenetics Chromatin* 91: 1–23

Meers MP, Bryson T, Henikoff S (2019a) A streamlined protocol and analysis pipeline for CUT&RUN chromatin profiling. *bioRxiv* https://doi.org/10.1101/569129 [PREPRINT]

Meers MP, Bryson TD, Henikoff JG, Henikoff S (2019b) Improved CUT&RUN chromatin profiling tools. *Elife* 8: e46314

Migita K, Komori A, Torigoshi T, Maeda Y, Izumi Y, Jiuchi Y, Miyashita T, Nakamura M, Motokawa S, Ishibashi H (2011) CP690,550 inhibits oncostatin M-induced JAK/STAT signaling pathway in rheumatoid synoviocytes. *Arthritis Res Ther* 13: 1–10

Moazed D (2011) Mechanisms for the inheritance of chromatin states. *Cell* 146: 510–518

Mogensen TH (2018) IRF and STAT transcription factors - from basic biology to roles in infection, protective immunity, and primary immunodeficiencies. *Front Immunol* 9: 3047

Nabet B, Roberts JM, Buckley DL, Paulk J, Dastjerdi S, Yang A, Leggett AL, Erb MA, Lawlor MA, Souza A *et al* (2018) The dTAG system for immediate and target-specific protein degradation. *Nat Chem Biol* 14: 431–441

Natoli G, Ostuni R (2019) Adaptation and memory in immune responses. *Nat Immunol* 207: 783–792

Netea MG, Quintin J, Van Der Meer JWM (2011) Trained immunity: a memory for innate host defense. *Cell Host Microbe* 9: 355–361

Netea MG, Domínguez-Andrés J, Barreiro LB, Chavakis T, Divangahi M, Fuchs E, Joosten LAB, van der Meer JWM, Mhlanga MM, Mulder WJM *et al* (2020) Defining trained immunity and its role in health and disease. *Nat Rev Immunol* 20: 375–388

Ni Z, Karaskov E, Yu T, Callaghan SM, Der S, Park DS, Xu Z, Pattenden SG, Bremner R (2005) Apical role for BRG1 in cytokine-induced promoter assembly. *Proc Natl Acad Sci USA* 102: 14611–14616

Peignier A, Parker D (2020) Trained immunity and host-pathogen interactions. *Cell Microbiol* 22: e13261

Quintin J, Saeed S, Martens JHA, Giamarellos-Bourboulis EJ, Ifrim DC, Logie C, Jacobs L, Jansen T, Kullberg BJ, Wijmenga C *et al* (2012) *Candida albicans* infection affords protection against reinfection via functional reprogramming of monocytes. *Cell Host Microbe* 12: 223–232

Ramana CV, Chatterjee-Kishore M, Nguyen H, Stark GR (2000) Complex roles of Stat1 in regulating gene expression. *Oncogene* 19: 2619–2627

Ramana CV, Gil MP, Schreiber RD, Stark GR (2002) Stat1-dependent and -independent pathways in IFN-γ-dependent signaling. *Trends Immunol* 23: 96–101

Ramírez F, Ryan DP, Grüning B, Bhardwaj V, Kilpert F, Richter AS, Heyne S, Dündar F, Manke T (2016) deepTools2: a next generation web server for deep-sequencing data analysis. *Nucleic Acids Res* 44: W160–W165

Ramsauer K, Farlik M, Zupkovitz G, Seiser C, Kröger A, Hauser H, Decker T (2007) Distinct modes of action applied by transcription factors STAT1 and IRF1 to initiate transcription of the IFN-gamma-inducible gbp2 gene. *Proc Natl Acad Sci USA* 104: 2849–2854

© 2023 The Authors

Rawlings JS, Rosler KM, Harrison DA (2004) The JAK/STAT signaling pathway. *J Cell Sci* 117: 1281–1283

Sanjana NE, Shalem O, Zhang F (2014) Improved vectors and genome-wide libraries for CRISPR screening. *Nat Methods* 11: 783–784

Schindler C, Fu XY, Improta T, Aebersold R, Darnell JE (1992) Proteins of transcription factor ISGF-3: one gene encodes the 91-and 84-kDa ISGF-3 proteins that are activated by interferon alpha. *Proc Natl Acad Sci USA* 89: 7836–7839

Schroder K, Hertzog PJ, Ravasi T, Hume DA (2004) Interferon-gamma: an overview of signals, mechanisms and functions. *J Leukoc Biol* 75: 163–189

Sif S, Saurin AJ, Imbalzano AN, Kingston RE (2001) Purification and characterization of mSin3A-containing Brg1 and hBrm chromatin remodeling complexes. *Genes Dev* 15: 603–618

Siwek W, Tehrani SSH, Mata JF, Jansen LET (2020) Activation of clustered IFNγ target genes drives Cohesin-controlled transcriptional memory. *Mol Cell* 80: 396–409

Song J, Angel A, Howard M, Dean C (2012) Vernalization - a cold-induced epigenetic switch. *J Cell Sci* 125: 3723–3731

Sood V, Cajigas I, D'Urso A, Light WH, Brickner JH (2017) Epigenetic transcriptional memory of *GAL* genes depends on growth in glucose and the Tup1 transcription factor in *Saccharomyces cerevisiae*. *Genetics* 206: 1895–1907

Sump B, Brickner DG, D'Urso A, Kim SH, Brickner JH (2022) Mitotically heritable, RNA polymerase II-independent H3K4 dimethylation stimulates INO1 transcriptional memory. *Elife* 11: e77646

Verma D, Parasa VR, Raffetseder J, Martis M, Mehta RB, Netea M, Lerm M (2017) Anti-mycobacterial activity correlates with altered DNA methylation pattern in immune cells from BCG-vaccinated subjects. *Sci Rep* 71: 1–10

Wen Z, Zhong Z, Darnell JE (1995) Maximal activation of transcription by statl and stat3 requires both tyrosine and serine phosphorylation. *Cell* 82: 241–250

Zakharova N, Lymar ES, Yang E, Malik S, Zhang JJ, Roeder RG, Darnell JE (2003) Distinct transcriptional activation functions of STAT1α and STAT1β on DNA and chromatin templates. *J Biol Chem* 278: 43067–43073

Zhao Z, Zhang Z, Li J, Dong Q, Xiong J, Li Y, Lan M, Li G, Zhu B (2020) Sustained TNF-α stimulation leads to transcriptional memory that greatly enhances signal sensitivity and robustness. *Elife* 9: 1–27

License: This is an open access article under the terms of the Creative Commons Attribution License, which permits use, distribution and reproduction in any medium, provided the original work is properly cited.

 © 2023 The Authors

Expanded View Figures

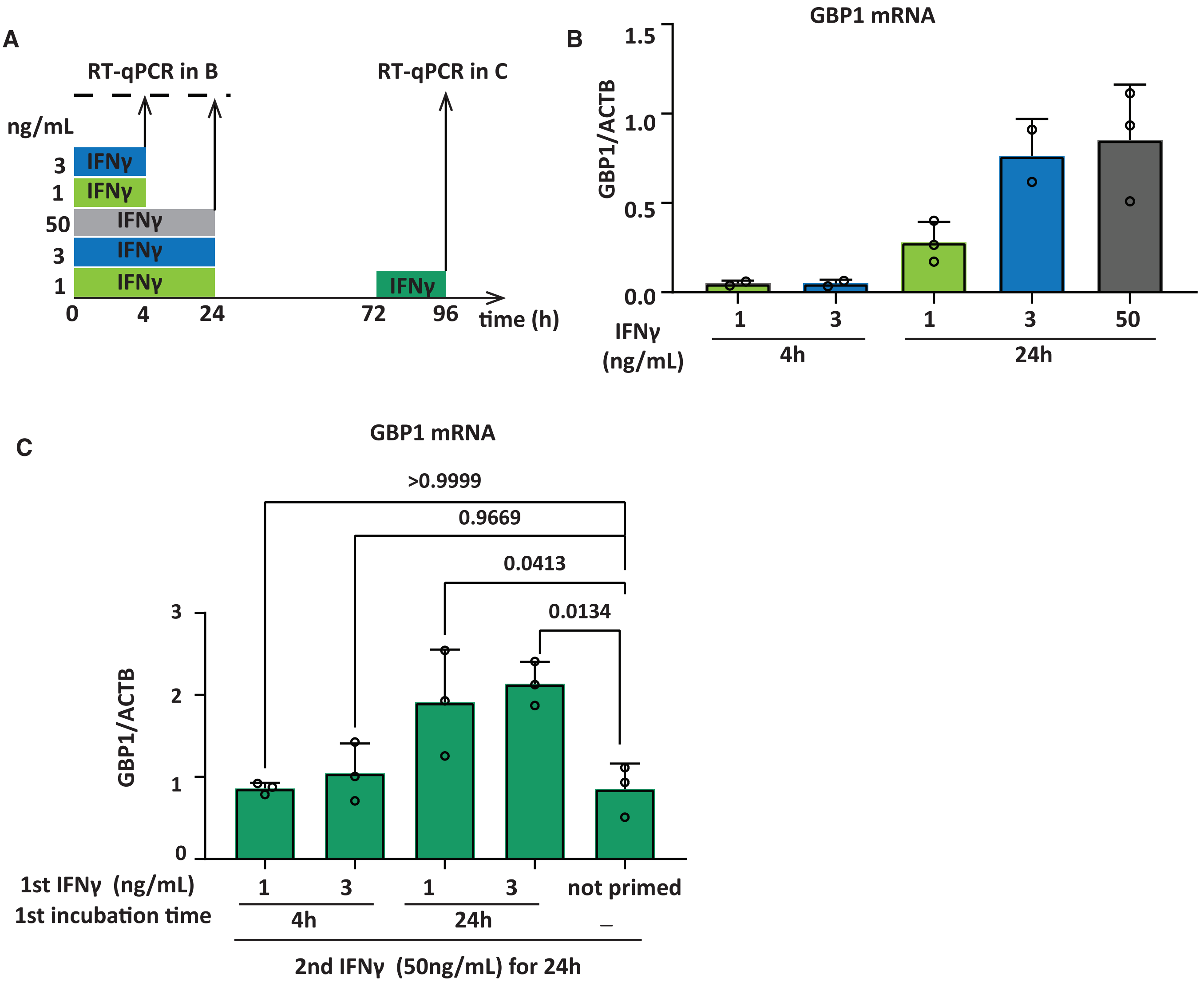

Figure EV1. Titration of minimal IFNγ pulse to prime GBP1.

A Experimental outline of GBP1 priming with different concentrations of IFNγ and incubation times. HeLa cells were induced with 1, 3, or 50 ng/ml of IFNγ for 4 and 24 h, followed by IFNγ washout.

B, C After 48 h, naïve and primed cells were induced with IFNγ (50 ng/ml) for 24 h and harvested for GBP1 mRNA analysis by RT–qPCR after induction (B) and reinduction (C), normalized to ACTB mRNA level. Statistical significance was determined using Ordinary one-way ANOVA. Data are shown as mean (error bars, SD; *n* = 3 biological replicates).

Source data are available online for this figure.

© 2023 The Authors

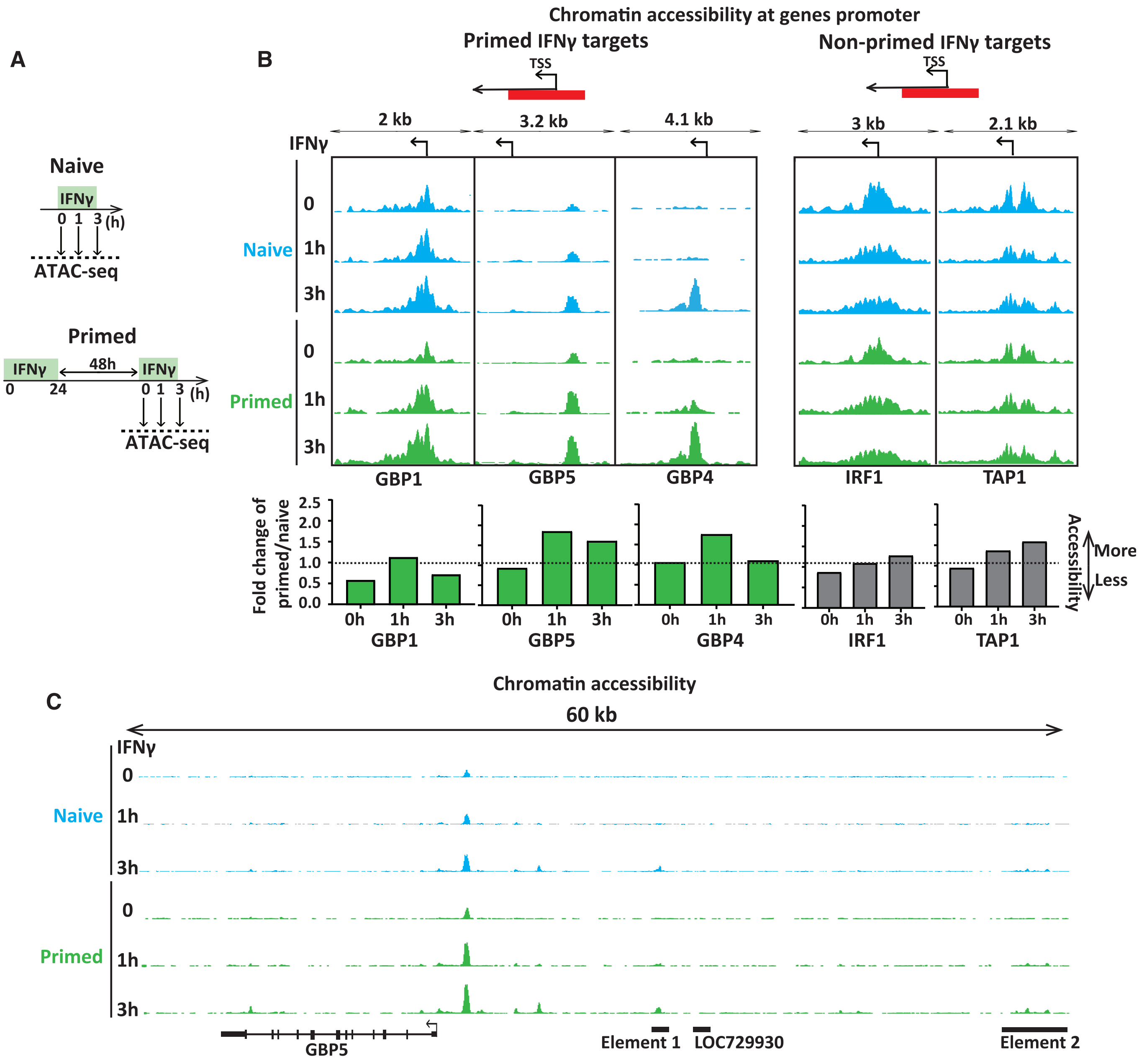

Figure EV2. Promoter accessibility of GBP genes upon IFNγ stimulation.

A Scheme describing chromatin accessibility (ATAC-seq) experiment. HeLa cells were primed with IFNγ for 24 h, followed by IFNγ washout. After 48 h, naïve and primed cells were induced by IFNγ for 1 and 3 h. Cells were harvested at indicated time points and processed for ATAC-seq.

B Results of sequenced reads were mapped to the human genome (hg38), and coverage data is displayed as reads per million (RPM) at equal scaling for two genes showing priming, GBP1, GBP4, and GBP5 (Left) and two IFNγ-induced genes, IRF1 and TAP1 (Right). The data across samples are scaled equally for each locus.

C No nearby enhancers accessible in primed cells. Representation of processed data for ATAC-seq at the GBP5 gene and upstream region. Sequenced reads were mapped to the human genome (hg38), and coverage data are displayed as reads per million (RPM) at equal scaling.

Source data are available online for this figure.

 © 2023 The Authors

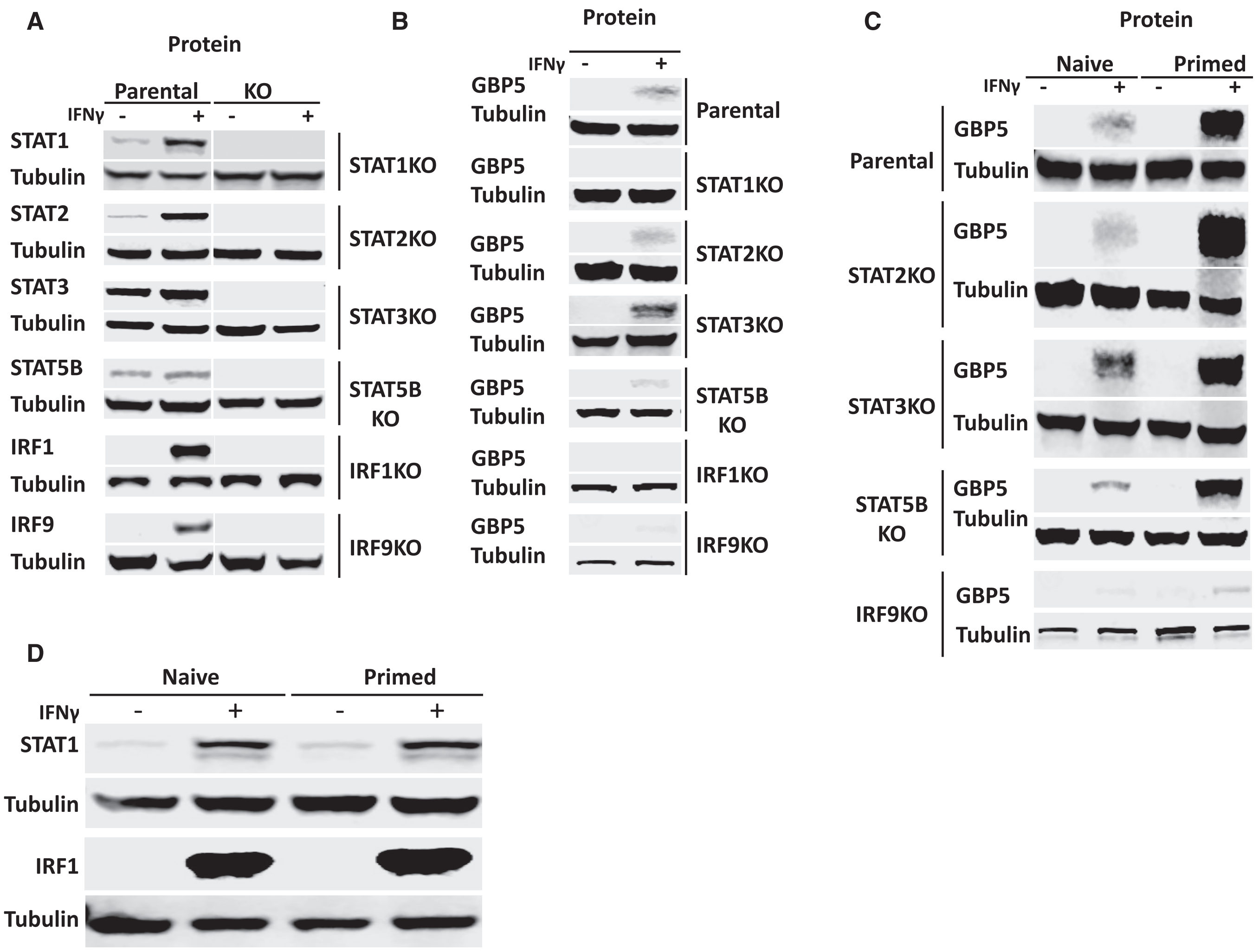

Figure EV3. Knockout cell lines of all relevant STAT and IRF genes to determine the requirement of GBP5 expression.

Stable CRISPR knockouts were generated for indicated genes in HeLa cells. Knockout (KO) cells and their parental controls (WT) were induced with IFNγ for 24 h or left untreated.

A–C Immunoblots probing for (A) STAT and IRF transcription factors to confirm knockout status, (B) probing for the effect on GBP5 expression (C) probing the effect on GBP5 priming. Experiment performed as outlined in Fig 1A. α-Tubulin (Tubulin) was used as a loading control. Note that GBP5 and Tubulin blot for parental cells is as in Fig 1C.

D HeLa cells were subjected to IFNγ induction and reinduction regime as outlined in Fig 1A with 2 days recovery time (primed state) after IFNγ washout. Cell extracts were prepared at indicated time points, processed for western blotting, and probed for STAT1, IRF1. α-Tubulin (Tubulin) as a loading control.

Source data are available online for this figure.

© 2023 The Authors

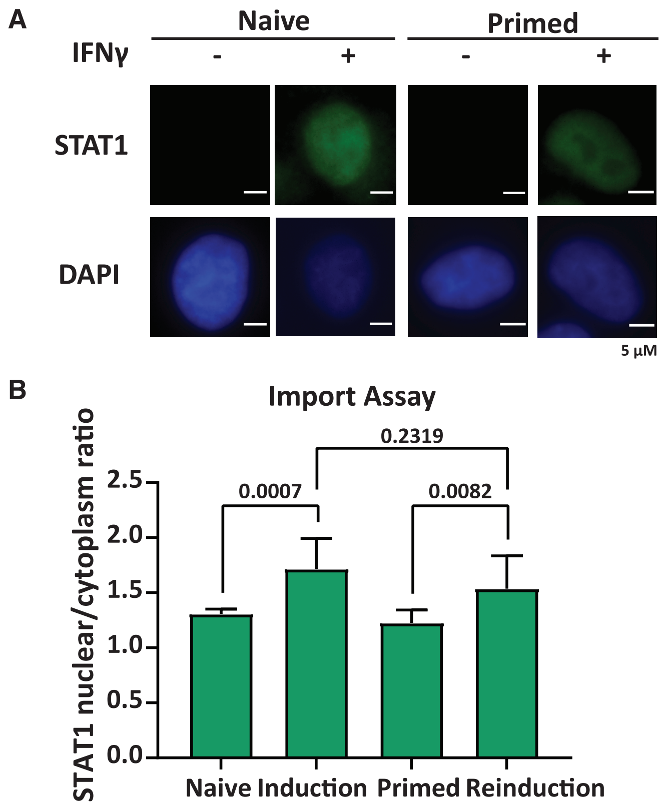

Figure EV4. Priming does not change rate of STAT1 nuclear import.

A Constitutive STAT1 expressing cells were subjected to IFNγ induction and reinduction regime as outlined in Fig 1A. Cells were fixed following indicated treatments as per the scheme in Fig 1A, followed by immunostaining for STAT1 and DAPI and imaging. Scale bar is 5 μm.

B Quantification of the ratio of STAT1 in the nucleus over cytoplasm in fixed cells. Statistical significance was determined using Ordinary one-way ANOVA. Data are shown as mean (error bars, SD; n = 10, biological replicates).

Source data are available online for this figure.

 © 2023 The Authors

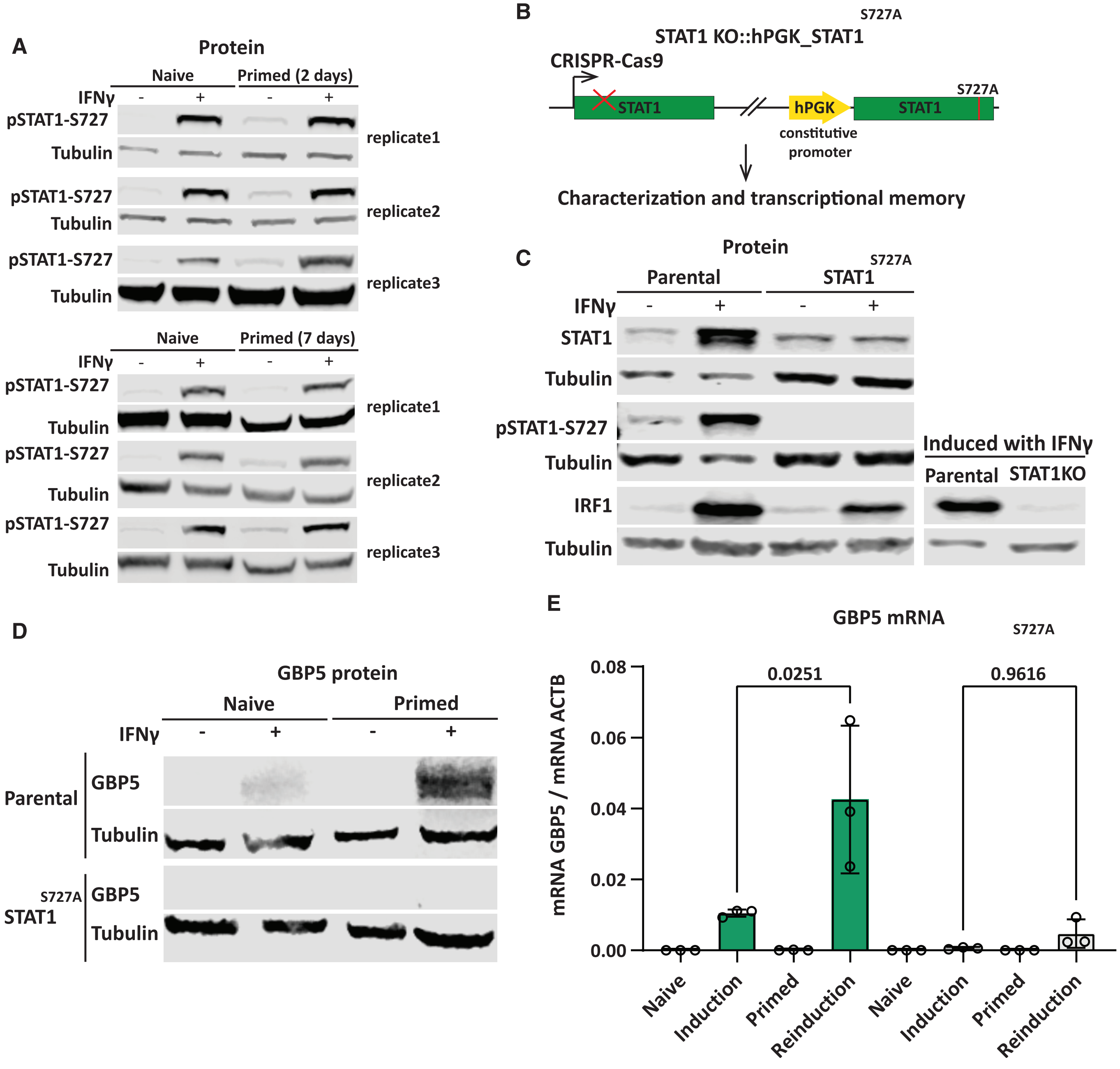

Figure EV5. STAT1 phosphorylation at Ser727 is essential for GBP5 expression.

A Biological replicates of experiments shown in Fig 6A, blots probed for pSTAT1-S727 and α-Tubulin (Tubulin) as a loading control.

B Schematic overview of STAT1 KO cell line rescued with a STAT1 variant with an S727A mutation under a constitutive promoter.

C STAT1KO::STAT1-S727A expressing cells or their parental controls (WT) were induced with IFNγ for 24 h or left untreated and processed for western blotting. Extracts were probed for STAT1, pSTAT1-S727, and IRF1 to confirm the mutation and to assess STAT1S727A function.

D STAT1-S727A expressing cells or their parental controls (WT) were subjected to IFNγ induction and reinduction regime as outlined in Fig 1A, and cell extracts were immunoblotted to determine the GBP5 protein level. α-Tubulin (Tubulin) was used as a loading control.

E In parallel to (D), RNA was isolated and GBP5 mRNA level was determined by RT–qPCR and normalized to ACTB mRNA level. Statistical significance was determined using Ordinary one-way ANOVA. Data are shown as mean (error bars, SD; n = 3 biological replicates).

Source data are available online for this figure.

Appendix

STAT1 is required to establish but not maintain interferon-γ-induced transcriptional memory

Sahar S.H. Tehrani[1,2], Pawel Mikulski[1], Izma Abdul-Zani[1], João F. Mata[2], Wojciech Siwek[1,3,†] and Lars E.T. Jansen[1*]

[1]Department of Biochemistry, University of Oxford, Oxford, UK

[2]Instituto Gulbenkian de Ciência, Oeiras, Portugal

[3]Present address: Department of Molecular Biology, Massachusetts General Hospital, Harvard Medical School, Boston, MA 02114, USA

†Co-corresponding author: *siwek@molbio.mgh.harvard.edu*

*Correspondence: *lars.jansen@bioch.ox.ac.uk* (lead contact)

Table of Contents

Appendix Figure S1 2
Appendix Figure S2 3
Appendix Table S1. list of gRNAs and primes used in this study. 4

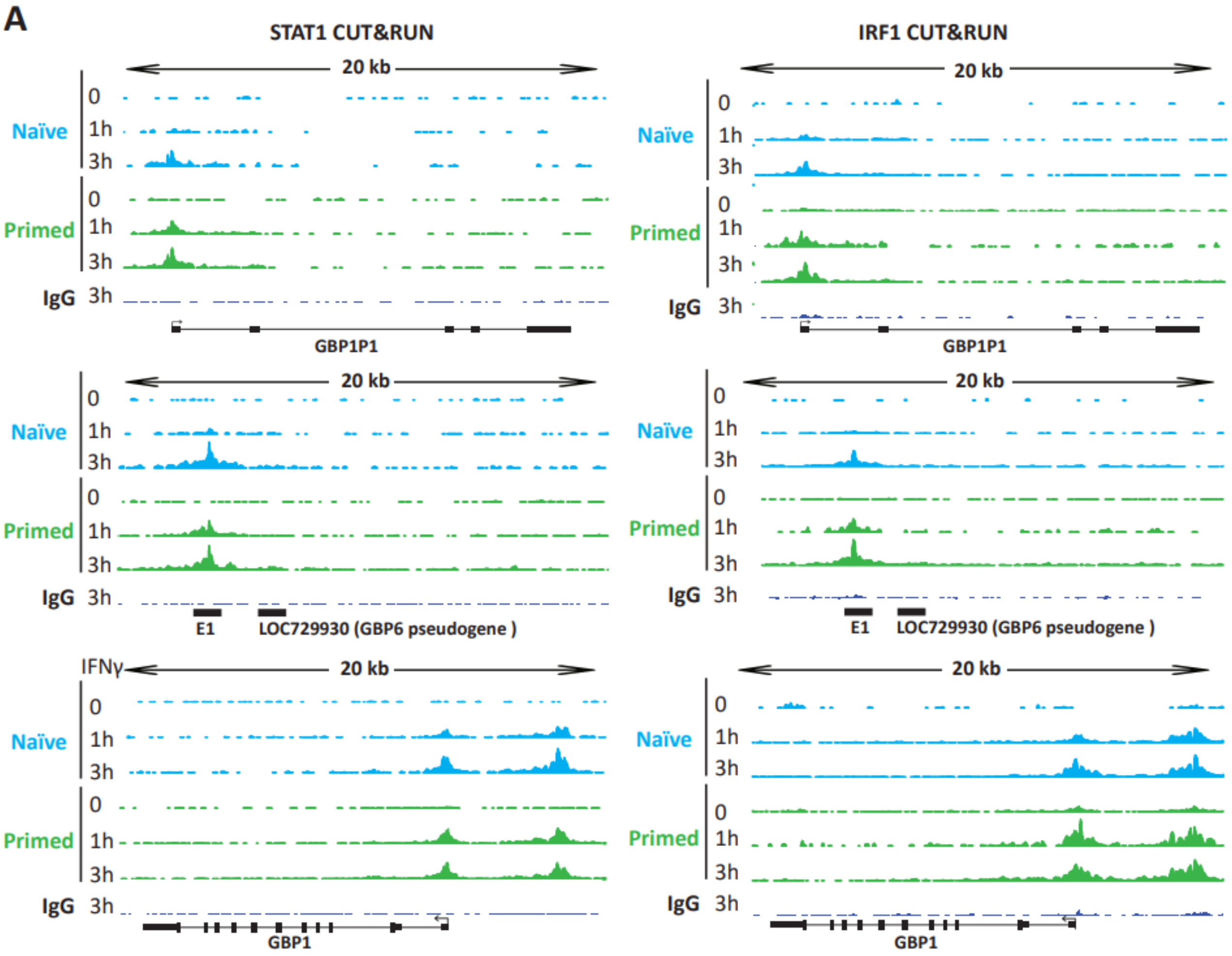

B list of overlap genes in Figure 3G, that showed both faster STAT1 and IRF1 recruitment

	Ensembl	Gene name	description
1	ENSG00000225492	GBP1P1	guanylate binding protein 1 pseudogene
2	ENSG00000154451	GBP5	guanylate binding protein 5 (see Figure 3)
3	ENSG00000162654	GBP4	guanylate binding protein 4 (see Figure 3)
4	ENSG00000237568	nan	LncRNA inside GBP5 gene
5	ENSG00000238081	LOC729930	GBP6 pseudogene, transcription factor binding site is found 2.4 kbp upstream of this site
6	ENSG00000284734	GBP4 (antisense)	novel transcript, antisense to GBP4 (see Figure 3)
7	ENSG00000117228	GBP1	guanylate binding protein 1
8	ENSG00000269588	LGALS13	lectin, galactoside-binding, soluble, 13 (LGALS13) pseudogene
9	ENSG00000253838	nan	lncRNA inside IDO1
10	ENSG00000262151	CIITA (antisense)	novel transcript, antisense to CIITA
11	ENSG00000226025	LGALS17A	galectin 14 pseudogene
12	ENSG00000131203	IDO1	indoleamine 2,3-dioxygenase 1

Appendix Figure S1. Accelerated STAT1 and IRF1 binding in primed genes (A) Representation of processed CUT&RUN data for STAT1 and IRF1 occupancy at the selected GBP genes loci (GBP1P1, LOC729930 and GBP1). **(B)** List of loci showing enhanced STAT1 and IRF1 binding in primed cells as in Figure 3G (−3kb of TSS and +3kb of TTS with a minimum of 1.5-fold difference between primed and naïve upon 1 hour of IFNγ treatment).

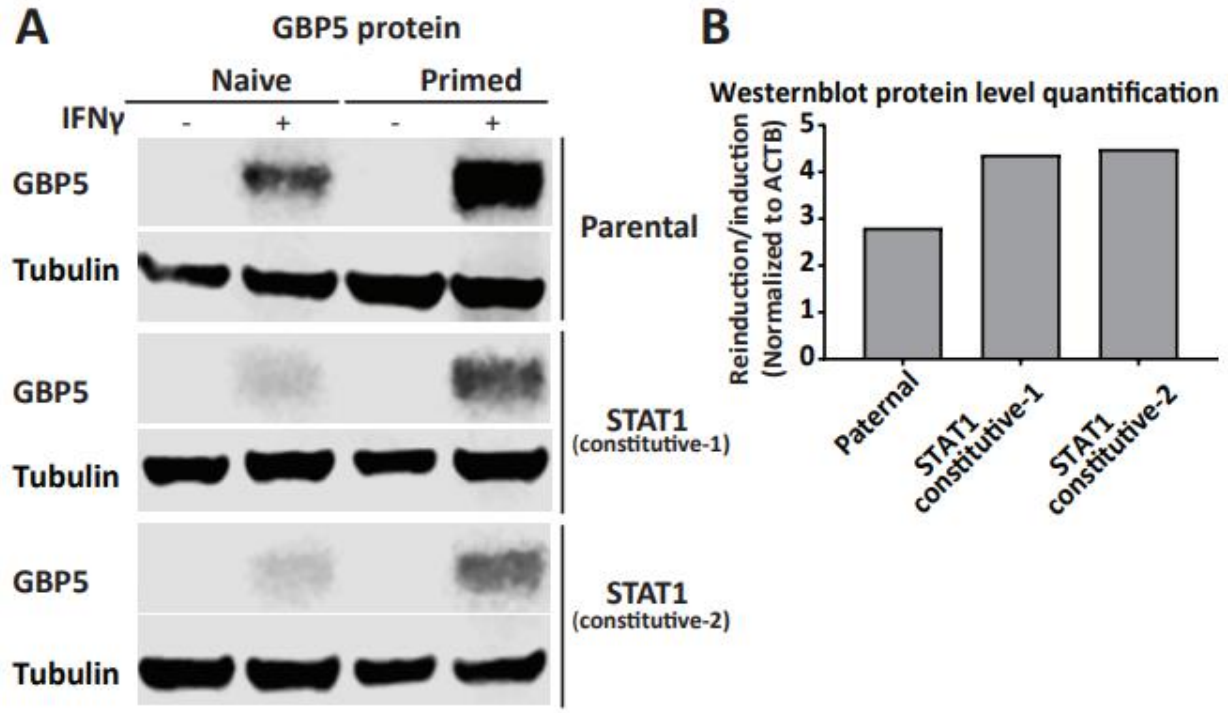

Appendix Figure S2 . Replicates of experiment show in Figure 5D. Two independent clones of STAT1 rescue cells and their parental control were subjected to IFNγ induction and reinduction regime as outlined in Figure 1A. **(A)** Cell extracts were processed for western blotting and probed for GBP5 expression before and after induction and reinduction as indicated in Figure 1A. α-Tubulin (Tubulin) was used as a loading control. Note that GBP5 and Tubulin blot for STAT1 (constitutive-1) are as in Figure 5D, STAT1 (constitutive). **(B)** Western blot signal quantification of blot in 6A. Data are shown as fold change of reinduction to induction.

Appendix Table S1. list of gRNAs and primes used in this study.

**CRISPRa gRNA**	
**Primer name #**	**sequence(5'->3')**
F_GBP1_SAM_gRNA1	CACCGAATTAGTGGAGTGTGCCAG
F_GBP1_SAM_gRNA2	CACCGAAATCTTTAAACCCTCCCAC
F_GBP1_SAM_gRNA3	CACCGTAGAACATGAGTACAACACA
R_GBP1_SAM_gRNA1	AAACCTGGCACACTCCACTAATTC
R_GBP1_SAM_gRNA2	AAACGTGGGAGGGTTTAAAGATTTC
R_GBP1_SAM_gRNA3	AAACTGTGTTGTACTCATGTTCTAC
F_ASCL1_gRNA1	CACCGCGGGAGAAAGGAACGGGAGG
R_ASCL1_gRNA1	AAACCCTCCCGTTCCTTTCTCCCGC
**CRISPR/Cas9**	
**Primer name #**	**sequence(5'->3')**
IRF1B_F	CACCGCATGGCTGGGACATCAACA
IRF9B_F	CACCGAGGGCTCAGCAACATCCATG
STAT1B_F	CACCGAGAACACGAGACCAATGGTG
STAT2B-F	CACCGAAGAATAGCATGGTAGCCT
STAT3A-F	CACCGCTACAGTGACAGCTTCCCAA
STAT5BA-F	CACCGTGCGGCATTATTTATCCCAG
ContorlA-F	CACCGTATTACTGATATTGGTGGG
ControlB-F	CACCGTTCCGCGTTACATAACTTA
IRF1B_R	AAACTGTTGATGTCCCAGCCATGC
IRF9B_R	AAACCATGGATGTTGCTGAGCCCTC
STAT1B_R	AAACCACCATTGGTCTCGTGTTCTC
STAT2B-R	AAACAGGCTACCATGCTATTCTTC
STAT3A-R	AAACTTGGGAAGCTGTCACTGTAGC
STAT5BA-R	AAACCTGGGATAAATAATGCCGCAC
ContorlA-R	AAACCCCACCAATATCAGTAATAC
ControlB-R	AAACTAAGTTATGTAACGCGGAAC
**qPCR**	
Primer name #	sequence(5'->3')
GBP1_qPCR_F	GTGGAACGTGTGAAAGCTGA
GBP1_qPCR_R	CAACTGGACCCTGTCGTTCT
GBP5_trancC_F	TTCAATTTGCCCCGTCTGTG
GBP5_trancC_R	AGGCAGTGTTTCAAGTTGGG
b-actin Forward	AACTGGAACGGTGAAGGTGACAGC
b-actin Reverse	TGGCTTTTAGGATGGCAAGGGAC
