# Supplementary figures and images for: STAT1 is required to establish but not maintain interferon‐γ‐induced transcriptional memory
Source: EMBO J. 2023 Jun 5;42(14):e112259. doi: 10.15252/embj.2022112259 (PMC10350821; doi:10.15252/embj.2022112259)

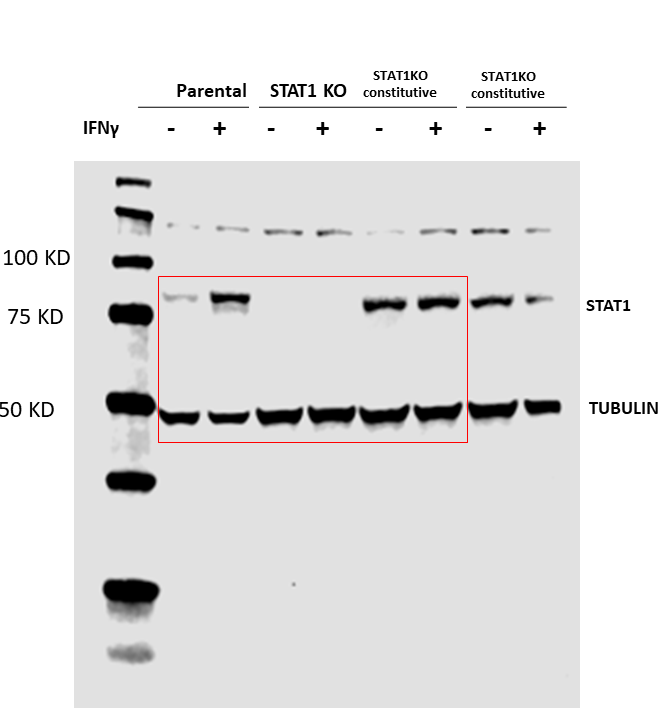

Supplement: Supplementary file 9 — Source Data for Figure 5 [file EMBJ-42-e112259-s007.zip › 5C/5C_STAT1 and Tubulin westernblot-annotated.tif]

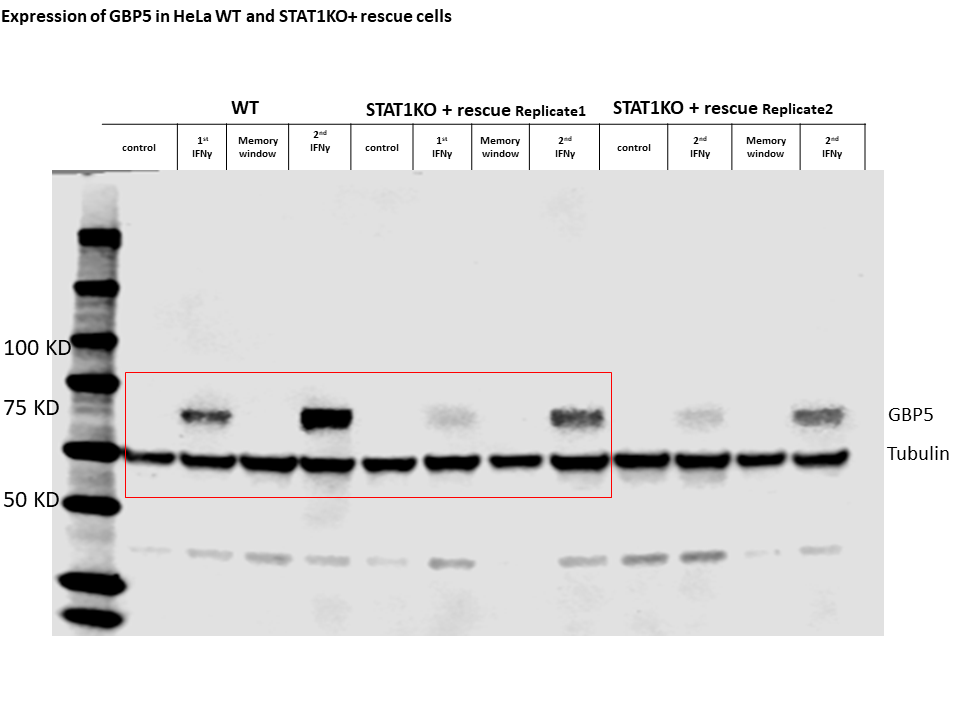

Supplement: Supplementary file 9 — Source Data for Figure 5 [file EMBJ-42-e112259-s007.zip › 5D/5D_GBP5 and Tubulin westernblot_STAT1rescue_WT.06.03.2019-annotated.tif]

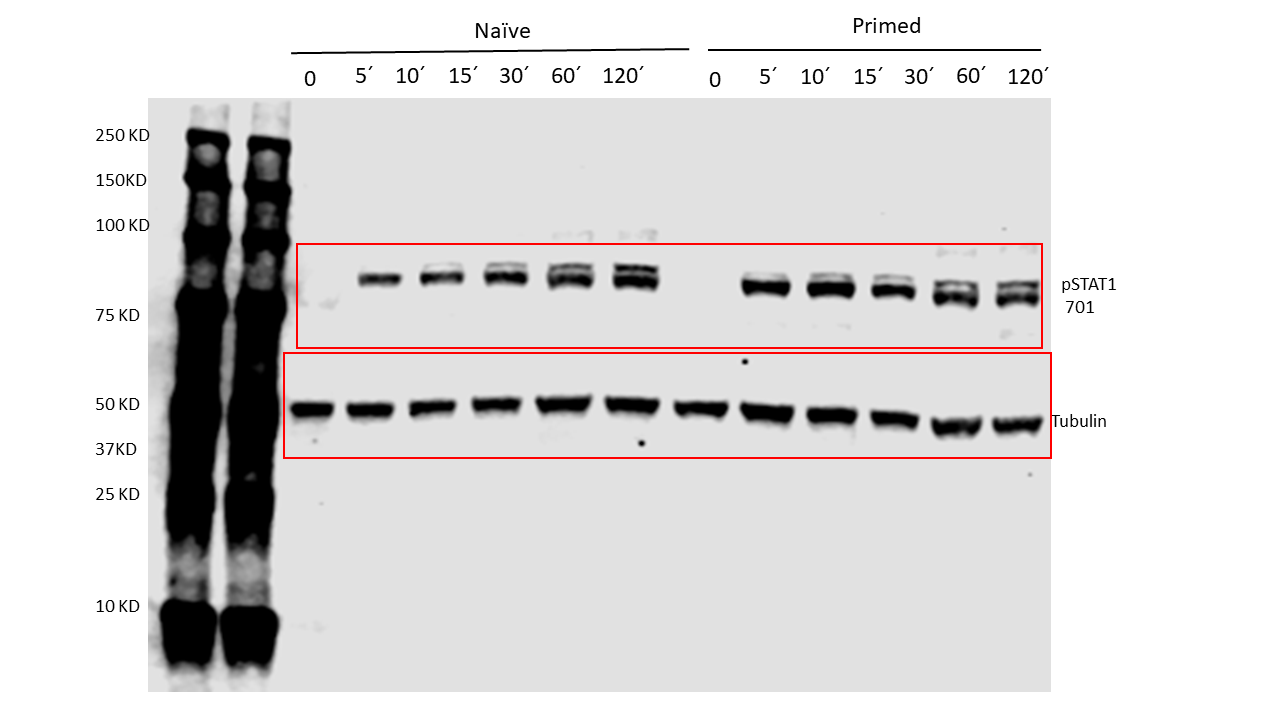

Supplement: Supplementary file 9 — Source Data for Figure 5 [file EMBJ-42-e112259-s007.zip › 5G,H/Westernblot/5G_WB annotation.TIF]

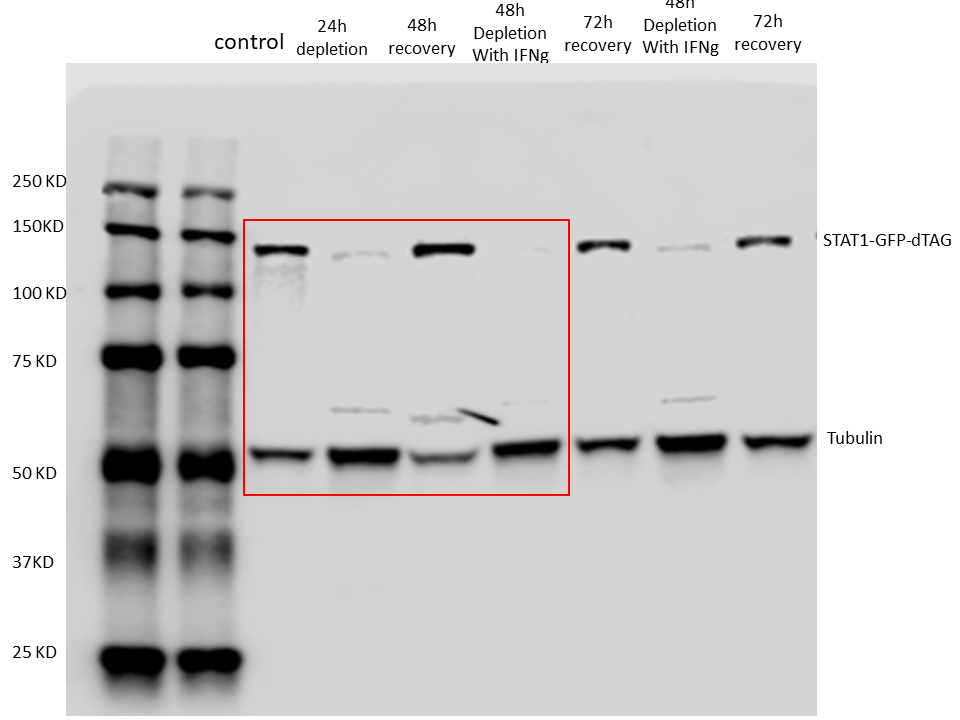

Supplement: Supplementary file 10 — Source Data for Figure 6 [file EMBJ-42-e112259-s009.zip › 6E/westernblot/6E.STAT1dtag.tif]
