# Supplementary material for: STAT1 is required to establish but not maintain interferon‐γ‐induced transcriptional memory
Source: EMBO J. 2023 Jun 5;42(14):e112259. doi: 10.15252/embj.2022112259 (PMC10350821; doi:10.15252/embj.2022112259)
Supplement: Supplementary file 11 [file EMBJ-42-e112259-s001.pdf]

THE EMBO JOURNAL

STAT1 is required to establish but not maintain interferon-γ-induced transcriptional memory

Sahar Tehrani, Pawel Mikulski, Izma Abdul-Zani, João Mata, Wojciech Siwek, and Lars Jansen
DOI: 10.15252/embj.2022112259

Corresponding author(s): Lars Jansen (lars.jansen@bioch.ox.ac.uk) , Wojciech Siwek (siwek@molbio.mgh.harvard.edu)

Review Timeline:

Submission Date:	2nd Aug 22
Editorial Decision:	14th Sep 22
Revision Received:	16th Apr 23
Editorial Decision:	3rd May 23
Revision Received:	9th May 23
Accepted:	12th May 23

Editor: Stefanie Boehm/Karin Dumstrei

Transaction Report:

(Note: With the exception of the correction of typographical or spelling errors that could be a source of ambiguity, letters and reports are not edited. Depending on transfer agreements, referee reports obtained elsewhere may or may not be included in this compilation. Referee reports are anonymous unless the Referee chooses to sign their reports.)

Prof. Lars E.T. Jansen
University of Oxford
Department of Biochemistry
South parks road
Oxford OX1 3QU
United Kingdom

14th Sep 2022

Re: EMBOJ-2022-112259
STAT1 is required to establish but not maintain IFNγ-induced transcriptional memory

Dear Prof. Jansen,
Dear Lars,

Thank you again for submitting your manuscript reporting on STAT1 in the context of IFNgamma-induced transcriptional memory to The EMBO Journal, as well as sending the preliminary response to the two referee reports (included again below). In light of these comments and our discussion, we would now like to formally invite you to prepare and submit a revised manuscript.

As we discussed, in our view, it will be important to provide more insight into the molecular details of transcriptional memory in the context studied here, also with respect to referee #2's concern regarding the depth of molecular mechanism provided. This could for example include addressing and expanding on referee #1's points 3 and 4, and adding experimental data to further define the role of STAT1 phosphorylation and/or involvement of Cdk8. In addition to this crucial point, please also carefully consider all other referee comments and revise the manuscript and figures as needed, as well as providing a detailed response to each comment. Please also remember that the revised manuscript should fulfill all EMBO Journal formatting requirements when it is next submitted (please see below and:
https://www.embopress.org/page/journal/14602075/authorguide#submissionofrevisions)

Please note that it is our policy to allow only a single round of major revision. Acceptance depends on a positive outcome of a second round of review and therefore on the completeness of your responses included in the next, final version of the manuscript. As mentioned, please contact us as early as possible if any issues or questions come up during the revision.

Thank you for the opportunity to consider your work for publication. I look forward to receiving your revised manuscript!

Kind regards,

Stefanie

Stefanie Boehm
Editor
The EMBO Journal

***IMPORTANT NOTE: we now perform an initial quality control of all revised manuscripts before re-review. Your manuscript will FAIL this control and the handling will be DELAYED if the following APPLIES:

1) A data availability section is missing. (The section must be named "Data Availability" and must have the required format, detailed below). Please provide either a referee token or public access to all data.
2) Your manuscript contains error bars based on n=2. Please use scatter blots showing the individual datapoints in these cases. The use of statistical tests needs to be justified.

When submitting your revised manuscript, please carefully review the instructions that follow below. Failure to include requested items will delay the evaluation of your revision.***

When submitting your revised manuscript, please carefully review the instructions below and include the following items:

1) A .docx formatted version of the manuscript text (including legends for main figures, EV figures and tables). Please make sure

that the changes are highlighted to be clearly visible.

2) Individual production quality figure files as .eps, .tif, .jpg (one file per figure). *Please remove figures from the main manuscript*

3) A .docx formatted letter INCLUDING the reviewers' reports and your detailed point-by-point response to their comments. As part of the EMBO Press transparent editorial process, the point-by-point response is part of the Review Process File (RPF), which will be published alongside your paper.

4) A complete author checklist, which you can download from our author guidelines (https://wol-prod-cdn.literatumonline.com/pb-assets/embo-site/Author%20Checklist%20-%20EMBO%20J-1561436015657.xlsx). Please insert information in the checklist that is also reflected in the manuscript. The completed author checklist will also be part of the RPF.

5) All corresponding authors are required to supply an ORCID ID for their name upon submission of a revised manuscript.

6) Before submitting your revision, primary datasets produced in this study need to be deposited in an appropriate public database. The required format for this section is:

Data availability

The datasets (and computer code) produced in this study are available in the following databases:

[data type]: [name of the resource] [accession number/identifier/doi] ([URL or identifiers.org/DATABASE:ACCESSION])

*** Note - All links should resolve to a page where the data can be accessed. ***

Please also see https://www.embopress.org/page/journal/14602075/authorguide#datadeposition Please note that the Data Availability Section is restricted to new primary data that are part of this study.

7) We replaced Supplementary Information with Expanded View (EV) Figures and Tables that are collapsible/expandable online (see examples in http://msb.embopress.org/content/11/6/812). A maximum of 5 EV Figures can be typeset. EV Figures should be cited as 'Figure EV1, Figure EV2" etc.. in the text and their respective legends should be included in the main text after the legends of regular figures.

- For the figures that you do NOT wish to display as Expanded View figures, they should be bundled together with their legends in a single PDF file called *Appendix*, which should start with a short Table of Content. Appendix figures should be referred to in the main text as: "Appendix Figure S1, Appendix Figure S2" etc. See detailed instructions regarding expanded view here: < https://www.embopress.org/page/journal/14602075/authorguide#expandedview>.

- Additional Tables/Datasets should be labeled and referred to as Table EV1, Dataset EV1, etc. Legends have to be provided in a separate tab in case of .xls files. Alternatively, the legend can be supplied as a separate text file (README) and zipped together with the Table/Dataset file.
** We suggest uploading table S1, S2, S3 as datasets. **

8) Our journal encourages inclusion of *data citations in the reference list* to directly cite datasets that were re-used and obtained from public databases. Data citations in the article text are distinct from normal bibliographical citations and should directly link to the database records from which the data can be accessed. In the main text, data citations are formatted as follows: "Data ref: Smith et al, 2001" or "Data ref: NCBI Sequence Read Archive PRJNA342805, 2017". In the Reference list, data citations must be labeled with "[DATASET]". A data reference must provide the database name, accession number/identifiers and a resolvable link to the landing page from which the data can be accessed at the end of the reference. Further instructions are available at < https://www.embopress.org/page/journal/14602075/authorguide#referencesformat >.

9) We would also encourage you to include the source data for figure panels that show essential data. Numerical data can be provided as individual .xls or .csv files (including a tab describing the data). For 'blots' or microscopy, uncropped images should be submitted (using a zip archive or a single pdf per main figure if multiple images need to be supplied for one panel).

Please note that source data should be uploaded in the following format:
a) For main figures: please upload the source data as one zip file per figure and label it as source_data_figX.
b) For EV figures please compile individual (zip) files for each figure, then combine these and upload one zip file for all figures. Please label this as source_data_EVfigures.
c) For Appendix figures please also make one file per figure, combine all Appendix files into one final zip file for all figures and label it as source_data_Appendix.

Additional information on source data and instruction on how to label the files are available at < https://www.embopress.org/page/journal/14602075/authorguide#sourcedata >.

10) When assembling figures, please refer to our figure preparation guideline in order to ensure proper formatting and readability in print as well as on screen:
https://wol-prod-cdn.literatumonline.com/pb-assets/embo-site/EMBOPress_Figure_Guidelines_061115-1561436025777.pdf

Please remember: Digital image enhancement is acceptable practice, as long as it accurately represents the original data and conforms to community standards. If a figure has been subjected to significant electronic manipulation, this must be noted in the figure legend or in the 'Materials and Methods' section. The editors reserve the right to request original versions of figures and the original images that were used to assemble the figure.

Further information is available in our Guide For Authors:
https://www.embopress.org/page/journal/14602075/authorguide

****

We realize that it is difficult to revise to a specific deadline. In the interest of protecting the conceptual advance provided by the work, we recommend a revision within 3 months (13th Dec 2022). Please discuss the revision progress ahead of this time with the editor if you require more time to complete the revisions. Use the link below to submit your revision:

Link Not Available

****

Referee #1:

In a number of systems, subsets of inducible genes exhibit mitotically heritable transcriptional memory, whereby they are more rapidly induced the second time a stimulus is encountered. Work from the Jansen lab and others has shown that interferon gamma induces transcriptional memory at hundreds of genes and that the GBP gene cluster, in particular, shows very long term memory (up to ~15 cell divisions in HeLa cells). Work from yeast has suggested that this type of memory requires interaction with nuclear pore proteins, Mediator kinase (Cdk8) and changes in chromatin modifications such as H3K4me2. Here, the authors explore the molecular requirements for interferon gamma memory. First, they demonstrate that artificially inducing the GBP1 gene using a CRISPRa system is not sufficient to induce memory. This important finding - consistent with the fact that not all interferon gamma-induced genes exhibit memory - highlights the fact that specific factors must control which genes exhibit memory and which do not. Next, the authors show a correlation between memory and increased chromatin accessibility, leading to faster recruitment of transcription factors such as STAT1 and IRF1 to target genes. Constitutive expression of STAT1 is sufficient to induce neither GPB5 expression nor memory, suggesting that other factors (i.e. JAK signaling) regulate STAT1 activity. Furthermore, while conditional depletion of STAT1 during the period between the first and second exposure to interferon gamma had no effect on memory, depletion of STAT1 specifically during the first treatment blocked memory. Thus, STAT1 function is required for the establishment of memory, but it is not required for the persistence/inheritance of memory. Finally, the authors find that phosphorylation of serine 727 of STAT1 is maintained for up to 7 days following treatment with interferon gamma. This modification is critical for STAT1-mediated transcriptional activation and has been suggested to be catalyzed by Cdk8, Mediator kinase, which has been shown to associate with many genes during memory. However, it is unclear if this persistent phosphorylation plays a role in this phenomenon.

Overall, this is an excellent paper. The data are of high quality and the conclusions are sound. Below, I suggest several experiments that would strengthen the conclusions or validate aspects of their model.
1. The protein levels of STAT1 and IRF1 should be measured in naïve and primed cells to validate the mRNA measurements.
2. The interpretation of the effects of artificially expressing GBP1 would be strengthened by a control experiment in which cells are both transfected with the CRISPRa-SAM construct driving expression of GBP1 and treated with IFN gamma.
3. The connection between S272 phophorylation and memory would be strengthened if expression of a phosphomimetic S272D STAT1 led to constitutive memory.
4. Testing if Cdk8 remains associated with the promoters/enhancers of IFN gamma induced genes that exhibit memory would

strengthen the model that S272 phosphorylation is important for memory. Alternatively, inhibiting Cdk8 and showing that this phosphorylation is lost and leads to a defect in memory would strengthen this idea.

Referee #2:

The manuscript from Tehrani et al. describes molecular bases of transcriptional memory in HeLa cells treated with Interferon-γ. The authors previously reported that clustered GBP genes were induced faster/greater upon 2nd stimulation of IFN-γ.
The authors found that STAT1, an important transcription factor for induction of Interferon stimulated genes, is essential to establish transcriptional memory of GBP genes. They showed that transcription itself did not show transcriptional memory at GBP cluster, but relies upon STAT1, by knock-out/knock-in system using CRISPR method and inducible deletion of STAT1 by a destruction tag (dTAG). They also found that specific phosphorylation of STAT1 remains phosphoryated during transcriptional memory. However, the role of STAT1 phosphorylation on transcriptional memory remains unclear.
This study is potentially interesting, however, there are significant flaws in the lack of data and the molecular mechanisms of transcriptional memory remains obscure, that preclude publication in EMBO J.

Specific comments are indicated below:

Major points:
1. Constitutive STAT1 expressed HeLa cells showed weaker memory (low expression level of GBP5) compared with parental HeLa cells, which have IFN inducible STAT1 (Figure 5D). The authors should discuss about this phenomenon/ mechanism.

2. Figure 5C. Expression levels of endogenous IFN-inducible STAT1 (parental cels) and constitutively expressed STAT1 were similar. However, the band of constitutive expressed STAT1 protein was lower than IFN-inducible endogenous STAT1. Is there any difference of post-translational modifications (phosphorylation status) of STAT1 including S727, which remains phosphorylated during priming? The authors should discuss the results.

3. Figure 6C. The authors showed that STAT1 protein was deleted by dTAG13 addition at 24 hrs time point. However, the author did not show detailed time course of STAT1 expression level during dTAG13 treatment (48 h ~ 120 h before re-induction). To conclude that STAT1 protein is dispensable during memory of the primed state, more detailed analysis should be performed.

Referee #1 (Report for Author)

In a number of systems, subsets of inducible genes exhibit mitotically heritable transcriptional memory, whereby they are more rapidly induced the second time a stimulus is encountered. Work from the Jansen lab and others has shown that interferon gamma induces transcriptional memory at hundreds of genes and that the GBP gene cluster, in particular, shows very long term memory (up to ~15 cell divisions in HeLa cells). Work from yeast has suggested that this type of memory requires interaction with nuclear pore proteins, Mediator kinase (Cdk8) and changes in chromatin modifications such as H3K4me2. Here, the authors explore the molecular requirements for interferon gamma memory. First, they demonstrate that artificially inducing the GBP1 gene using a CRISPRa system is not sufficient to induce memory. This important finding - consistent with the fact that not all interferon gamma-induced genes exhibit memory - highlights the fact that specific factors must control which genes exhibit memory and which do not. Next, the authors show a correlation between memory and increased chromatin accessibility, leading to faster recruitment of transcription factors such as STAT1 and IRF1 to target genes. Constitutive expression of STAT1 is sufficient to induce neither GPB5 expression nor memory, suggesting that other factors (i.e. JAK signaling) regulate STAT1 activity. Furthermore, while conditional depletion of STAT1 during the period between the first and second exposure to interferon gamma had no effect on memory, depletion of STAT1 specifically during the first treatment blocked memory. Thus, STAT1 function is required for the establishment of memory, but it is not required for the persistence/inheritance of memory. Finally, the authors find that phosphorylation of serine 727 of STAT1 is maintained for up to 7 days following treatment with interferon gamma. This modification is critical for STAT1-mediated transcriptional activation and has been suggested to be catalyzed by Cdk8, Mediator kinase, which has been shown to associate with many genes during memory. However, it is unclear if this persistent phosphorylation plays a role in this phenomenon.

Overall, this is an excellent paper. The data are of high quality and the conclusions are sound. Below, I suggest several experiments that would strengthen the conclusions or validate aspects of their model.

We thank the reviewer for their positive evaluation of our work. We have carefully considered and addressed the reviewer's comments and suggestions below.

1. The protein levels of STAT1 and IRF1 should be measured in naïve and primed cells to validate the mRNA measurements.

The reviewer raises a valid point to demonstrate STAT1 and IRF1 protein levels alongside the mRNA data presented in Figure 3A. We have now performed these experiments and found that protein levels generally reflect mRNA levels. This data is now included in the new Figure EV3D.

2. The interpretation of the effects of artificially expressing GBP1 would be strengthened by a control experiment in which cells are both transfected with the CRISPRa-SAM construct driving expression of GBP1 and treated with IFN gamma.

We thank the reviewer for suggesting this experiment that we agree is a valuable additional control. We have shown in the original submission that targeted transcription of the GBP1 gene is not sufficient to prime the gene for enhanced expression following a memory period. However, while not sufficient by itself, it is possible that combining CRISPR activation with interferon will synergize to activate the target gene and possibly lead to stronger priming.

We have now redone these experiments including the control the reviewer requested. As in our original submission, CRISPR activation targeted to GBP1 drives specific GBP1 expression to similar levels as those induced by IFNγ (new Figure 2B)

and does not by itself prime GBP1 (new Fig 2C). Interestingly, combining CRISPRa and IFNγ has an additive effect on expression (new Fig. 2B). However, we find that this does not lead to stronger priming (new Fig. 2D). In sum, we find that CRISPRa alone does not prime cells while IFNγdoes (as in our original submission) but also that when CRISPRa is combined with interferon it does not lead to any further priming, beyond what is induced by interferon. This is consistent with our initial conclusion that mere transcription does not contribute to priming of GBP1. We used a non-relevant gene as control (ASCL1) as well as no CRISPRa. While these different treatments caused some variation in induction and priming, the difference between GBP1 activation, negative control gene activation and no CRISPRa are not significant. This new data is now included in new figures 2B-D and discussed in lines 134-148.

3. The connection between S272 phophorylation and memory would be strengthened if expression of a phosphomimetic S272D STAT1 led to constitutive memory.

We showed in our original submission that while S727 phosphorylation is maintained for up to 7 days post priming, we find that fully removing STAT1 during this window without any effect on memory. This indicates that STAT1 is not the carrier of memory. Nevertheless, the reviewer is correct that despite STAT1 not being the carrier of memory, constitutively activated STAT1 could drive rapid reactivation.

We have now performed the requested experiment and have generated cells in which we made two phosphomimetic mutations at the S727 site to E and D. We expressed these in a STAT1 knockout background similar to the constitutively expressing STAT1 cells in the experiments shown Figure 5.

We find that these phosphomimetic mutants, unlike the non-phosphorylated mutant are still able to robustly support GBP expression (new figure 6B,C) indicating that S727 phosphorylation is required for expression. However, the addition of charged residues does not lead to enhanced memory. Interestingly, priming is reduced (see memory ratios in Figure 6C). These new results suggest that the differential phosphorylation of this residue between naïve cells (no S727 phosphorylation) and primed cells (memorized S272 phosphorylation) is functionally relevant for memory but may dependent on the dynamic of control of the phosphorylated state that is difficult to capture with constitutive mutations. Nevertheless, we hope the reviewer agrees that this experiment is a valuable addition to the manuscript that we discuss in lines 295-305.

4. Testing if Cdk8 remains associated with the promoters/enhancers of IFN gamma induced genes that exhibit memory would strengthen the model that S272 phosphorylation is important for memory. Alternatively, inhibiting Cdk8 and showing that this phosphorylation is lost and leads to a defect in memory would strengthen this idea.

We agree with the reviewer that a putative role for Cdk8 in memory is potentially interesting as it has been implicated previously in both yeast *INO1* memory and human interferon memory (D'Urso et al., (2016) *eLife*), Upon the reviewers request we have attempted to perform siRNA-mediated depletion and pharmacological inhibition of Cdk8 in the context of our interferon induction and reinduction

experiments. However, the results are not easily interpretable where we see both up and downregulation of genes in ways that do not clearly correlate with interferon-induced expression. This likely reflects the broad requirement for Cdk8 and the associated mediator complex in transcription and manipulating its activity has pleiotropic effects. For this reason, we feel these experiments are inadequate to test the reviewers' hypothesis and we prefer not to include these as they do not add any further insight.

As our paper is largely focused on the specific role of STAT1 in transcriptional memory we have not performed further ChIP experiments to test Cdk8 association as we feel this takes the paper further from its orifical scope. We nevertheless hope that our addition of additional western blots, CRISPRa control experiments and experiments on STAT1 phosphomimetic mutants address the key concerns of the reviewer.

Referee #2 (Report for Author)

The manuscript from Tehrani et al. describes molecular bases of transcriptional memory in HeLa cells treated with Interferon-γ. The authors previously reported that clustered GBP genes were induced faster/greater upon 2nd stimulation of IFN-γ.
The authors found that STAT1, an important transcription factor for induction of Interferon stimulated genes, is essential to establish transcriptional memory of GBP genes. They showed that transcription itself did not show transcriptional memory at GBP cluster, but relies upon STAT1, by knock-out/knock-in system using CRISPR method and inducible deletion of STAT1 by a destruction tag (dTAG). They also found that specific phosphorylation of STAT1 remains phosphoryated during transcriptional memory. However, the role of STAT1 phosphorylation on transcriptional memory remains unclear.
This study is potentially interesting, however, there are significant flaws in the lack of data and the molecular mechanisms of transcriptional memory remains obscure, that preclude publication in EMBO J.

We thank the reviewer for their assessment that our paper is of interest. We fully agree that the role of STAT1 phosphorylation remains an open question and the overall mechanism of memory remains unclear. Nevertheless, we have made some key observations that we think are significant and important to move the field forward. Our finding that STAT1 is essential for gene priming in a manner that is independent of direct transcriptional activation is a valuable advance. Further, in response to reviewer 1, we have also added additional controls to further strengthen our observation that gene activation per se is not sufficient for priming. We also added new data in response to reviewer 1 showing that phosphomimetic mutants of STAT1, while functional in activating GBP expression, have reduced priming.

Further, we value the reviewers' additional comments that we address in more detail below.

Specific comments are indicated below:

Major points:
1. Constitutive STAT1 expressed HeLa cells showed weaker memory (low expression level of GBP5) compared with parental HeLa cells, which have IFN inducible STAT1 (Figure 5D). The authors should discuss about this phenomenon/ mechanism.

We agree that there appears to be an overall lower level of target gene expression between the endogenous and constitutively expressing STAT1 cells and we failed to discuss this. The main point of this experiment is to show that even if STAT1 is no longer under interferon-gamma control (i.e. constitutively expressed), cells still maintain normal transcriptional memory. Thus, while overall expression levels may be somewhat lower, memory is maintained despite loss of regulated STAT1 expression.

Nevertheless, the reviewer is correct that we did not discuss why the overall GBP5 gene expression levels are lower than the parental cells. It is possible that the STAT1 expression levels are slightly lower in the constitutively expressed cells, leading to lower transcriptional output. This may also be related to the specific isoform that we are expressing (see also the reviewers' next point about STAT1 size). We have now performed these experiments again in different STAT1-expressing clones and found that GBP5 expression is consistently lower (see Appendix Fig S2). Importantly, memory is maintained despite the loss of STAT1 regulation which is the main point of the experiment (see quantification in Appendix Fig S2B) We have now added a discussion on this point in lines 237-245.

2. Figure 5C. Expression levels of endogenous IFN-inducible STAT1 (parental cels) and constitutively expressed STAT1 were similar. However, the band of constitutive expressed STAT1 protein was lower than IFN-inducible endogenous STAT1. Is there any difference of post-translational modifications (phosphorylation status) of STAT1 including S727, which remains phosphorylated during priming? The authors should discuss the results.

The reviewer raises a valid point and indeed it appears that the STAT1 sizes are slightly different. We have reassessed multiple western blots and ascertained that indeed our transgenic STAT1 levels are slightly slower migrating. Endogenous STAT1 is expressed as multiple isoforms and we cloned and constitutively expressed the main isoform. This may perhaps explain some of the differences in molecular weight and the level of GBP5 expression (see reviewers' point above). We have added a description on this point in lines 239-242. Nevertheless, despite the possible differences in isoform expression the STAT1 transgene is fully functional and supports GBP expression and transcriptional memory which are the functions we aim to characterize in this manuscript.

3. Figure 6C. The authors showed that STAT1 protein was deleted by dTAG13 addition at 24 hrs time point. However, the author did not show detailed time course of STAT1 expression level during dTAG13 treatment (48 h ~ 120 h before re-induction). To conclude that STAT1 protein is dispensable during memory of the primed state, more detailed analysis should be performed.

The reviewer points out what appears to be poor figure labeling on our part. The measurements of the time points that the reviewer requests are in fact in the figure but are numbered differently from what is perhaps more logical. We have now updated Figure 6E and the legend to make this clearer. Our data shows that we can fully deplete STAT1 with 24h of dTAG13 and STAT1 levels fully recover 48 hours after washout prior to reinduction with interferon.

1st Revision - Editorial Decision 3rd May 2023

Dear Lars,

Thank you for submitting your revised manuscript to The EMBO Journal. Your study has now been seen by the two referees and their comments are provide below.

As you can see both referees appreciate the added changes and support publication here.

Before proceeding with acceptance here, there are some editorial points that we need to sort out including some figure issues.

When you submit the revised manuscript, please address the following points:

The funding information for LETJ - University of Oxford, Department of Biochemistry and St Edmund Hall should be added into the online submission system.

Please make sure that the deposited data (Data Availability section) is open and accessible.

COI needs to be re-labelled "Disclosure Statement and Competing Interests"

Please remove the Authors Contributions from the manuscript. The 'Author Contributions' section is replaced by the CRediT contributor roles taxonomy to specify the contributions of each author in the journal submission system. Please use the free text box in the 'author information' section of the manuscript submisssion system to provide more detailed descriptions (e.g., 'X provided intracellular Ca++ measurements in fig Y')

There is a Supplementary Table 1 called out in the text, but it appears to be missing.

The resource table should be removed from the MS and uploaded separately

Please upload a synopsis text. We need a summary statement plus 3-5 bullet points describing the key findings of the MS.

We also need a synopsis image => 550 wide by [200-400]

Our publisher has also done their pre-publication check on your manuscript. When you log into the manuscript submission system you will see the file "Data Edited Manuscript file". Please take a look at the word file and the comments regarding the figure legends and respond to the issues.

We have noted a few figure issues:

Is the GBPS panel from Figure 1C re-used in Figure 3B and Figure EV3B?

Is the GBPS & Tubulin panel from Figure 1C re-used in Figure EV3C? PS you mention Figure 1A in the EV3C figure legend, but shouldn't it be 1C?

Is the GPBS panel reused in Figure EV3B & EV3C from Figure 1C?

Is the Tubulin panel from Figure 3B reused in EV Figure 3B?

Is the GBPS and Tubulin panel and second GBPS from Figure 5D reused in Appendix Fig. S2?

Is the GBPS panel reused within Figure EV3 B and C?

If any of the panels are re-used in multiple figures, please state that in the figure legend.

When you re-submit the revision please include a point-by-point response

best Karin

Karin Dumstrei, PhD
Senior Editor
The EMBO Journal

Instructions for preparing your revised manuscript:

Guide For Authors: https://www.embopress.org/page/journal/14602075/authorguide

----

Referee #1:

The authors have addressed my minor concerns and responded positively to my suggested additional data. I have no concerns.

Referee #2:

The authors have addressed all my concerns in the revised manuscript and I have no further comments.

Karin Dumstrei, PhD
Senior Editor
The EMBO Journal

Manuscript EMBOJ-2022-112259R, final revision

Oxford, UK, 9th of May, 2023

Dear Karin,

Thank you for your decision to move forward with our manuscript. We have now submitted a revised manuscript and figures in which we addressed the points raised in your decision letter.

Below, I have outlined our changes to the manuscript point by point:

The funding information for LETJ - University of Oxford, Department of Biochemistry and St Edmund Hall should be added into the online submission system.
This information has now been added to the submission system.

Please make sure that the deposited data (Data Availability section) is open and accessible.
The externally deposited data is now accessible.

COI needs to be re-labelled "Disclosure Statement and Competing Interests"
This has been relabeled.

Please remove the Authors Contributions from the manuscript. The 'Author Contributions' section is replaced by the CRediT contributor roles taxonomy to specify the contributions of each author in the journal submission system. Please use the free text box in the 'author information' section of the manuscript submisssion system to provide more detailed descriptions (e.g., 'X provided intracellular Ca++ measurements in fig Y')
We have removed the contributions from the main text and updated the contributions in the submission system.

There is a Supplementary Table 1 called out in the text, but it appears to be missing.
This was mislabeled as Supplementary Table which should have been Appendix Table S1. This is now updated.

The resource table should be removed from the MS and uploaded separately
This is removed and a separate file is provided.

Lars E.T. Jansen
Department of Biochemistry
University of Oxford
South Parks Road
Oxford OX1 3QU
United Kingdom

Room: 50-021
Office phone: +44 (0)18656-13428
lars.jansen@bioch.ox.ac.uk
www.jansenlab.org

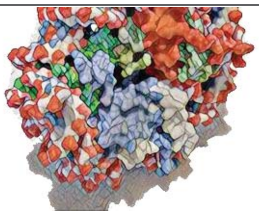

Please upload a synopsis text. We need a summary statement plus 3-5 bullet points describing the key findings of the MS.
This is now included in the submission.

We also need a synopsis image => 550 wide by [200-400]
This is now included in the submission.

Our publisher has also done their pre-publication check on your manuscript. When you log into the manuscript submission system you will see the file "Data Edited Manuscript file". Please take a look at the word file and the comments regarding the figure legends and respond to the issues.
We have addressed all the issues raised. These are related to the type of statistical test used and the number of replicates. This is now clarified in all cases.

We have noted a few figure issues:

Is the GBPS panel from Figure 1C re-used in Figure 3B and Figure EV3B?
This is the same data that we included in 3B to facilitate comparison. We have clarified this now in the legend.

Is the GBPS & Tubulin panel from Figure 1C re-used in Figure EV3C? PS you mention Figure 1A in the EV3C figure legend, but shouldn't it be 1C?
This is the same data for the wild-type cells (parental) that we included in EV3C to facilitate comparison. We have clarified this now in the legend. We refer to Figure 1A in EV3C, not Figure 1C as we refer to the experimental scheme that was followed in the experiment in EV3C. The original figure citation is correct, and we left it as is.

Is the GPBS panel reused in Figure EV3B & EV3C from Figure 1C?
Yes, we included the data for wild-type cells (parent) from 1C here to facilitate comparison. We have clarified this in the legend.

Is the Tubulin panel from Figure 3B reused in EV Figure 3B?
Yes, for the same reason as above. We have clarified this in the legend.

Is the GBPS and Tubulin panel and second GBPS from Figure 5D reused in Appendix Fig. S2?
Yes, Figure S2 is an expansion of Figure 5D where we show additional data but also the original data in 5D to allow comparison. We have clarified this in the legend.

Is the GBPS panel reused within Figure EV3 B and C?
Yes, for the same reason as above. We have clarified this in the legend.

If any of the panels are re-used in multiple figures, please state that in the figure legend.
This has now been clarified for all instances.

Note that we have also rechecked the identity of the western blots and ensured formatting of the blots matches that of the raw data included in the source data.

Note also that we have updated the data plots to include scatter plots showing the individual data points as requested in the original decision letter.

Lars E.T. Jansen
Department of Biochemistry
University of Oxford
South Parks Road
Oxford OX1 3QU
United Kingdom

Room: 50-021
Office phone: +44 (0)18656-13428
lars.jansen@bioch.ox.ac.uk
www.jansenlab.org

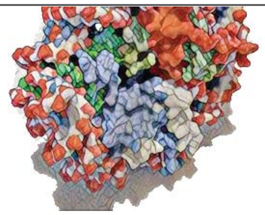

I hope our revision has covered all the points raised.
Do let me know if you need any further clarification.

With best wishes,

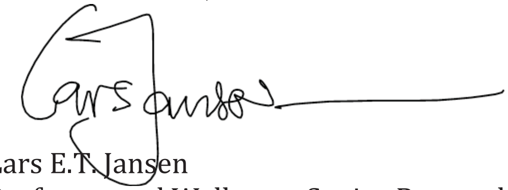

Lars E.T. Jansen
Professor and Wellcome Senior Research Fellow
Department of Biochemistry, University of Oxford

Lars E.T. Jansen
Department of Biochemistry
University of Oxford
South Parks Road
Oxford OX1 3QU
United Kingdom

Room: 50-021
Office phone: +44 (0)18656-13428
lars.jansen@bioch.ox.ac.uk
www.jansenlab.org

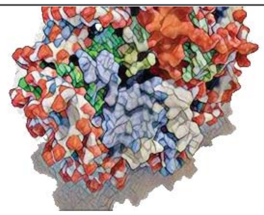

2nd Revision - Editorial Decision 12th May 2023

Dear Lars,

Thank you for sending us your revised manuscript. I have now looked at everything carefully and all looks good.

I am therefore very pleased to accept the manuscript for publication here.

Congratulations on a nice study!

Best Karin

Karin Dumstrei, PhD
Senior Editor
The EMBO Journal

Please note that it is EMBO Journal policy for the transcript of the editorial process (containing referee reports and your response letter) to be published as an online supplement to each paper. If you do NOT want this, you will need to inform the Editorial Office via email immediately. More information is available here: https://www.embopress.org/page/journal/14602075/authorguide#transparentprocess

Your manuscript will be processed for publication in the journal by EMBO Press. Manuscripts in the PDF and electronic editions of The EMBO Journal will be copy edited, and you will be provided with page proofs prior to publication. Please note that supplementary information is not included in the proofs.

You will be contacted by Wiley Author Services to complete licensing and payment information. The required 'Page Charges Authorization Form' is available here: https://www.embopress.org/pb-assets/embo-site/tej_apc.pdf - please download and complete the form and return to embopressproduction@wiley.com

EMBO Press participates in many Publish and Read agreements that allow authors to publish Open Access with reduced/no publication charges. Check your eligibility: https://authorservices.wiley.com/author-resources/Journal-Authors/open-access/affiliation-policies-payments/index.html

Should you be planning a Press Release on your article, please get in contact with embojournal@wiley.com as early as possible, in order to coordinate publication and release dates.

If you have any questions, please do not hesitate to call or email the Editorial Office. Thank you for your contribution to The EMBO Journal.

EMBO Press Author Checklist

Corresponding Author Name: Wojciech Siwek and Lars E.T. Jansen
Journal Submitted to: The EMBO Journal
Manuscript Number: EMBOJ-2022-112259

USEFUL LINKS FOR COMPLETING THIS FORM
The EMBO Journal - Author Guidelines
EMBO Reports - Author Guidelines
Molecular Systems Biology - Author Guidelines
EMBO Molecular Medicine - Author Guidelines

Reporting Checklist for Life Science Articles (updated January
This checklist is adapted from Materials Design Analysis Reporting (MDAR) Checklist for Authors. MDAR establishes a minimum set of requirements in transparent reporting in the life sciences (see Statement of Task: 10.31222/osf.io/9sm4x). Please follow the journal's guidelines in preparing your manuscript.
Please note that a copy of this checklist will be published alongside your article.

Abridged guidelines for figures
1. Data
The data shown in figures should satisfy the following conditions:
- the data were obtained and processed according to the field's best practice and are presented to reflect the results of the experiments in an accurate and unbiased manner.
- ideally, figure panels should include only measurements that are directly comparable to each other and obtained with the same assay.
- plots include clearly labeled error bars for independent experiments and sample sizes. Unless justified, error bars should not be shown for technical
- if n<5, the individual data points from each experiment should be plotted. Any statistical test employed should be justified.
- Source Data should be included to report the data underlying figures according to the guidelines set out in the authorship guidelines on Data

2. Captions
Each figure caption should contain the following information, for each panel where they are relevant:
- a specification of the experimental system investigated (eg cell line, species name).
- the assay(s) and method(s) used to carry out the reported observations and measurements.
- an explicit mention of the biological and chemical entity(ies) that are being measured.
- an explicit mention of the biological and chemical entity(ies) that are altered/varied/perturbed in a controlled manner.
- the exact sample size (n) for each experimental group/condition, given as a number, not a range;
- a description of the sample collection allowing the reader to understand whether the samples represent technical or biological replicates (including how many animals, litters, cultures, etc.).
- a statement of how many times the experiment shown was independently replicated in the laboratory.
- definitions of statistical methods and measures:
  - common tests, such as t-test (please specify whether paired vs. unpaired), simple χ^2 tests, Wilcoxon and Mann-Whitney tests, can be unambiguously identified by name only, but more complex techniques should be described in the methods section;
  - are tests one-sided or two-sided?
  - are there adjustments for multiple comparisons?
  - exact statistical test results, e.g., P values = x but not P values < x;
  - definition of 'center values' as median or average;
  - definition of error bars as s.d. or s.e.m.

Please complete ALL of the questions below.
Select "Not Applicable" only when the requested information is not relevant for your study.

Materials

Newly Created Materials	Information included in the manuscript?	In which section is the information available? (Reagents and Tools Table, Materials and Methods, Figures, Data Availability Section)
New materials and reagents need to be available; do any restrictions apply?	Not Applicable	

Antibodies	Information included in the manuscript?	In which section is the information available? (Reagents and Tools Table, Materials and Methods, Figures, Data Availability Section)
For **antibodies** provide the following information:<br>- Commercial antibodies: RRID (if possible) or supplier name, catalogue number and or/clone number<br>- Non-commercial: RRID or citation	Yes	Material and Methods (Resource table)

DNA and RNA sequences	Information included in the manuscript?	In which section is the information available? (Reagents and Tools Table, Materials and Methods, Figures, Data Availability Section)
**Short novel DNA or RNA including primers, probes**: provide the sequences.	Yes	Material and Methods (DNA constructs and genome engineering) and Appendix table S1

Cell materials	Information included in the manuscript?	In which section is the information available? (Reagents and Tools Table, Materials and Methods, Figures, Data Availability Section)
**Cell lines:** Provide species information, strain. Provide accession number in repository **OR** supplier name, catalog number, clone number, and/**OR** RRID.	Yes	Material and Methods (Resource table)
**Primary cultures:** Provide species, strain, sex of origin, genetic modification status.	Not Applicable	
Report if the cell lines were recently **authenticated** (e.g., by STR profiling) and tested for mycoplasma contamination.	Yes	Material and Methods

Experimental animals	Information included in the manuscript?	In which section is the information available? (Reagents and Tools Table, Materials and Methods, Figures, Data Availability Section)
**Laboratory animals or Model organisms:** Provide species, strain, sex, age, genetic modification status. Provide accession number in repository **OR** supplier name, catalog number, clone number, **OR** RRID.	Not Applicable	
**Animal observed in or captured from the field:** Provide species, sex, and age where possible.	Not Applicable	
Please detail **housing and husbandry conditions**.	Not Applicable	

Plants and microbes	Information included in the manuscript?	In which section is the information available? (Reagents and Tools Table, Materials and Methods, Figures, Data Availability Section)
**Plants:** provide species and strain, ecotype and cultivar where relevant, unique accession number if available, and source (including location for collected wild specimens).	Not Applicable	
**Microbes:** provide species and strain, unique accession number if available, and source.	Yes	Material and Methods (Resource table)

Human research participants	Information included in the manuscript?	In which section is the information available? (Reagents and Tools Table, Materials and Methods, Figures, Data Availability Section)
If collected and within the bounds of privacy constraints report on age, sex and gender or ethnicity for all study participants.	Not Applicable	

Core facilities	Information included in the manuscript?	In which section is the information available? (Reagents and Tools Table, Materials and Methods, Figures, Data Availability Section)
If your work benefited from core facilities, was their service mentioned in the acknowledgments section?	Yes	

Design

Study protocol	Information included in the manuscript?	In which section is the information available? (Reagents and Tools Table, Materials and Methods, Figures, Data Availability Section)
If study protocol has been **pre-registered, provide DOI in the manuscript**. For clinical trials, provide the trial registration number **OR** cite DOI.	Not Applicable	
Report the **clinical trial registration number** (at ClinicalTrials.gov or equivalent), where applicable.	Not Applicable	

Laboratory protocol	Information included in the manuscript?	In which section is the information available? (Reagents and Tools Table, Materials and Methods, Figures, Data Availability Section)
Provide DOI OR other citation details if **external detailed step-by-step protocols** are available.	Yes	Material and Methods

Experimental study design and statistics	Information included in the manuscript?	In which section is the information available? (Reagents and Tools Table, Materials and Methods, Figures, Data Availability Section)
Include a statement about **sample size** estimate even if no statistical methods were used.	Yes	Material and Methods (Quantification and statistical analysis) and Figure legends
Were any steps taken to minimize the effects of subjective bias when allocating animals/samples to treatment (e.g. **randomization procedure**)? If yes, have they been described?	Not Applicable	
Include a statement about **blinding** even if no blinding was done.	Not Applicable	
Describe **inclusion/exclusion criteria** if samples or animals were excluded from the analysis. Were the criteria pre-established?<br><br>If sample or data points were omitted from analysis, report if this was due to attrition or intentional exclusion and provide justification.	Not Applicable	
For every figure, are **statistical tests** justified as appropriate? Do the data meet the assumptions of the tests (e.g., normal distribution)? Describe any methods used to assess it. Is there an estimate of variation within each group of data? Is the variance similar between the groups that are being statistically compared?	Yes	Material and Methods (Quantification and statistical analysis)

Sample definition and in-laboratory replication	Information included in the manuscript?	In which section is the information available? (Reagents and Tools Table, Materials and Methods, Figures, Data Availability Section)
In the figure legends: state number of times the experiment was **replicated** in laboratory.	Yes	
In the figure legends: define whether data describe **technical or biological replicates**.	Yes	

Ethics

Ethics	Information included in the manuscript?	In which section is the information available? (Reagents and Tools Table, Materials and Methods, Figures, Data Availability Section)
Studies involving **human participants**: State details of **authority granting ethics approval** (IRB or equivalent committee(s), provide reference number for approval.	Not Applicable	
Studies involving **human participants**: Include a statement confirming that **informed consent** was obtained from all subjects and that the experiments conformed to the principles set out in the WMA Declaration of Helsinki and the Department of Health and Human Services Belmont Report.	Not Applicable	
Studies involving **human participants:** For publication of **patient photos**, include a statement confirming that consent to publish was obtained.	Not Applicable	
Studies involving experimental **animals**: State details of **authority granting ethics approval** (IRB or equivalent committee(s), provide reference number for approval. Include a statement of compliance with ethical regulations.	Not Applicable	
Studies involving **specimen and field samples:** State if relevant **permits** obtained, provide details of authority approving study; if none were required, explain why.	Not Applicable	

Dual Use Research of Concern (DURC)	Information included in the manuscript?	In which section is the information available? (Reagents and Tools Table, Materials and Methods, Figures, Data Availability Section)
Could your study fall under dual use research restrictions? Please check biosecurity documents and list of **select agents and toxins** (CDC): https://www.selectagents.gov/sat/list.htm	Not Applicable	
If you used a select agent, is the security level of the lab appropriate and reported in the manuscript?	Not Applicable	
If a study is subject to dual use research of concern regulations, is the name of the **authority granting approval and reference number** for the regulatory approval provided in the manuscript?	Not Applicable	

Reporting

The MDAR framework recommends adoption of discipline-specific guidelines, established and endorsed through community initiatives. Journals have their own policy about requiring specific guidelines and recommendations to complement MDAR.

Adherence to community standards	Information included in the manuscript?	In which section is the information available? (Reagents and Tools Table, Materials and Methods, Figures, Data Availability Section)
State if relevant guidelines or checklists (e.g., **ICMJE, MIBBI, ARRIVE, PRISMA**) have been followed or provided.	Not Applicable	
For **tumor marker prognostic studies,** we recommend that you follow the **REMARK** reporting guidelines (see link list at top right). See author guidelines, under 'Reporting Guidelines'. Please confirm you have followed these guidelines.	Not Applicable	
For **phase II and III randomized controlled trials**, please refer to the **CONSORT** flow diagram (see link list at top right) and submit the CONSORT checklist (see link list at top right) with your submission. See author guidelines, under 'Reporting Guidelines'. Please confirm you have submitted this list.	Not Applicable	

Data Availability

Data availability	Information included in the manuscript?	In which section is the information available? (Reagents and Tools Table, Materials and Methods, Figures, Data Availability Section)
Have **primary datasets** been deposited according to the journal's guidelines (see 'Data Deposition' section) and the respective accession numbers provided in the Data Availability Section?	Yes	Data availability
Were **human clinical and genomic datasets** deposited in a public access-controlled repository in accordance to ethical obligations to the patients and to the applicable consent agreement?	Yes	Data availability
Are **computational models** that are central and integral to a study available without restrictions in a machine-readable form? Were the relevant accession numbers or links provided?	Not Applicable	
If publicly available data were reused, provide the respective **data citations in the reference list**.	Yes	Figure legends
